# Micronutrient supplementation affects DNA methylation in male gonads with potential intergenerational epigenetic inheritance involving the embryonic development through glutamate receptor-associated genes

**DOI:** 10.1186/s12864-022-08348-4

**Published:** 2022-02-10

**Authors:** Takaya Saito, Paul Whatmore, John F. Taylor, Jorge M. O. Fernandes, Anne-Catrin Adam, Douglas R. Tocher, Marit Espe, Kaja H. Skjærven

**Affiliations:** 1 Institute of Marine Research, Bergen, Norway; 2 Institute of Aquaculture, Faculty of Natural Sciences, University of Stirling, Scotland, UK; 3 Faculty of Biosciences and Aquaculture, Nord University, Bodø, Norway

**Keywords:** Atlantic salmon, Micronutrient, Epigenetics, DNA methylation, Intergenerational inheritance, Male germline, Gonad, Glutamate receptor, GRIN3A, HDAC2

## Abstract

**Background:**

DNA methylation has an important role in intergenerational inheritance. An increasing number of studies have reported evidence of germline inheritance of DNA methylation induced by nutritional signals in mammals. Vitamins and minerals as micronutrients contribute to growth performance in vertebrates, including Atlantic salmon (*Salmo salar*), and also have a role in epigenetics as environmental factors that alter DNA methylation status. It is important to understand whether micronutrients in the paternal diet can influence the offspring through alterations of DNA methylation signatures in male germ cells.

**Results:**

Here, we show the effect of micronutrient supplementation on DNA methylation profiles in the male gonad through a whole life cycle feeding trial of Atlantic salmon fed three graded levels of micronutrient components. Our results strongly indicate that micronutrient supplementation affects the DNA methylation status of genes associated with cell signalling, synaptic signalling, and embryonic development. In particular, it substantially affects DNA methylation status in the promoter region of a glutamate receptor gene, *glutamate receptor ionotropic, NMDA 3A-like* (*grin3a-like*), when the fish are fed both medium and high doses of micronutrients. Furthermore, two transcription factors, *histone deacetylase 2* (*hdac2*) and a zinc finger protein, bind to the hyper-methylated site in the *grin3a-like* promoter. An estimated function of *hdac2* together with a zinc finger indicates that *grin3a-like* has a potential role in intergenerational epigenetic inheritance and the regulation of embryonic development affected by paternal diet.

**Conclusions:**

The present study demonstrates alterations of gene expression patterns and DNA methylation signatures in the male gonad when Atlantic salmon are fed different levels of micronutrients. Alterations of gene expression patterns are of great interest because the gonads are supposed to have limited metabolic activities compared to other organs, whereas alterations of DNA methylation signatures are of great importance in the field of nutritional epigenetics because the signatures affected by nutrition could be transferred to the next generation. We provide extensive data resources for future work in the context of potential intergenerational inheritance through the male germline.

**Supplementary Information:**

The online version contains supplementary material available at 10.1186/s12864-022-08348-4.

## Background

The influence of epigenetic information is not only limited to mitotic cell-to-cell inheritance but can be extended to meiotic intergenerational inheritance [1, 2]. In this view, alterations of phenotypic traits caused by external factors can be heritable without changes in DNA sequence and affect offspring phenotypes [3]. Nutrition is one of the major external factors that influence epigenomes [4, 5]. Intergenerational studies in zebrafish have revealed changes in DNA methylation in offspring when the parents are given different levels of fatty acids [6] and micronutrients [7]. DNA methylation is a reversible and heritable covalent modification of DNA found in the genomes of plants and animals [8–10]. Cytosine methylation at CpG sites is the most common form of DNA methylation in vertebrates [8] including Atlantic salmon (*Salmo salar*) [11].

In the present study, we sought to characterise and identify DNA methylation signatures in the male gonad of Atlantic salmon sampled from a feeding trial covering the whole life cycle (~ 54 weeks) [12]. Throughout the feeding trial, three groups of Atlantic salmon were fed plant-based diets with graded levels of micronutrient supplementation. The supplement was formulated based on a nutrition package (NP) that contained the recommended level of micronutrients for Atlantic salmon [12–14]. The NP contained 24 micronutrient components, including 13 vitamins, eight minerals, two crystalline amino acids, and cholesterol (Table 1). Previous studies that analysed fish from the same feeding trial reported that micronutrient supplementation enhanced growth performance [12] and epigenetically improved lipid metabolism in liver [11]. Nevertheless, the intergenerational impact of DNA methylation on offspring remained unanswered.

**Table 1:** Micronutrients contained in the NP (nutrient package) used in the feeding trial

Nutrient group	Micronutrients
Vitamin	Vitamin A, Vitamin D3, Vitamin E, Vitamin K3, Thiamine (B1), Thiamine (B1), Riboflavin (B2), B6, B12, Niacin (B3), Pantothenic Acid (B5), Folic Acid (B9), Biotin (B7), Vitamin C
Micro-mineral	Cobalt (Co), Iodine, Selenium, Iron, Manganese, Copper, Zinc
Macro-mineral	Calcium (Ca)
Amino acid	Taurine, Histidine
Cholesterol	Cholesterol

The DNA methylation status is globally cleared and reset during germ-cell specification as most methylated cytosine sites become unmethylated [13, 14]. During development, tissue-specific DNA methylation profiles are gradually re-established via comprehensive reprogramming processes [14, 15]. Altered epigenetic traits induced by environmental factors, including deficiency or excess of nutrients, can be transmitted to the offspring through the germline [16–18]. The knowledge about the intergenerational impact of DNA methylation in the male gonad is limited; nonetheless, a mouse study showed that hypo-methylated sperm DNA in aged fathers negatively affected neurodevelopment in its offspring [19]. Although both male and female gonads are primary reproductive organs to produce gametes, the yolk from the female gonad potentially conveys additional factors that contribute to intergenerational inheritance compared to milt from the male gonad. Hence, the male gonad can be an ideal organ to study a more direct effect of DNA methylation on intergenerational inheritance than the female gonad.

The present study aims to investigate DNA methylation signatures influenced by micronutrient supplementation in the male gonad of Atlantic salmon. We used reduced representation bisulfite sequencing (RRBS) [20] to measure differences in DNA methylation rates among three micronutrient-graded diet groups. To effectively annotate the sites and regions identified with differentially methylated status, we used data from multiple sources, such as gene expression data from RNA-seq, male liver data of the same feeding trial, and human orthologous data. Our results indicated that micronutrient supplementation affected the DNA methylation status in the male gonad. Specifically, the supplementation affected genes associated with vital pathways for the subsequent embryonic development, such as cell signalling, synaptic signalling, and brain development. Moreover, we identified an epigenetically affected region in the promoter of an evolutionarily conserved glutamate receptor gene, the *grin3a-like* gene, as a strong candidate for potential intergenerational DNA inheritance through the male germline. The present study represents important resources for future research in the context of intergenerational DNA inheritance in Atlantic salmon as well as among other vertebrates.

## Results

### Micronutrient supplementation improved the growth performance of Atlantic salmon

**Fig. 1:**
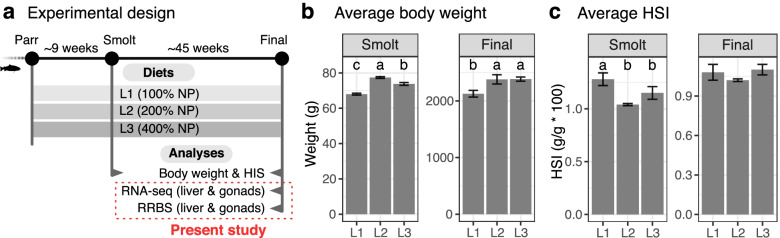
Experimental design and growth performance measurements. **a** Schematic plan of feeding trial and performed analyses of the present study. Bar plots show the average values with SEMs for **b** body weight and **c** Hepatosomatic Index (HSI) by diet. The calculation of both measurements is based on both male and female samples. Letters above the error bars indicate significant differences between groups (*p* < 0.05, one-way ANOVA) by the compact letter display of Tukey's HSD

To study potential intergenerational epigenetic inheritance through the male germline, we used male gonads obtained from a whole life-cycle feeding trial of Atlantic salmon with three feeding groups of parr, through smoltification, to the final harvest stage (Fig. 1a) [12]. The three feeding groups, L1, L2, and L3, were fed different levels of micronutrient supplementation throughout the experiment as L1, L2 and L3 contained 100, 200, and 400% of the recommended level of micronutrients, respectively (Fig. 1a, Additional file 1: Table S1&S2) [21–24].

As summarised in previous studies [11, 12], both medium (L2) and high (L3) doses of micronutrients contributed to improved growth performance when compared to the control diet (L1) by body weight, hepatosomatic index (HSI) [25], and Fulton's condition factor (K) [26]. At the smolt stage, L2 showed the best growth followed by L3 in terms of weight gain, whereas at harvest, both L2 and L3 showed significantly better growth (Fig. 1b, Additional file 1: Table S3). Accordingly, condition factor (K) indicated that L3 showed the best growth performance followed by L2 at harvest (Additional file 1: Table S3). HSI indicated that L1 likely retained more energy in liver instead of utilising it for growth than L2 and L3 at smolt (Fig. 1c, Additional file 1: Table S3).

For gene expression and DNA methylation analyses, we used male gonads and male liver collected at the final harvest stage (Fig. 1a) and then filtered them by gonadosomatic-index (GSI) to exclude the fish with overgrown gonads by GSI < 0.2%. We included liver samples for comparison purposes to elucidate gonad-specific patterns of transcriptional and epigenetic regulations as well as common patterns between them.

### Micronutrient supplementation affected overall gene expression profiles in male gonads but to a lesser degree than in liver

To study the influence of micronutrient supplementation on gene expression profiles, we performed differential expression analysis (DEA) by pair-wise comparisons with two data sets, defined as L2:L1 and L3:L1 (respectively L2 against L1, and L3 against L1; see 17), on uniquely mapped reads to the Atlantic salmon genome. A total of 35 RNA-seq samples, with 17 male gonad and 18 male liver samples, produced approximately 80% of the reads as uniquely mapped (Additional file 1: Table S4&S5). Each group contained an equal number of samples (*n* = 6) except for L2 gonads (*n* = 5).

**Fig. 2:**
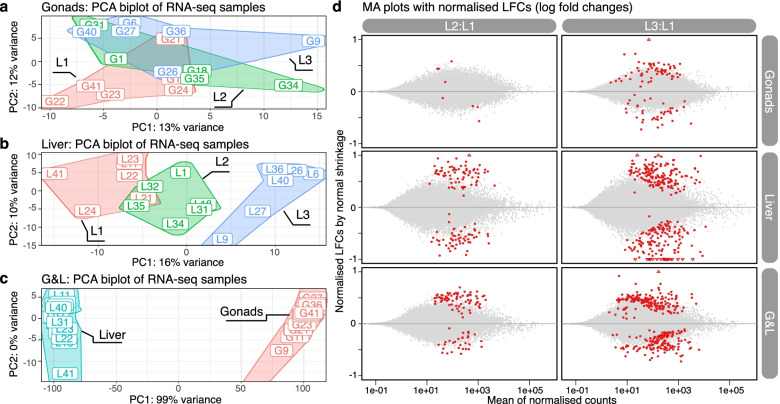
PCA biplots and MA plots showing gene expression patterns affected by diets. Three PCA biplots on the left show the first and second PCA components of top 500 high variance genes of RNA-seq counts with VST (variance stabilization transformation) for **a** gonads, **b** liver and **c** G&L (gonads and liver) datasets. The areas of three diet groups (L1: red, L2: green, L3: blue) and two tissue types (liver: turquoise, gonads: red) are outlined by convex hulls. d MA plots show the normalised read counts and log fold changes of all the mapped genes for gonads, liver and G&L datasets. Red dots indicate DEGs (adjusted *p*-value < 0.1), whereas grey dots indicate the genes with no significant differences

Prior to differential expression analysis, principal component analysis (PCA) showed no noticeable separations in gonads, regardless of using the top 500 high variance genes (Fig. 2a) or all the uniquely mapped genes (Additional file 1: Figure S1a). In contrast, PCA with liver samples produced clear separation by diet with L2 being intermediary (Fig. 2b, Additional file 1: Figure S1b). Furthermore, PCA with different tissues showed distinct clustering between gonads and liver on the first component of PCA biplots (Fig. 2c, Additional file 1: Figure S1c).

Differential expression analysis revealed that L3 had more differentially expressed genes (DEGs; adjusted *p*-value < 0.1) than L2 in gonads, with having only 6 DEGs for L2:L1 but 97 DEGs for L3:L1 (Fig. 2d, Table 2, Additional file 1: Figure S2). L3 also affected more genes than L2 in liver as well as in the dataset defined as G&L (see 17), which was used with a multi-factorial design to analyse a combinatorial effect of both tissues (gonads and liver) along with the diets (Fig. 2, Table 2). Moreover, most of the DEGs appeared to be tissue-specific as there were few overlapping DEGs between gonads and liver. Approximately 33.3% (2/6) and 7% (7/97) of the gonad DEGs were also identified as liver DEGs for L2:L1 and L3:L1, respectively (Additional file 1: Figure S3).

**Table 2:** The number of DEGs, enriched KEGG pathways, and enriched GO terms

	**n**^**a**^	**DEG**^**b**^		**KEGG**^**c**^	**GO**^**d**^			**GSEA**^**e**^	
		**Up**	**Down**		**BP**	**MF**	**CC**	**Up**	**Down**
Gonads									
L2:L1	5:6	3	3	0	0	0	0	7	2
L3:L1	6:6	60	37	0	0	0	0	2	7
Liver									
L2:L1	6:6	72	57	2	10	8	0	0	4
L2:L1	6:6	153	146	8	36	16	6	0	15
G&L									
L2:L1	12:12	75	30	0	3	0	0	5	4
L3:L1	11:12	196	150	3	10	0	0	2	22

^a^Number of samples represented as (# treatment group):(# control group). ^b^Number of DEGs defined by adjusted *p*-values < 0.1 and LFC > 0. ^c^Number of enriched KEGG pathways identified by ORA with adjusted *p*-values < 0.05 and the minimum gene count of 5. ^d^Number of enriched GO terms by ORA with adjusted *p*-values < 0.05 and the minimum gene count of 5. *BP* biological process, *MF* molecular function, and *CC* cellular component. ^e^Number of enriched KEGG pathways identified by GSEA with adjusted *p*-values < 0.05, minimum gene counts: 5 and abs(NES) > 2. Up & Down: the number of pathways enriched by up- and down-regulated genes, respectively

As expected, different nutrition levels influenced hepatic gene expression, as liver is an important organ with metabolic functions. Nonetheless, micronutrient supplementation also influenced gene expression in male gonads to some degree even though the gonads are organs that have limited metabolic activity compared to liver. Moreover, the results implied that high dosages of micronutrients (L3) affected more genes than medium dosages of micronutrients (L2) when compared to the control diet (L1) in both liver and gonads.

### The overall gene expression affected by micronutrient supplementation in male gonads showed association with cytokine receptor interaction, mismatch repair, and DNA replication

Even though there were too few DEGs for L2:L1 gonads (6 DEGs) for a robust functional annotation, L3:L1 gonads had enough DEGs (~ 100 DEGs) for over-representation analysis (ORA) on the KEGG (Kyoto encyclopedia of genes and genomes) [27] and the GO (gene ontology) [28] databases. Nevertheless, ORA identified neither enriched KEGG pathways nor GO terms even for L3:L1 gonad DEGs (Table 2). For liver and G&L DEGs, ORA identified enriched KEGG pathways and GO terms mainly related to lipid metabolism (Additional file 1: Tables S6-S9).

Gene set enrichment analysis (GSEA) is another functional annotation method that relies on the whole gene set instead of using only DEGs, and its NESs (normalized enrichment scores) indicate the trend of either up- or down-regulation of the identified pathways. GSEA on KEGG revealed in total 15, 16 and 29 enriched pathways for gonads, liver and G&L, respectively (Table 2, Additional file 1: Tables S10-S12). To control for potential false positive enrichment, we combined the identified pathways from L2:L1 and L3:L1 to make common pathways between them, which resulted in three enriched pathways from the original 15 pathways for gonads (Table 3). These functional annotation results were likely less robust in gonads than liver because none of the identified pathways by GSEA for gonads were supported by ORA results (Table 3). Nevertheless, the merged GSEA result between L2:L1 and L3:L1 implied that micronutrient supplementation potentially affected the expression of genes involved in three biological pathways in gonads: up-regulation for cytokine receptor interaction (sasa03030), and down-regulation for mismatch repair (sasa03430) and DNA replication (sasa04060), potentially in a tissue-specific manner.

**Table 3:** Enriched KEGG pathways identified both in L2:L1 and L3:L1 by GSEA

Tissue	Enriched pathways	KEGG ID	NES^a^	ORA^b^
Gonads	DNA replication	sasa03030	Down	-
	Mismatch repair	sasa03430	Down	-
	Cytokine-cytokine receptor interaction	sasa04060	Up	-
Liver	Steroid biosynthesis	sasa00100	Down	L2&L3
	Terpenoid backbone biosynthesis	sasa00900	Down	L2&L3
	Aminoacyl-tRNA biosynthesis	sasa00970	Down	-
G&L	Ribosome biogenesis in eukaryotes	sasa03008	Down	-
	Cell adhesion molecules	sasa04514	Up	-
	Cytokine-cytokine receptor interaction	sasa04060	Up	-
	Steroid biosynthesis	sasa00592	Down	L3
	Terpenoid backbone biosynthesis	sasa00900	Down	L3

^a^"Down" indicates down-regulation as identified by negative, *NES* (normalized enrichment score) values, whereas "Up" indicates up-regulation as positive *NES* values. ^b^Supported by ORA as "L2&L3" by both L2:L1 and L3:L1 and "L3" by only L3:L1. "-" indicates no ORA support

### Micronutrient supplementation altered DNA methylation patterns around transcription start sites in male gonads

In a similar way to differential expression analysis with RNA-seq samples, we performed differential methylation analysis by pair-wise comparisons with L2:L1 and L3:L1 datasets (see 17) on uniquely mapped reads to the Atlantic salmon genome to reveal the influence of micronutrient supplementation on DNA methylation profiles in male gonads. A total of 18 RRBS samples for male gonads and male liver produced approximately 46% of the reads as uniquely mapped (Additional file 1: Table S13). Each group contained an equal number of samples (*n* = 3).

**Fig. 3:**
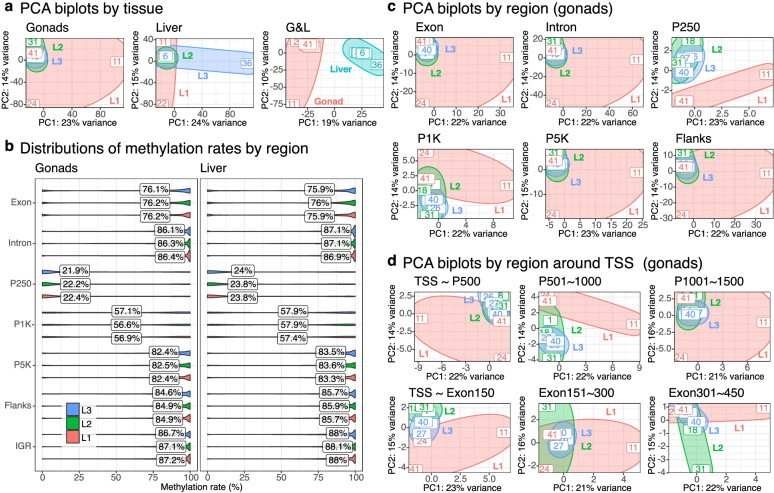
PCA biplots and violin plots showing DNA methylation patterns affected by diets. **a** Three PCA biplots show the first and second PCA components of DNA methylation rates for liver, gonads and G&L (gonads and liver) datasets. The areas of three diet groups (L1: red, L2: green, L3: blue) and two tissue types (liver: turquoise, gonads: red) are outlined with the ellipses estimated by the Khachiyan algorithm. **b** Violin plots show the regional distributions of methylation rates for gonads and liver datasets. Twelve PCA biplots on the right show the first and second PCA components of DNA methylation rates for **c** six different genomic regions (exon, intron, three promoter regions: P250, P1K and P5K, and flanking regions) and **d** six regions around TSS (three upstream promoter regions and three downstream exon regions from TSS). The areas of three diet groups (L1: red, L2: green, L3: blue) are outlined with the ellipses estimated by the Khachiyan algorithm

Prior to differential methylation analysis, we extensively performed clustering analysis to investigate the overall as well as regional DNA methylation patterns by diet. The result of PCA showed no distinct clusters by diet for both gonads and liver (Fig. 3a). Similar to gene expression, tissue-specific DNA methylation patterns appeared to be strong as indicated by the PCA biplot for the G&L dataset (Fig. 3a).

To elucidate regional DNA methylation patterns, we first separated the genome into three main regions: regulatory sequence (RS), gene body (GB), and intergenic region (IGR), which were further divided into four sub-regions: flanks (flanking regions both 10 K upstream and downstream around mRNAs) and promoter (P) within RS, and exon and intron within GB (see 17 for details). We also defined three sub-regions in promoters distinguished by upstream distances from transcription start sites (TSSs) as P250 (1 ~ 250 bp), P1K (251 ~ 1 K bp), and P5K (1001 ~ 5 K bp). Flanks were for covering potential enhancer regions, whereas P250 and P5K were for covering proximal and distal promoters, respectively.

Comparisons of the average methylation rates between genomic regions showed P250 had the lowest rates (~ 22%) followed by P1K (~ 57%) and exon (~ 76%) in gonads (Fig. 3b). There were no noticeable differences of methylation rates between diets even though the rates of L3 tended to be lower than those of L1 and L2 in gonads. The methylation rates in liver were similar to those in gonads even though the average rates of P250 in gonads (22.2%) were slightly lower than liver (23.7%; Fig. 3b).

Interestingly, the result of PCA with sub-regions showed distinct clustering for P250 and P1K in gonads, with having both L2 and L3 largely separated from L1 (Fig. 3c). The other sub-regions in gonads as well as all the sub-regions in liver, and G&L showed no such distinct patterns (Fig. 3c, Additional file 1: Figure S4a&S5a). Additional PCA that focused on both upstream (promoter) and downstream (exon) regions around TSS showed similar clustering between 1 K upstream and 150 bp downstream around TSS in gonads (Fig. 3d). Again, both liver and G&L showed no such distinct patterns around TSS (Additional file 1: Figure S4b&S5b). Therefore, clustering analysis suggested that micronutrient supplementation broadly affected DNA methylation profiles around TSS in male gonads.

### Differential methylation analysis revealed that most of the differentially methylated sites were tissue-specific

**Fig. 4:**
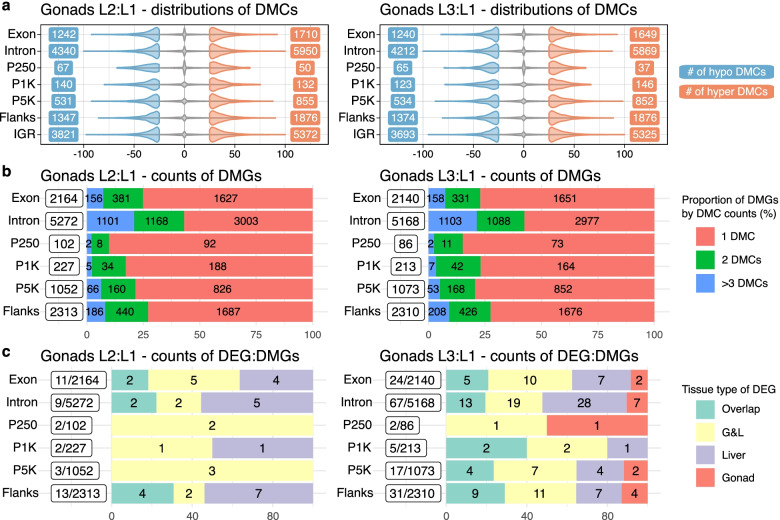
Violin plots and bar plots showing distributions and features of DMCs and DMGs. **a** Two violin plots on the top show the distribution of methylation rate differences of all the mapped CpG sites (grey background) as well as hypo-methylated (blue) and hyper-methylated (red) DMCs for L2:L1 and L3:L1 datasets. The label boxes display the numbers of corresponding DMCs. **b** Two stacked bar plots show the proportions of DMC counts (1 DMC, 2 DMCs and > 3DMCs) per DMG for L2:L1 and L3:L1 datasets. The numbers on the bars represent the numbers of corresponding DMGs. The label boxes next to the region names display the total number of DMGs per region. **c** Two stacked bar plots show the number of DEG:DMGs (DEGs that are also DMGs) for L2:L1 and L3:L1 datasets. The numbers on the bars represent the numbers of corresponding DEGs. The label boxes next to the region names display the ratio of DEGs and DMGs as (#DEG:DMGs)/(#DMGs). All the genes that belong to multiple datasets (for instance, liver and gonads) are categorized at "overlap"

Differential methylation analysis identified over 25 000 differentially methylated CpG sites (DMCs) defined by q-values < 0.01 and methylation rate differences > 25% for both L2:L1 and L3:L1 in male gonads (Table 4). The distributions of hypo- or hyper-methylated DMCs were balanced in all six sub-regions along with IGR (Fig. 4a). Although the counts and distributions of DMCs were similar between liver and gonads (Table 4, Additional file 1: Figure S6a), most of them were tissue-specific as only 5.3% and 5.9% of the gonad DMCs overlapped with the liver DMCs in L2:L1 and L3:L1, respectively (Additional file 1: Figure S7). The counts of DMCs in G&L were lower than both gonads and liver, potentially due to a multifactorial design with *n* = 12 rather than *n* = 6 (Table 4), which would contribute to smaller differences in methylation rates compared to gonads and liver (Additional file 1: Figure S8a). As expected, only a small fraction of the DMCs were G&L specific as over 97% of G&L DMCs overlapped with either gonads or liver (Additional file 1: Figure S7).

**Table 4:** The number of DMC, DMGs, enriched KEGG pathways and GO terms, and DEG:DMCs

	**n**^**a**^	**DMC**^**b**^	**DMG**^**c**^	**KEGG**^**d**^	**GO**^**e**^	**DEG:DMC**^**f**^		
						**Gonads**	**Liver**	**G&L**
Gonads								
L2:L1	3:3	27 433	9 774	20	107	**0**	21	20
L3:L1	3:3	26 995	9 647	12	95	**26**	59	69
Liver								
L2:L1	3:3	25 420	9 370	16	125	2	**20**	18
L3:L1	3:3	26 587	9 512	19	153	26	**72**	80
G&L								
L2:L1	6:6	3 786	1 981	1	13	0	2	**2**
L3:L1	6:6	3 716	1 977	2	25	7	16	**21**

^a^Number of samples represented as (# treatment group):(# control group). ^b^Number of DMCs defined by q-values < 0.01 and methylation rate differences > 25%. ^c^Number of DMGs defined as the genes with at least one DMC. ^d^Number of enriched KEGG pathways identified by ORA with adjusted *p*-values < 0.05 and the minimum gene counts of 20. ^e^Number of enriched GO terms by ORA with adjusted *p*-values < 0.001 and the minimum gene counts of 10. ^f^Number of DEGs that contain at least one DMC for gonads, liver and G&L (gonads and liver) datasets. Numbers in bold font are emphasised for those with matched datasets as gonads vs gonads, liver vs liver, and G&L vs G&L

We defined differentially methylated genes (DMGs) as protein-coding genes that contained at least one DMC, which led to the identification of over 9 500 DMGs in gonads (Table 4). Unlike DMC, DMG is just a term without any statistical evidence but simply to represent a gene that showed association with at least one DMC. In gonads, most of the DMGs were supported by single DMCs, ranging approximately from 60% in intron to 90% in P250 (Fig. 4b). Similarly, most of the DMGs were supported by only single DMCs for both liver and G&L (Additional file 1: Figure S6b&S8b). Although it was less tissue-specific than the DMCs (over 94%; Additional file 1: Figure S8), around 53% of the gonad DMGs remained tissue-specific (Additional file 1: Figure S9), suggesting micronutrient supplementation affected DNA methylation patterns differently in gonads and liver.

### Differentially methylated genes showed association with several cell signalling and metabolism pathways mainly by their gene bodies in both gonads and liver

Like functional annotation analysis with DEGs, we performed functional annotation analysis on DMGs. The number of identified DMGs was much higher than the corresponding DEGs. For instance, 97 DEGs vs. 9647 DMGs for gonad L3:L1, which could be a potential issue with functional annotation since the identified DMGs occupied a large part of the whole transcriptome. To investigate the regional contribution to enrichment, we labelled DMGs with 10 different regions as gene body (GB), intron, exon, promoter (P), P250, P1K, P5K, flanks, P + GB, and RS + GB (regulatory sequence + GB), depending on the locations of the corresponding DMCs. Also, one DMG could belong to multiple regional groups in case of having multiple DMCs.

Over-representation analysis (ORA) on KEGG identified 20 and 12 enriched KEGG pathways, whereas ORA on GO identified 107 and 95 enriched terms respectively for L2:L1 and L3:L1 in gonads (Table 4, Additional file 1: Tables S14-S17). Most enriched pathways and terms were associated with cell signalling and metabolism in gonads, and the enrichment results were similar to those in liver (Additional file 1: Tables S18-S21) and those of G&L to a lesser extent (Additional file 1: Tables S22-S24). Moreover, all the identified pathways and terms were enriched through the gene bodies except one pathway—the cellular senescence (sasa04218) pathway in P5K for gonad L2:L1 (Additional file 1: Table S14), which could be a good candidate for further studies targeting cell growth and death influenced by micronutrients in gonads.

Since a large part of the identified KEGG pathways overlapped between gonads and liver, we filtered the results to highlight the tissue specificity. We first eliminated the common enriched pathways identified in both gonads and liver, which resulted in a total of 10 pathways (Additional file 1: Table S25), and then further eliminated the common KEGG sub-classes, which resulted in a total of four unique enriched pathways (Table 5). This filtered result suggests that high dosages of micronutrients (L3) potentially influenced DNA methylation rates of the genes associated with the lysine degradation (sasa00310) and purine metabolism (sasa00230) pathways exclusively in male gonads.

**Table 5:** Enriched KEGG pathways with high tissue-specificity

	**Sub class**	**KEGG pathway**	**KEGG ID**	**Region (gene ratio)**
Gonads				
L3:L1	Amino acid metabolism	Lysine degradation	sasa00310	Exon (15/545)
	Nucleotide metabolism	Purine metabolism	sasa00230	Gene body (65/1898)
Liver				
L2:L1	Membrane transport	ABC transporters	sasa02010	RS + GB (25/2652), P + GB (22/2123), Gene body (20/1822)
L3:L1	Membrane transport	ABC transporters	sasa02010	RS + GB (29/2725), P + GB (27/2226), Gene body (26/1879), Intron (20/1486)
	Lipid metabolism	Fatty acid biosynthesis	sasa00061	P + GB (14/2226), Gene body (13/1879)

### Linking DEGs with DMCs identified two genes in which micronutrient supplementation simultaneously influenced both gene expression levels and DNA methylation signatures in male gonads

To study the epigenetic regulation of DNA methylation on gene expression affected by micronutrients, we merged DEGs with DMGs in all the possible combinations of tissue datasets (gonads, liver and G&L, see 17 for details) respectively for two diet datasets (L2:L1 and L3:L1), which resulted in 18 different DEG:DMCs (DEGs that had at least one DMC) counts (Table 4). These counts were statistically neither over nor under-represented, as indicated by the result of multiple linear regression showing that the counts of DEG:DMCs were mostly explained by the counts of DEGs and DMGs (#DEG:DMCs ~ #DEGs + #DMGs; adjusted R-squared: 0.83 and p-value: 7.1e-07; see 17). Moreover, there were no noticeable regional specific patterns of DEG:DMC counts (Fig. 4c, Additional file 1: Figure S6c&S8c).

For gonads, there were no DEG:DMCs for L2:L1 but 26 DEG:DMCs for L3:L1 (Table 4), with five DEG:DMCs located in promoters—one DEG:DMC in P250 and four DEG:DMCs in P5K (Additional file 1: Table S26). Among them, *guanylate cyclase 2G-like* (*gucy2g-like*) had two hypo-methylated DMCs (Additional file 1: Figure S10) with strongly up-regulated expression (LFC: 4.92), and *myelin basic protein-like* (*mbp-like*) had one hypo-methylated DMC with (Additional file 1: Figure S11) moderately up-regulated expression (LFC: 0.5) when fed the L3 diet. The human orthologue of *gucy2g-like* encodes a soluble guanylyl cyclase that has a potential role in water and electrolyte balance, and muscle contraction in response to calcium levels [29, 30], whereas the human orthologue of *mbp-like* encodes a protein that plays a key role in the process of myelination of nerves and interacts with the lipids in the myelin membrane [31]. Also, DNA methylation plays an essential role in the cellular reprogramming of non-myelinating cells in humans [32].

These potential epigenetic regulations of the two genes were likely gonad-specific, as both genes were neither DEGs nor DMGs identified through the promoters liver and G&L except that *mbp-like* was a DEG in L2:L1 G&L. The result of merging DEGs and DMCs suggests that *gucy2g-like* and *mbp-like* could be two good candidates epigenetically affected by micronutrient supplementation, as their gene expression may be regulated through DNA methylation on their promoter regions in male gonads.

### High and medium doses of micronutrients similarly affected gene expression and DNA methylation patterns

Our previous study that used fish from the same feeding trial as in the present study revealed strong transcriptomic and epigenetic regulations in a dose-dependent manner in liver [11]. It could be useful for effective filtering of false positives if there were any dose- or concentration-dependent regulations in male gonads. To investigate the dose-associated influence on both gene expression and DNA methylation, we combined L2:L1 and L3:L1 to create datasets that contained at least one DEG/DMC from either L2:L1 or L3:L1. In other words, datasets consisted of three types of pairs: one DEG/DMC in L2:L1, one DEG/DMC in L3:L1, and two DEGs/DMCs in both L2:L1 and L3:L1.

**Fig. 5:**
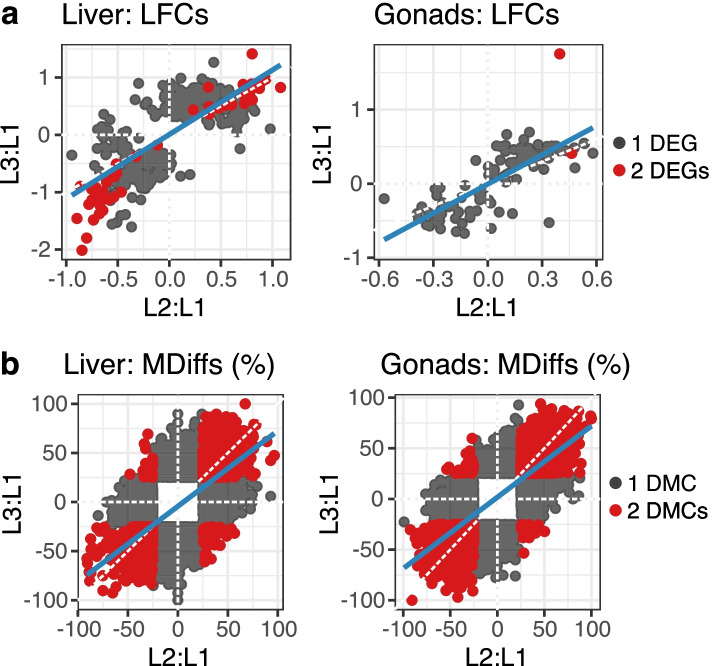
Scatter plots of LFCs and methylation rate differences between L2:L1 and L3:L1. **a** Scatter plots show LFCs of DEGs and corresponding genes between L2:L1 and L3:L1 for liver and gonads datasets. Gray dots indicate only one of them is DEG, whereas red dots indicate both of them are DEGs. The blue line represents a linear regression line estimated from all the dots. **b** Scatter plots show methylation differences (%) of DMCs and corresponding CpG sites between L2:L1 and L3:L1 for liver and gonads datasets. Gray dots indicate only one of them is DMC, whereas red dots indicate both of them are DMCs. The blue line represents a linear regression line estimated from all the dots

For gene expression, scatter plots of log fold changes (LFCs) between L2:L1 and L3:L1 showed strong positive correlations for gonads (*r* = 0.77, *p*-value < 2.2e-16) and liver (*r* = 0.74, *p*-value < 2.2e-16) along with linear regression lines with positive slopes (Fig. 5a), suggesting that L2 and L3 affected most of the genes in the same direction in terms of up- and down-regulation. DNA methylation differences (MDiffs) also showed strong positive correlations for gonads (*r* = 0.73, *p*-value < 2.2e-16) and liver (*r* = 0.59, *p*-value < 2.2e-16) again with positive regression lines (Fig. 5b), suggesting that L2 and L3 affected most of the CpG sites in the same direction in terms of hypo- and hyper-methylation.

To inspect potential gradual effects of L2 and L3, we split the dataset into positive and negative LFCs/MDiffs and compared the distributions between L2:L1 and L3:L1. Gene expression showed gradual effects in both liver and gonads (Additional file 1: Figure S12a), but it was most likely due to the number of DEGs as #(L3:L1 DEGs) > #(L2:L1 DEGs), where #(x) indicates the size of dataset x. Methylation differences showed a strong gradual effect towards L3 < L2 for hypo-methylation in liver but no noticeable effects in gonads (Additional file 1: Figure S12b).

Although there were no strong gradual effects on DNA methylation patterns in male gonads, high (L3) and medium (L2) doses of micronutrients still affected both gene expression and DNA methylation consistently in terms of down/up gene regulation and hyper/hypo methylation.

### Human orthologues of highly differentially methylated genes suggested associations with reproductive processes, synaptic signalling, and brain development

To make a list of candidate genes for potential intergenerational epigenetic inheritance in the male lineage, we merged L2:L1 and L3:L1 and then applied several filters to reduce the number of potential false positives. The first step was to merge common DMCs from L2:L1 (27 433 DMCs) and L3:L1 (26 995 DMCs) with the same direction in terms of hypo/hyper-methylation and exclude the sites in IGR (see 17), which produced 6082 common DMCs labelled with seven genomic regions—Exon, Exon150, Intron, P250, P1K, P5K, and Flanks. Exon150 contained DMCs located from TSS to 150 bp downstream, while Exon contained the rest of the DMCs in exons. We then defined "common DMGs" as the genes that had at least one common DMC and obtained 4336 common DMGs for gonads, along with 2869 and 426 common DMGs for liver and G&L, respectively (Additional file 1: Table S27). Subsequently, we sorted the list by the counts of DMCs per DMG and q-values and then selected the top three common DMGs from each region (Additional file 1: Tables S28-S30). Finally, we selected a total of nine genes from Exon150, P250, and P1K (Table 6, Additional file 1: Figures S13-S21) to review epigenetic regulations with DNA methylation in the literature using the corresponding human orthologues.

**Table 6:** Top three common DMGs in Exon150, P250, and P1K for gonads

**Region**	**Gene symbol**	**Gene name**	**Ortholog**^**a**^	**#DMCs**^**b**^	
				**Hypo**	**Hyper**
Exon150	*LOC106566321*	*potassium voltage-gated channel subfamily A member 2-like*	*KCNA2*	0	3
	*LOC106568430*	*putative uncharacterized protein DDB_G0286901*	*DDB_G0286901*	0	2
	*LOC106591755*	*nuclear pore complex protein Nup50-like*	*NUP50*	0	1
P250	*LOC106572512*	*glutamate receptor ionotropic, NMDA 3A-like*	*GRIN3A*	2	0
	*LOC106582681*	*dnaJ homolog subfamily C member 16-like*	*DNAJC16*	1	1
	*DEAD (Asp-Glu-Ala-Asp) box polypeptide 43*	*DEAD-box helicase 43*	*DDX43*	1	0
P1K	*LOC106611715*	*transmembrane protein 35-like*	*TMEM35A*	3	0
	*plcb4*	*phospholipase C beta 4*	*PLCB4*	0	3
	*LOC106578665*	*protein scribble homolog*	*SCRIB*	2	0

^a^Human orthologues in UniProt (www.uniprot.org) except for DDB_G0286901, which is an orthologue of slime mold (*Dictyostelium discoideum*). ^b^Number of the DMCs for hypo-methylated (hypo) and hyper-methylated (hyper)

In Exon150, the corresponding orthologues are *potassium voltage-gated channel subfamily a member 2* (*KCNA2*), which mediates transmembrane transport mainly in the brain and the central nervous system [33], *putative uncharacterized protein DDB_G0286901* (*DDB_G0286901*), which encodes a putative recombinant protein [34], and *nucleoporin 50* (*NUP50*), which encodes a component of the nuclear pore complex that plays a role in nuclear protein import [35]. Epigenetic regulations of *DDB_G0286901* and *NUP50* are unknown, but DNA methylation in the promoter of *KCNA2* is associated with attenuation of neuropathic pain in humans [36].

In P250, the corresponding orthologue of the most affected gene is *glutamate ionotropic receptor NMDA type subunit 3a* (*GRIN3A*), which encodes a subunit of *N-methyl-D-aspartate* (NMDA) receptor, which further belongs to the superfamily of glutamate-regulated ion channels [37]. The precise epigenetic regulation of *GRIN3A* is unknown, but DNA methylation in the promoter of *glutamate ionotropic receptor NMDA type subunit 2a* (*GRIN2A*), which is another subunit of NMDA, is strongly associated with major depressive disorder in humans [38]. Moreover, *glutamate ionotropic receptor NMDA type subunit 2b* (*GRIN2B*) is associated with anxiety-like behaviour in mice when juvenile mice were fed a methyl-donor-deficient diet [39]. The other two corresponding orthologues are *dnaj heat shock protein family (HSP40) member C16* (*DNAJC16*), which is a member of the heat-shock protein (HSP40) family [40], and *DEAD-box helicase 43* (*DDX43*), which encodes an ATP-dependent dual RNA–DNA helicase [41]. As for *DNAJC16*, a cohort study has reported that tea consumption in women epigenetically changes the gene expression of *DNAJC16* through DNA methylation of a single CpG site [42]. Aberrant DNA methylation status in the *DDX43* promoter is known to be associated with human cancers including acute myeloid leukemia [43].

In P1K, the corresponding orthologues are *transmembrane protein 35a* (*TMEM35A*), which encodes a soluble peptide that may modulate neurite outgrowth [44], *phospholipase C beta* (*PLCB4*), which encodes an enzyme that uses calcium as a cofactor to play an important role in extracellular signals [45], and *scribble planar cell polarity protein* (*SCRIB*), which encodes a membrane protein involved in cell migration and cell polarity [46]. While epigenetic regulation of *TMEM35A* and *SCRIB* are unknown, aberrant DNA methylation status in the *PLCB4* promoter affects hippocampal neurogenesis in mouse offspring upon maternal hexachlorophene (HCP) exposure [47].

Our differential expression analysis indicated that none of the nine common DMGs were DEGs in gonads. Nevertheless, *NUP50* and *DDX43* are highly expressed in testis, and *DNAJC16* is highly expressed in the oviduct epithelium in humans [48]. Hence, DNA methylation of these genes may have regulatory roles in the reproductive process. Moreover, *GRIN3A* is expressed in fetal brain in humans [48], suggesting DNA methylation of *glutamate receptor ionotropic, NMDA 3A-like* (*grin3a-like*) in Atlantic salmon potentially has a role in embryonic brain development.

### Analysis of a specific differentially methylated region revealed grin3a-like, ***hdac2***, and a zinc finger protein as prime candidates potentially involved in intergenerational epigenetic inheritance

As an alternative to DMCs, differentially methylation regions (DMRs) are widely used to detect genomic regions with different DNA methylation status instead of considering only single CpG sites. Nonetheless, most DMGs in the present study were supported by single DMCs (Fig. 4b), and DMCs were also sparsely located (for example, Additional file 1: Figure S10, S11, and S13-S21). Hence, we focused on loci with multiple DMCs that might have similar functionality to DMRs. Among the common DMGs, *grin3a-like* contained a locus with two hypo-methylated DMCs for gonads (Additional file 1: Figure S16) and G&L, and three hypo-methylated DMCs for liver within eight nucleotides of "CGCGCTCG" in its promoter (P250). Motif analysis with SalMotifDB [49] predicted three transcription factors that potentially bind this locus with multiple DMCs (Additional file 1: Table S31). Among them, *histone deacetylase 2* (*HDAC2*) and a zinc finger protein, *zinc finger protein 206* (*ZFP206*), had almost identically matched motifs to this locus (Additional file 1: Table S31), suggesting a potential interaction between *HDAC2* and *ZFP206*. *HDAC2* is a histone deacetylase that is responsible for the removal of acetyl groups from lysine residues of the core histones [50], whereas *ZFP206* is a DNA binding zinc finger protein that regulates a very small set of transcripts specific to embryonic stem (ES) cells [51] and controls pluripotency of ES cells [52].

Most entries in SalMotifDB are based on model organisms as *HDAC2* is from humans, and *ZFP206* is from both humans and mice. While *HDAC2* has a corresponding orthologue, *histone deacetylase 2* (*hdac2*; Gene ID: 101,448,008) in Atlantic salmon, *ZFP206* has no orthologues identified in Atlantic salmon as well as any other fish species. Since zinc finger proteins are one of the most abundant groups of proteins [53], there can be some zinc finger proteins that have a SCAN domain and a similar DNA binding motif to *ZFP206* in fish.

Transcripts of both *grin3a-like* and *hdac2* were expressed in male gonads, but neither of them were DEGs. No expression was detected for *grin3a-like* in liver, but *hdac2* showed weak expression in liver. Moreover, *hdac2* had one common DMC in its intron for gonads but no common DMCs for liver. Hence, micronutrient supplementation substantially affected DNA methylation status in the *grin3a-like* promoter but without alternating its gene expression in gonads.

### Combination of DMCs between the datasets of high and medium micronutrient doses identified over 4000 candidates for studying potential intergenerational epigenetic inheritance

In addition to the top three common DMCs from Exon150, P250, and P1K, all the other common DMGs could be strong candidates for studying potential epigenetic regulations. Since there were over 4000 common DMCs identified for gonads, it would be useful to provide an easy access data source for future work. Therefore, we summarised the data used in the present study into 13 tabular format files for gonads, liver and G&L available on figshare (https://doi.org/10.6084/m9.figshare.14177015.v1), and also created a website (https://nutrepi.github.io/wp1gonad) for fast and easy online data access specifically for gonads (see 17 and also Availability of data and materials). These datasets aims to provide various types of resources for studying potential intergenerational epigenetic inheritance through the male germline affected by different levels of micronutrients.

## Discussion

Our extensive analysis on DNA methylation in male gonads identified over 4000 CpG sites with differential methylation status induced by different levels of micronutrient supplementation. By using human orthologues, functional estimation of the top nine common DMGs with high methylation differences revealed that most of them were associated with neurodevelopment and synaptic signalling. In addition to these common DMGs in promoters, our functional annotation analysis revealed that the other general DMGs were associated with cell signalling in environmental information processing and cellular processing through the DMCs in their gene bodies. Hence, genes involved in signalling pathways are potentially susceptible to alterations of DNA methylation induced by environmental factors including different levels of micronutrients.

Establishing a robust method of analysing binding motif enrichment on multiple DMC sites could be useful to understanding intergenerational epigenetic regulation. A study about the effect of parental age on offspring in mice reported that genomic regions enriched in *RE1-silencing transcription factor* (*REST*) binding motifs were hypo-methylated in the sperm of aged fathers, and hypo-methylated sperm DNA negatively affected neurodevelopment in offspring [19]. Moreover, another mice study suggested that two zinc finger proteins, *zinc finger protein 217* (*Zfp217*) and *zinc finger protein 516* (*Zfp516*), controlled the concise epigenetic states on active embryonic stem cell (ESC) genes [54]. Interestingly, binding sites of *Zfp217* were largely overlapped with the target sites of a limited number of transcription factors, including *HDAC2* and *REST* [54]. Hence, DNA methylation of *grin3a-like* together with *hdac2* in the male gonad of Atlantic salmon could be one of the prime candidates for studying intergenerational epigenetic inheritance that affects early cell development stages in offspring.

Major limitations of the present study are (i) usage of the F0 gonads, (ii) the limited number of replicates in the DNA methylation analysis, and (iii) sparsely located DMCs. First, a feeding trial over generations to study intergenerational effects of micronutrient supplements is expensive and takes many years. In addition, most of the fish were too young to get fully matured even at the final sampling stage of the feeding trial, and therefore we focused on the male gonad to study the potential epigenetic inheritance affected by micronutrients. As the gonads are primary reproductive organs, the male gonad is the most suitable organ to study intergenerational epigenetic effects of the male linage especially when sperm cells are scarcely available. Second, the number of replicates used for the DNA methylation analysis was *n* = 3 for each group, and therefore, we utilized the liver samples, as verification, from one of our previous studies that used the fish from the same experiment with a larger number of replicates (*n* = 6 for two treatments and *n* = 9 for control) for the DNA methylation analysis [11]. Since the outcomes of this previous study were in line with those of the present study, we assumed that our DNA methylation analysis was robust enough to support our findings. Furthermore, we applied several strict filters to select genes with substantially affected CpG sites. Third, most of the identified DMGs in our study only contain single DMCs. Even though a single methylated site is enough to alter DNA binding affinity of transcription factors [55], false positives would be higher for the regions with single DMCs. In general, DMRs are more likely associated with biological functions than DMCs. As optimized methods of predicting DMRs are unclear for RRBS data with the salmon genome, benchmarking of DMR tools, such as, methylKit [56], DSS [57], and dmrseq [58], can be useful to find a method that effectively detects DMRs from sparsely located methylated CpG sites. Hence, further studies in both in vivo and in silico are needed to refine the candidates of intergenerational epigenetic modulators that respond to nutritional signals. To this end, we provide compressive data sets both as tabular format files and feature-rich data tables on a website for future work.

Our study also has a potential impact on reconsidering the optimised composition of broodstock diet in the aquaculture industry. Genetic and epigenetic regulations modulated by nutrients in broodstock of cultured fish have been less well studied compared to its offspring. Given that different micronutrient levels affect gene expression and DNA methylation profiles that may contribute to intergenerational inheritance in the male linage, micronutrients in male broodstock can be one of the key environmental factors that control the healthy growth and fish welfare of its offspring.

## Conclusions

The present study was aimed to unravel the impact of adding micronutrient supplementation in Atlantic salmon feed that potentially affected epigenetic regulations, specifically for DNA methylation. Although the feeding trial used in the present study was limited to one generation, we extensively analysed multiple types of data, such as different omics, different tissue types, human and mouse orthologues, and graded levels of nutrients, to examine overall and regional patterns of DNA methylation affected by micronutrients. The most heavily affected DNA methylation sites by micronutrient supplementation were mainly associated with cell signalling, neurodevelopment, and synaptic signalling. We also identified an epigenetically influenced genomic region in the promoter of the *grin3a-like* gene, where two transcription factors, *HDAC2* and *ZFP206*, were predicted to bind. The functional roles of *HDAC2* and *ZFP206* in humans and mice suggest that DNA methylation of the *grin3a-like* promoter may intergenerationally affect the early neurodevelopment of embryonic cells in offspring. To obtain a good understanding of the epigenetic inheritance triggered by nutrient signals in Atlantic salmon, we also provide a wide range of easy access datasets as important resources for future work.

## Methods

### Feeding trial

Atlantic salmon were obtained from SalmoBreed AS (Norway), and the whole feeding trial took place in the UK. During the freshwater phase, 500 salmon parr were kept in nine tanks at the Niall Bromage Freshwater Research Facility (Stirlingshire, UK; September 2014). After smoltification, the fish were transferred to the Mowi Marine Harvest Feed Trial Unit (Ardnish, Scotland; November 2014) and kept there for 12 months in sea pens. A nutrient package (NP) was used to supplement experimental diets to meet the required levels for Atlantic salmon as reported by the Advanced Research Initiatives for Nutrition & Aquaculture (ARRAINA) EU project [12, 22, 23]. L1, L2, and L3 were termed to represent three dietary groups prepared as 100%, 200%, and 400% of NP content, respectively. See the original study of the experiment [12] for more comprehensive descriptions of the feeding trial along with the summary of the trial. See also Additional file 1: Feeding trial, Experimental diets, and Micronutrient analysis of experimental diets in Supplementary Methods for further details.

### Sampling

At each sampling point in freshwater, 50 fish per tank were anaesthetised (50 mg/L Tricaine/MS222, PHARMAQ, UK, buffered with bicarbonate, 100 mg/L) and measured, while in seawater, all fish per tank were counted and individually measured; all fish were subjected to recovery in aerated water prior to letting them back to their original tanks. At the termination, six fish per tank were euthanised by lethal anaesthesia (> 200 mg/L Tricaine, PHARMAQ UK) for sequencing and molecular analyses. Among them, 17 gonad and 18 liver samples were used for gene expression analysis (Additional file 1: Table S4&S5), while nine gonad and nine liver samples were used for DNA methylation analysis (Additional file 1: Table S13).

All the fish in the feeding trial were euthanised by lethal anaesthesia (> 200 mg/L Tricaine, PHARMAQ UK) at the termination of the trial, and the fish that were not involved in this study were used in the ARRAINA EU project and reported elsewhere [11, 12, 59]. See the original study of the experiment for more details of sampling methods [12]. See also Additional file 1: Sampling and growth measurement in Supplementary Methods for further details.

### Extraction of DNA and RNA for sequencing

At the harvest stage of the trial, both gonads and liver were dissected followed by snap freezing in liquid nitrogen for RNA and DNA extraction. The same fish were used for both RNA and DNA samples, and ceramic beads were used to homogenise tissue samples. See Additional file 1: DNA and RNA extraction in Supplementary Methods for further details.

### Library preparation and sequencing for RNA-seq and RRBS

Both gonads and liver samples were sent to the DeepSeq sequencing facility at Nord University (Bodø, Norway) for RNA-sequencing (RNA-seq) where libraries were prepared using an NEBNext Ultra II Directional RNA Library Prep Kit for Illumina (New England Biolabs). The libraries were subsequently sequenced by the NextSeq500 machine (Illumina). See Additional file 1: RNA-seq library preparation and sequencing in Supplementary Methods for further details.

Both gonads and liver samples were sent to the CeMM Biomedical Sequencing Facility (Vienna, Austria) for reduced representation bisulfite sequencing (RRBS) where enzyme digestion by MspI and TaqI were performed followed by size selection and bisulfite conversion. RRBS was subsequently performed using the HiSeq 3000/4000 instruments (Illumina). See Additional file 1: RRBS library preparation and sequencing in Supplementary Methods for further details.

### Atlantic salmon genome and genome annotation

The genome data of the Atlantic salmon genome (ICSASG_v2) were obtained from the NCBI assembly site (https://www.ncbi.nlm.nih.gov/assembly/GCF_000233375.1).

Only the longest transcript was kept when a gene ID was associated with multiple isoforms with alternative splicing. The genome was separated into three main regions as regulatory sequence (RS), gene body (GB), and intergenic region (IGR). RS contained four sub-regions: P250, P1K, P5K, and flanks. P250 P1K, and P5K were promoter regions separated by the distances from TSS (transcription start site) as P250 (1 ~ 250 bp), P1K (251 ~ 1 K bp), and P5K (1001 ~ 5 K bp), whereas flanks were defined as 10 K up and downstream around mRNAs with excluding the regions defined as P250, P1K, and P5K. GB contained two sub-regions: exon and intron. IGR had no sub-regions. In case of overlapping, a site was exclusively assigned to one region or sub-region as the highest precedence given for exon followed by intron, P250, P1K, P5K, flanks, and IGR.

### Quality trimming, alignment, and quantification of RNA-seq reads

Raw reads were initially trimmed using Cutadapt [60] to remove low-quality reads (phred scores < Q30 or less than 20 bases) and adapters. STAR [61] was used with the default parameters to index the reference genome with RefSeq genes (ICSASG_v2) and align the quality trimmed reads to the indexed genome. The mapped reads were quantified using featureCounts [62] to estimate gene expression levels per gene based on the RefSeq genes (ICSASG_v2). Prior to differential expression analysis, principal component analysis (PCA) was performed using the factoextra package from CRAN (https://CRAN.R-project.org/package=factoextra).

### Differential gene expression analysis

Differential gene expression analysis was performed using the DESeq2 package [63] that produced log-fold changes with p-values adjusted by Benjamini-Hochberg. Differentially expressed genes (DEGs) were defined as the genes with adjusted *p*-values < 0.1.

The analysis was performed in a pair-wise manner using L1 as control for two datasets, termed L2:L1 and L3:L1 for each treatment group. In addition, samples of both gonads and liver were combined to generate the G&L dataset separately for L2:L1 and L3:L1. To perform a multifactorial analysis for the G&L dataset, tissue types (either gonads or liver) were added to the design matrix in addition to diet groups.

### Functional analysis of DEGs

The functional analysis of DEGs [64] was performed using the R package clusterProfiler [65, 66] based on the Kyoto Encyclopedia of Genes and Genomes (KEGG) database [27] for biological pathways and the Gene Ontology (GO) database [28] for gene ontology terms. Both KEGG and GO were used for over-representation analysis (ORA) [64], whereas only KEGG was used for gene set enrichment analysis (GSEA) [67]. Enriched KEGG pathways and GO terms were defined when p-values adjusted by Benjamini–Hochberg were less than 0.05 and a minimum gene count of five. Moreover, enriched pathways by GSEA were filtered by absolute values of normalized enrichment score (NES) > 2.

### Quality trimming, alignment and clustering of RRBS reads

FastQC (Babraham Institute; https://www.babraham.ac.uk) and MultiQC [68] were used to check the initial read quality, followed by trimming of adapters and low-quality reads performed by Trim Galore! (Babraham Institute) with the RRBS mode based on Cutadapt [60]. The reads longer than 50 bp were trimmed to make the maximum length of the reads 50 bp. Only the reads digested by two restriction enzymes, MspI and TaqI (around 97% of the total reads), were kept by an in-house Python script.

Bismark [69] with the default parameters for Bowtie 1 [70] was used to align the trimmed reads to the Atlantic salmon genome, and two Bismark functions, bismark_methylation_extractor and coverage2cytosine [69], were used to subsequent methylation calls at CpG sites. Prior to differential methylation analysis, principal component analysis (PCA) was performed using the factoextra package from CRAN (https://CRAN.R-project.org/package=factoextra).

### Differential methylation analysis

Differential methylation calling was completed with the methylKit R package [56] by calculating methylation differences with p-values for all the mapped CpG sites. Prior to differential methylation calling, reads with less than or equal to 10 and above the 99.9th percentile of coverage were discarded. The logistic regression method provided methylKit [56] was used to calculate methylation differences and p-values, and the SLIM method [71] also provided by methylKit was used to calculate q-values. Differentially methylated CpG sites (DMCs) were defined as the CpG sites with q-values < 0.01 and absolute methylation differences greater than or equal to 25%.

Similar to differential gene expression analysis, differential methylation analysis was performed in a pair-wise manner using L1 as control for two datasets, termed L2:L1 and L3:L1 for each treatment group. G&L datasets were formed in the same way as differential gene expression analysis separately for L2:L1 and L3:L1. Again, tissue types (either gonads or liver) were added to the design matrix in addition to diet groups to perform differential methylation analysis with G&L.

The plots of the genomic features with methylation differences and average methylation rates were generated by the Gvis R package [72].

### Functional enrichment of genes containing DMCs

Differentially methylated genes (DMGs) were defined when genes contained at least one DMC in the corresponding region. Like functional enrichment analysis with DEGs, over-representation analysis (ORA) [64] for DMGs was performed on both KEGG [27] and GO [28] by using the clusterProfiler R package [66]. Enriched KEGG pathways were defined by adjusted *p*-values < 0.05 and a minimum gene counts of 20, whereas enriched GO terms were defined by adjusted p-values < 0.001 and a minimum gene counts of 10.

### Linking DNA methylation with gene expression

DEGs and DMCs were merged to produce DEG:DMCs, which were defined as the DEGs that had at least one DMC. DEG:DMCs were produced for all nine possible pairs from the three datasets (gonads, liver, and G&L). A pair of the datasets comprised of one DEG dataset and one DMC dataset, for instance, (gonad DMGs, gonad DMCs), (gonad DMGs, liver DMCs), (gonad DMGs, G&L DMCs), and so on so forth. DMCs were used instead of DMGs to provide additional information for subsequent analyses.

To find statistically unexpected counts (too few or too large), a linear regression analysis with the formula of #DEG:DMCs ~ #DEGs + #DMGs was performed in R. Feature/variable-wise normalization on all the counts were performed by using the maximum counts before linear regression.

### Analysis with the common DMGs generated by merging L2:L1 and L3:L1

DMGs from L2:L1 and L3:L1 were merged to produce common DMGs identified in both datasets. Only matched directions of methylation differences (hyper and hyper, or hypo and hypo) were merged. DMGs in IGR were eliminated from the common DMGs. DMGs in exon were split into Exon150 and Exon. Exon150 was to cover the DMCs located near TSS (~ 150 bp downstream).

The website of UniProtKB [73] was used to estimate the orthologous genes for the common DMGs. The R version of SalMotifDB [49] with the default parameters was used to predict matching motifs and transcription factors.

### Tabular format files and website

As resources for further analyses, 13 tabular format files were generated and uploaded to Figshare – (1) DEGs for L2:L1, (2) DEGs for L3:L1, (3) DMCs and CpG sites for L2:L1, (4) DMCs and CpG sites for L3:L1, (5) DMCs by region for gonad L2:L1, (6) DMCs by region for gonad L3:L1, (7) DMGs for L2:L1, (8) DMGs for L3:L1, (9) DEG:DMCs for gonads (10) DEG:DMCs for liver, (11) DEG:DMCs for G&L, (12) common DMCs, (13) common DMGs. See Additional file 1: Tables S32-S44 for data descriptions.

In addition, a website with data for gonads was created by Jekyll (https://jekyllrb.com) and hosted on GitHub pages (https://nutrepi.github.io/wp1gonad).

### Bioinformatics pipelines

Both RNA-seq and RRBS pipelines were organised using Snakemake [74] to combine multiple bioinformatics tools, R and Python scripts for high-throughput sequence analysis. Moreover, R was used for basic statistical analysis and generating figures.

## Supplementary Information

**Additional file 1.**

## Data Availability

Both RNA-seq and RRBS datasets used by the present study are available from the Sequence Read Archive (SRA) on the National Center for Biotechnology Information (NCBI) website (https://www.ncbi.nlm.nih.gov/sra). The accession numbers of RNA-seq datasets are PRJNA680207 (https://www.ncbi.nlm.nih.gov/bioproject/PRJNA680207) for gonads and PRJNA632591 (https://www.ncbi.nlm.nih.gov/bioproject/PRJNA632591) for liver. The accession numbers of RRBS datasets are PRJNA680423 (https://www.ncbi.nlm.nih.gov/bioproject/PRJNA680423) for gonads and PRJNA628740 for liver (https://www.ncbi.nlm.nih.gov/bioproject/PRJNA628740). All tabular files generated by the present study are available on Figshare (http://dx.doi.org/10.6084/m9.figshare.14177015.v1). A website with rich-featured tables for gonads data is available on GitHub pages (https://nutrepi.github.io/wp1gonad).

## Abbreviations

NP: Nutrition package
RRBS: Reduced representation bisulfite sequencing
HSI: Hepatosomatic index
GSI: Gonadosomatic index
DEA: Differential expression analysis
PCA: Principal component analysis
DEG: Differentially expressed gene
G&L: Gonads and liver dataset
ORA: Over-representation analysis
KEGG: Kyoto encyclopedia of genes and genomes
GO: Gene ontology
GSEA: Gene set enrichment analysis
NES: Normalized enrichment score
RS: Regulatory sequence
GB: Gene body
IGR: Intergenic region
P: Promoter region
TSS: Transcription start site
P250: Proximal promoter between 1 and 250 bp from TSS
P1K: Promoter between 251 bp and 1 K bp from TSS
P5K: Distal promoters between 1001 and 5 K bp from TSS
DMC: Differentially methylated CpG site
DMG: Differentially methylated gene
HCP: Hexachlorophene
NMDA: N-methyl-D-aspartate
DMR: Differentially methylation region
HDAC2: Histone deacetylase 2
ES: Embryonic stem
REST: RE1-silencing transcription factor
ESC: Embryonic stem cell
