# Supplementary material for: Micronutrient supplementation affects DNA methylation in male gonads with potential intergenerational epigenetic inheritance involving the embryonic development through glutamate receptor-associated genes
Source: BMC Genomics. 2022 Feb 10;23:115. doi: 10.1186/s12864-022-08348-4 (PMC8832813; doi:10.1186/s12864-022-08348-4)
Supplement: Supplementary file 1 — Additional file 1. [file 12864_2022_8348_MOESM1_ESM.pdf]

Additional file 1: Supplementary information

Micronutrient supplementation affects DNA methylation signatures in male gonads with potential intergenerational epigenetic inheritance involving the embryonic development through glutamate receptor-associated genes

Takaya Saito, Paul Whatmore, John F. Taylor, Jorge M.O. Fernandes, Anne-Catrin Adam, Douglas R Tocher, Marit Espe, and Kaja H. Skjærven

Supplementary Methods

Feeding trial

The freshwater phase was carried out at the Niall Bromage Freshwater Research Facility (Stirlingshire, UK). Initially, 500 diploid salmon parr (initial mean weight, 38.2 ± 5.8g) were stocked (September 4th, 2014) into nine x 1.6m^3 circular fibreglass tanks (three tanks/diet). Fish were acclimatised to the experimental conditions for two weeks before being fed the experimental diets. Fish were fed continuously during the light period of the light dark cycle by automatic feeders (Arvotec T2000, Arvotec, Finland) controlled by a PC system. Specific feeding rates (SFR; % tank biomass per day) were adjusted automatically according to predicted growth and daily temperature. An out-of-season photoperiod (LL – 4 weeks LD14:10 – 4 weeks LL) and ambient water temperature (12 - 16˚C) was applied to produce S0+ smolts, with lighting provided by two 28W fluorescent daylight bulbs (4000°K, RS Components, UK) mounted centrally within the tank lid. Water was supplied by an upstream reservoir under flow-through conditions (10L/min). Oxygen levels were always higher than 8 mg/L. Uneaten feed recovery was not feasible during the freshwater phase.

Post-smolts were transferred (November 4th, 2014) to the Marine Harvest (Mowi) Feed Trial Unit, (Ardnish, Scotland) and on-grown for 12 months in 5 x 5 x 5 m sea pens under natural photoperiod and ambient water temperatures ranging from 6 to 16˚C. Triplicate groups of 250 post-smolts from respective tanks were stocked per pen and on-grown to a final size of ~3.0kg. Fish were fed continuously during daylight by automatic feeders (Arvotec T2000) controlled by a PC system. Specific feeding rates (SFR; % pen biomass per day) were adjusted weekly according to predicted growth and water temperature. Waste feed was collected per pen by means of air uplifts following meal delivery ensuring satiation and allowing calculation of total daily feed intake (Fi).

Experimental diets

Feeds were formulated to reflect standard practice in commercial salmon feeds in terms of protein, oil and energy contents. Thus, feeds were initially formulated to contain 48% protein and 20% lipid (~22 MJ), with protein content decreasing and lipid content increasing with increasing pellet size to

reach 36% protein and 34% lipid (~24 MJ) in the largest pellet size in seawater. The experimental feeds were a low FM (fish meal) / FO (fish oil) formulation (initially 15% and 8% in freshwater, decreasing to 5% and 3% respectively in seawater). Feeds were supplemented with a nutrient package (NP) at one of three inclusion levels to produce three dietary treatments: L1, 100% NP; L2, 200% NP; L3, 400% NP (Table S1), the assumption being that the 100% NP package should contain 100 % of assumed requirement based on the given requirement levels reported for Atlantic salmon at the time (NRC 2011) and modified according to earlier trials as part of the EU-funded ARRAINA project (Hamre et al. 2016; Hemre et al. 2016). Specifically, the NP contained 24 nutrients in total these being vitamins (A, D3, E, K3, C, thiamine, riboflavin, B6, B12, niacin, pantothenic acid, folic acid and biotin), minerals (Ca, Co, I, Se, Fe, Mn, Cu and Zn), crystalline amino acids (L-histidine and taurine) and cholesterol. Total and available phosphorus were fixed in all diets at 12.0 g/kg and 9.0 g/kg respectively, and magnesium at 1.5 g/kg, and were not part of the NP. Pellet size was adjusted according to fish weight (2mm, 3.5mm, 5mm, 7mm, 9mm). All non-oil ingredients were mixed, and pellets produced by extrusion to produce three base pellets that had oil added by vacuum coating. All feeds were produced at the BioMar Tech-Centre (Brande, Denmark).

Micronutrient analysis of experimental diets

Micronutrient analysis were performed by several different technologies and methods as described previously (Taylor et al. 2019). Vitamins were determined by microbiological methods and high-performance liquid chromatography (HPLC), whereas minerals were determined by inductively couple plasma mass spectrometry (ICP-MS).

Sampling and growth measurement

Fish were sampled at start and end of the freshwater phase, and then at approximately 250g, 500g, 1kg, and ~2.5kg in seawater prior to dietary pellet size/formulation changes. In freshwater at each sampling point, 50 fish per tank were anaesthetised (Tricaine/MS222, PHARMAQ, UK), individual weights (±0.1g) and fork lengths (±1.0mm) measured, while in seawater, all fish per pen were counted and individually measured.

For body weights and fork lengths, all fish per pen were individually measured. Following measurement, all fish were allowed to recover in aerated water before returning to their original experimental pens. Fulton's condition factor (K) was calculated using: $K = (W / L^3) * 100$ where W is body weight (g), and L is fork length (mm). Hepatosomatic index (HSI) was calculated as HSI (%) = (liver weight (g) / body weight (g)) * 100. Gonadosomatic-index (GSI) was calculated as GSI (%) = (gonad weight (g) / body weight (g)) * 100.

Following measurement, all fish were allowed to recover in aerated water before returning to their original experimental tanks/pens. Maturation at harvest was determined by assessment of external

appearance of secondary sexual characteristics and gonad development (n = 30 per pen). Fish were classified as sexually recruited based on a threshold value of GSI >0.20% or >1.0% for males and females, respectively (Kadri et al. 1997).

DNA and RNA extraction

At the end of the feeding trial, gonad and liver tissue were dissected for RNA and DNA extraction from 6 fish per diet at the same area of each individual, the ventral posterior lobe, and snap frozen in liquid nitrogen and stored at -80°C until further processing. DNA and RNA were extracted from same fish. For each feed group, liver samples were selected from the triplicate feeding tanks for RNA sequencing and RRBS. For both RNA and DNA extraction, tissue samples were homogenized using ceramic beads CK28 and a Precellys 24 homogenizer (Bertin Technologies).

RNA was extracted using the BioRobot EZ1 and EZ1 RNA Universal Tissue kit (Qiagen) and DNase treated with Ambion DNA-free DNA removal kit (Invitrogen, USA) according to the protocols. RNA quantity, which was 2460 ± 652 ng/ml on average, were assessed using NanoDrop ND-1000 Spectrophotometer (Nanodrop Technologies). RNA integrity (RIN), which was 9.55 ± 0.15 on average, were analysed using an Agilent 2100 Bioanalyser (RNA 6000 Nano LabChip kit, Agilent Technologies).

Homogenate from six single tissue samples from each feed group were resuspended in lysis buffer and DNA isolation was performed using the DNeasy Blood &Tissue Kit (Qiagen, Cat. No. #69506) according to the manufacturers protocol, except that after homogenization, tissue for DNA extraction were pre-treated with RNase A (provided by the Qiagen kit, 50ng/µL, 10 min at room temperature) immediately followed by proteinase K treatment (New England biolabs, #8102S 20µg/µL, 1.5 h at 55°C). DNA was eluted in Milli Q H_2O. Quantification of double stranded genomic DNA was done using the Qubit High Sensitivity Assay (Life Technologies #Q32854).

RNA-seq library preparation and sequencing

RNA-sequencing (RNA-seq) was performed by the DeepSeq sequencing facility at Nord University, Bodø, Norway. RNA-seq library preparation was completed using an NEBNext Ultra II Directional RNA Library Prep Kit for Illumina using the manufacturer protocol and workflow (New England Biolabs). Libraries were ligated with four primers using NEBNext Multiplex Oligos for Illumina Index Primer Sets 1, 2, 3 and 4. Samples were multiplexed into two pools of nine samples, with multiplexing barcodes ligated to each sample during the PCR amplification step, and further sequenced on the NextSeq500 machine (Illumina).

RRBS library preparation and sequencing

For RRBS, 100 ng of genomic DNA were digested for 6h at 65°C with 20 U TaqI (New England Biolabs) and 6h hours at 37°C with 20 U of MspI (New England Biolabs) in 30 µl of 1x NEB buffer

2. To retain even the smallest fragments and to minimize the loss of material, end preparation and adaptor ligation were performed in a single-tube setup. End fill-in and A-tailing were performed by addition of Klenow Fragment 3′ -> 5′ exo- (New England Biolabs) and dNTP mix (10 mM dATP, 1 mM dCTP, 1 mM dGTP). After ligation to methylated Illumina TruSeq LT v2 adaptors using T4 DNA Ligase rapid (Enzymatics), the libraries were size selected by performing a 0.75x clean-up with AMPure XP beads (Beckman Coulter).

The libraries were pooled based on qPCR data and subjected to bisulfite conversion using the EZ DNA Methylation Direct Kit (Zymo Research) with changes to the manufacturer's protocol: conversion reagent was used at 0.9x concentration, incubation performed for 20 cycles of 1 min at 95°C, 10 min at 60°C and the desulphonation time was extended to 30 min. These changes increase the number of CpG dinucleotides covered, by reducing double-strand break formation in larger library fragments. Bisulfite-converted libraries were enriched KAPA HiFi HS Uracil+ RM (Roche). The minimum number of enrichment cycles was estimated based on a qPCR experiment. After a 1x AMPure XP clean-up, library concentrations were quantified with the Qubit Fluorometric Quantitation system (Life Technologies) and the size distribution was assessed using the Bioanalyzer High Sensitivity DNA Kit (Agilent).

RRBS libraries were sequenced on Illumina HiSeq 3000/4000 instruments in 50-base-pair-single-end mode or 60-base-pair-single-end mode, and base calls provided by the Illumina Real-Time Analysis (RTA) software were subsequently converted into BAM format (Illumina2bam) before de-multiplexing (BamIndexDecoder) into individual, sample-specific BAM files via Illumina2bam tools (1.17.3 https://github.com/wtsi-npg/illumina2bam).

Supplementary figures

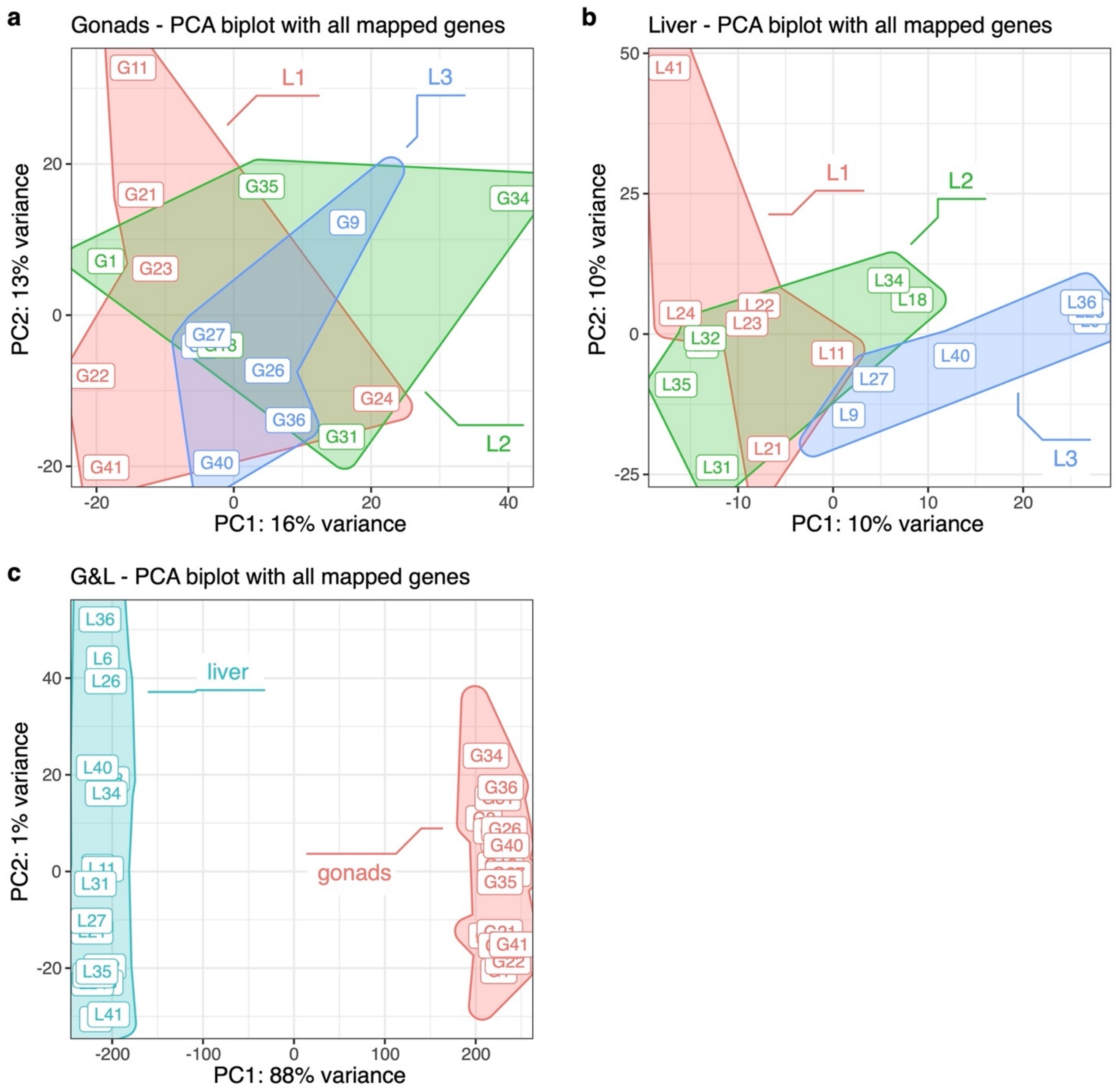

Figure S1. **PCA biplots showing gene expression patterns affected by diets.**
PCA biplots show the first and second PCA components of all the mapped genes of RNA-seq counts with VST (variance stabilization transformation) for **a** gonads, **b** liver and **c** G&L (gonads and liver) datasets. The area of three diet groups (L1: red, L2: green, L3: blue) and two tissue types (liver: blue, gonads: red) are outlined by convex hulls.

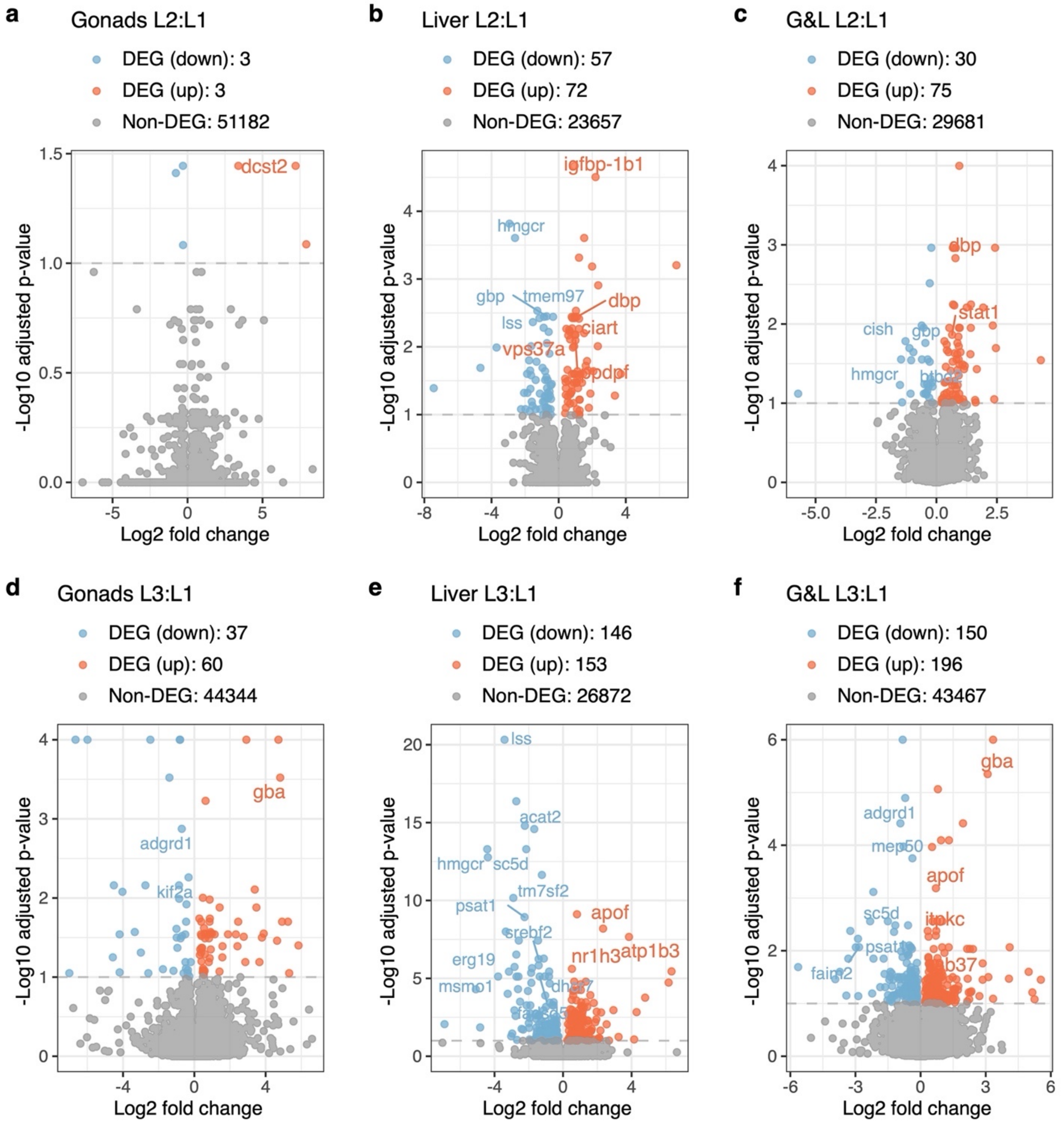

Figure S2. **Volcano plots showing LFCs and the corresponding adjusted p-values.**
Volcano plots show log2 fold changes vs -log10 adjusted p-values for **a** gonad L2:L1, **b** liver L2:L1, **c** G&L (gonads and liver) L2:L1, **d** gonad L3:L1, **e** liver L3:L1, and **f** G&L L3:L1. Gene symbols (non-LOC symbols) are shown for the genes with -log10 adjusted p-value lower than a dataset specific threshold (pval_label). Some plots have the maximum -log10 adjusted p-value (pval_max), and the p-values above the maximum are replaced with the maximum scores. Threshold values used for each plot are **a** pval_label: 1, **b** pval_label: 2, **c** pval_label: 1.5, **d** pval_label: 3, pval_max: 4, **e** pval_label: 5, and **f** pval_label: 2, pval_max: 6.

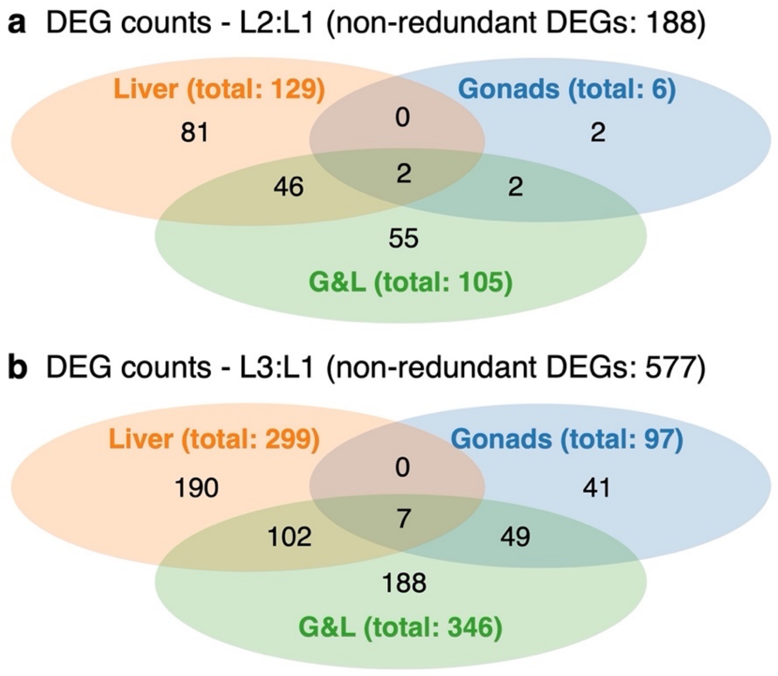

Figure S3. **Venn diagrams showing overlapped DEGs between liver and gonads.**
Ovals represent three DEG datasets of liver (orange), gonads (blue), and G&L (gonads and liver; green) with the counts of overlapped DEGs for **a** L2:L1 and **b** L3:L1 datasets. Non-redundant DEGs are a set of unique DEGs among all the three datasets - liver, gonads, and G&L.

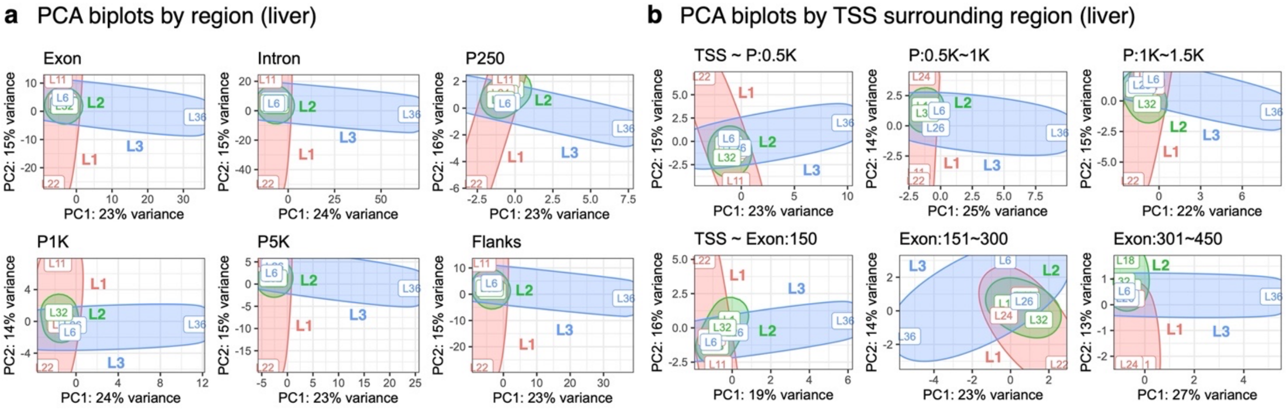

Figure S4. **PCA biplots showing DNA methylation patterns affected by diets in liver.**
PCA biplots show the first and second PCA components of DNA methylation rates for **a** six different genomic regions (exon, intron, three promoter regions: P250, P1K and P5K, and flanking regions) and **b** six regions around TSS (three upstream promoter regions and three downstream exon regions from TSS). The area of three diet groups (L1: red, L2: green, L3: blue) are outlined with the ellipses estimated by the Khachiyan algorithm.

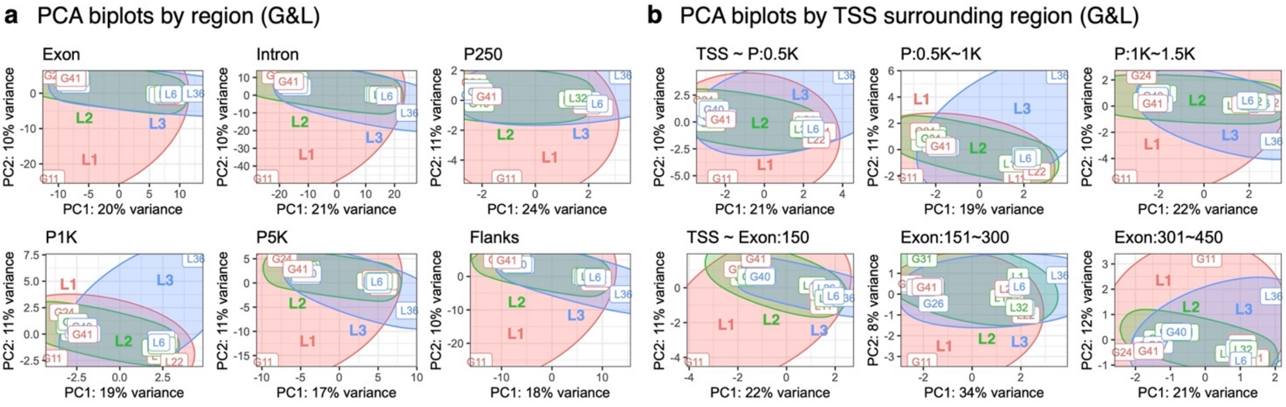

Figure S5. **PCA biplots showing DNA methylation patterns affected by diets in both gonads and liver (G&L).**

PCA biplots show the first and second PCA components of DNA methylation rates for **a** six different genomic regions (exon, intron, three promoter regions: P250, P1K and P5K, and flanking regions) and **b** six regions around TSS (three upstream promoter regions and three downstream exon regions from TSS). The area of three diet groups (L1: red, L2: green, L3: blue) are outlined with the ellipses estimated by the Khachiyan algorithm.

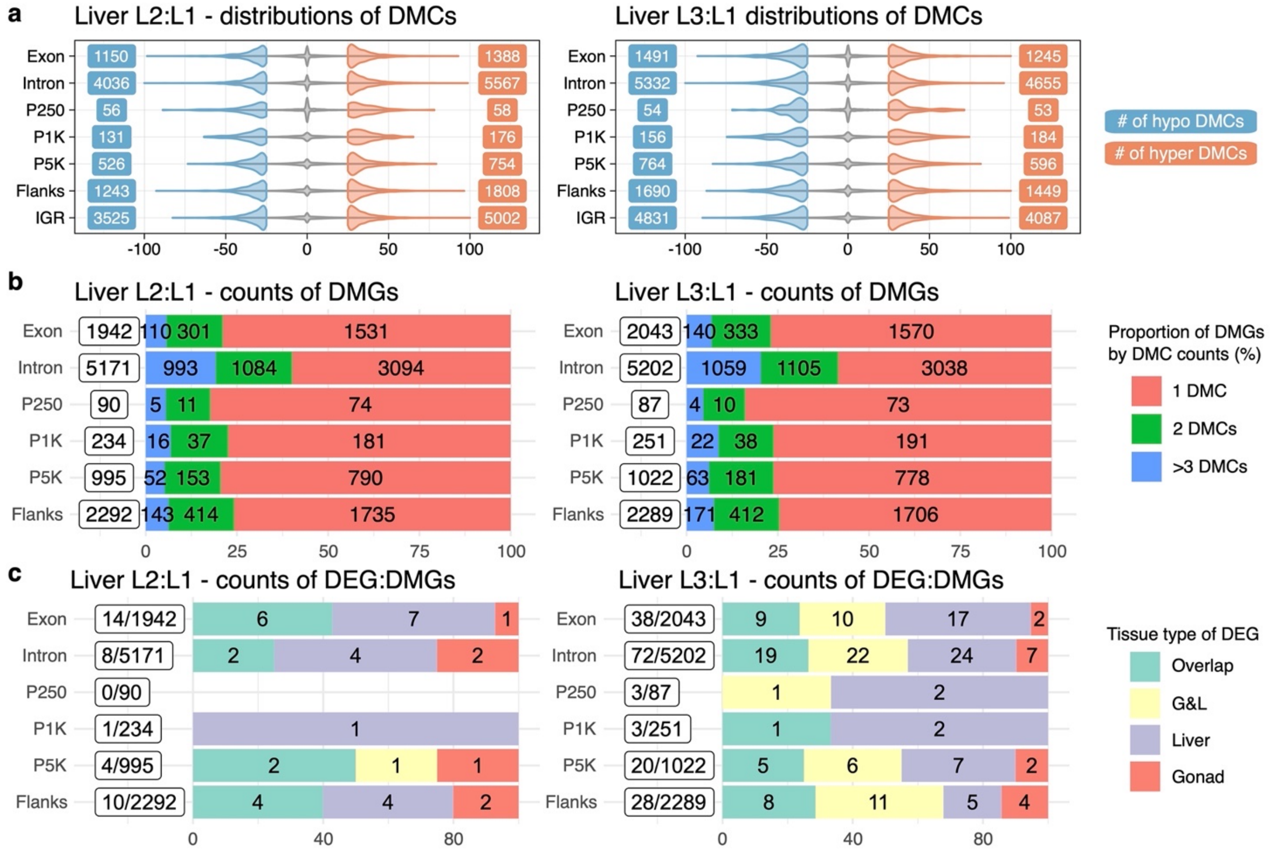

Figure S6. **Violin plots and bar plots showing distributions and features of DMCs and DMGs in liver.** **a** Two violin plots on the top show the distribution of methylation rate differences of all the mapped CpG sites (grey background) as well as hypo-methylated (blue) and hyper-methylated (red) DMCs for L2:L1 and L3:L1 datasets. The label boxes display the numbers of corresponding DMCs. **b** Two stacked bar plots show the proportions of DMC counts (1 DMC, 2 DMCs and >3DMCs) per DMG for L2:L1 and L3:L1 datasets. The numbers on the bars represent the numbers of corresponding DMGs. The label boxes next to the region names display the total number of DMGs per region. **c** Two stacked bar plots show the number of DEG:DMGs (DEGs that are also DMGs) for L2:L1 and L3:L1 datasets. The numbers on the bars represent the numbers of corresponding DEGs. The label boxes next to the region names display the ratio of DEGs and DMGs as(#DEG:DMGs)/(#DMGs). All the genes that belong to multiple datasets (for instance, liver and gonads) are categorized at "overlap".

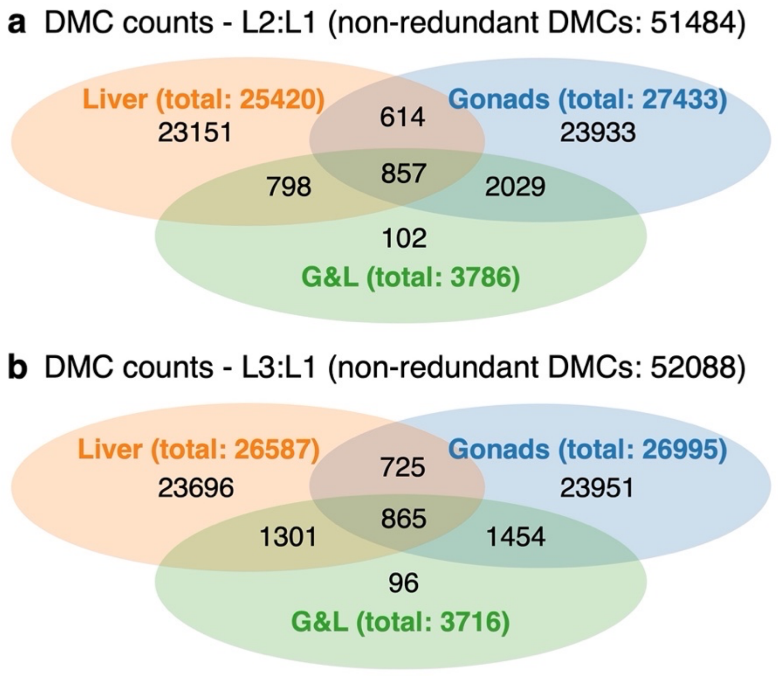

Figure S7. **Venn diagrams of overlapped DMCs between liver and gonads.**
Ovals represent three DMCs datasets of liver (orange), gonads (blue), and G&L (gonads and liver; green) with the counts of overlapped DMCs for **a** L2:L1 and **b** L3:L1 datasets. Non-redundant DMCs are a set of unique DMCs among all the three datasets - liver, gonads, and G&L.

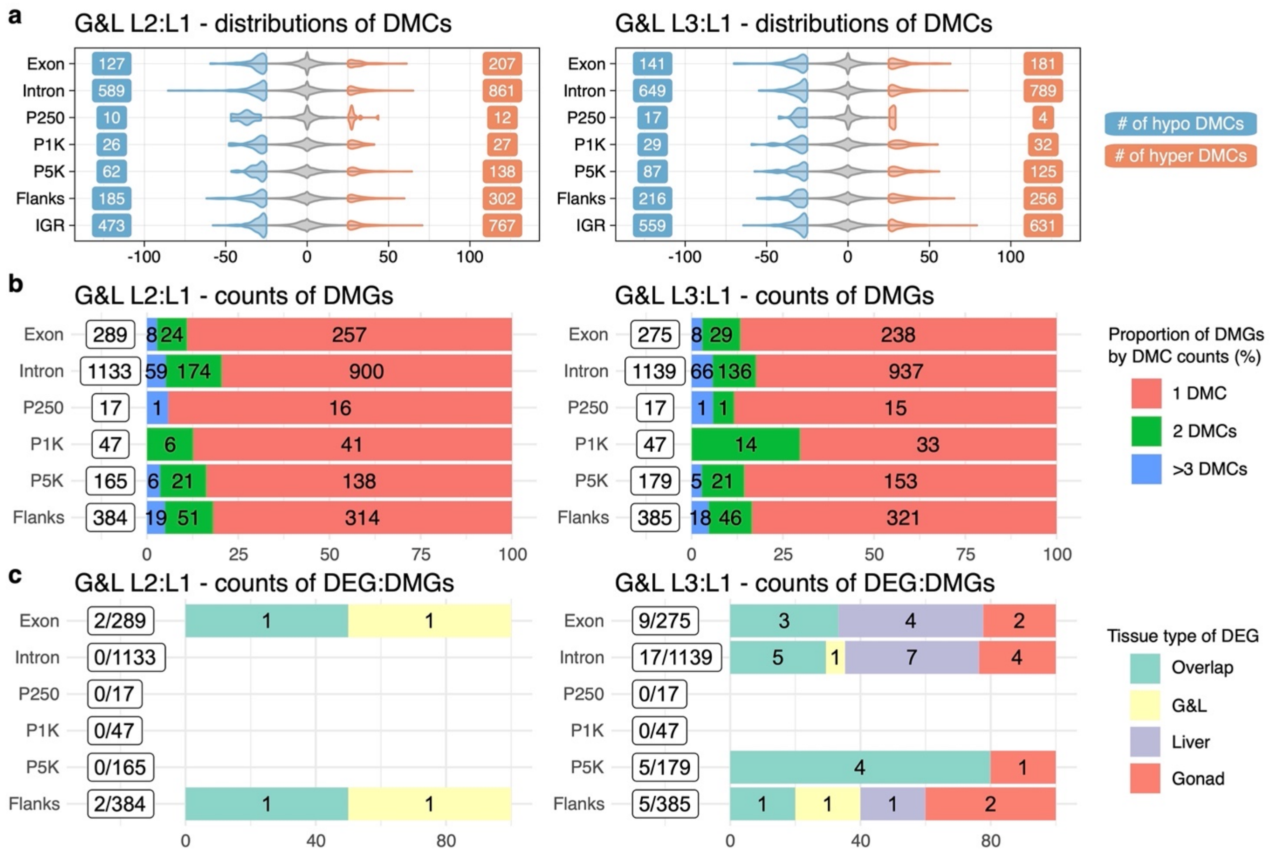

Figure S8. **Violin plots and bar plots showing distributions and features of DMCs and DMGs in both gonads and liver (G&L).**

a Two violin plots on the top show the distribution of methylation rate differences of all the mapped CpG sites (grey background) as well as hypo-methylated (blue) and hyper-methylated (red) DMCs for L2:L1 and L3:L1 datasets. The label boxes display the numbers of corresponding DMCs. **b** Two stacked bar plots show the proportions of DMC counts (1 DMC, 2 DMCs and >3DMCs) per DMG for L2:L1 and L3:L1 datasets. The numbers on the bars represent the numbers of corresponding DMGs. The label boxes next to the region names display the total number of DMGs per region. **c** Two stacked bar plots show the number of DEG:DMGs (DEGs that are also DMGs) for L2:L1 and L3:L1 datasets. The numbers on the bars represent the numbers of corresponding DEGs. The label boxes next to the region names display the ratio of DEGs and DMGs as(#DEG:DMGs)/(#DMGs). All the genes that belong to multiple datasets (for instance, liver and gonads) are categorized at "overlap".

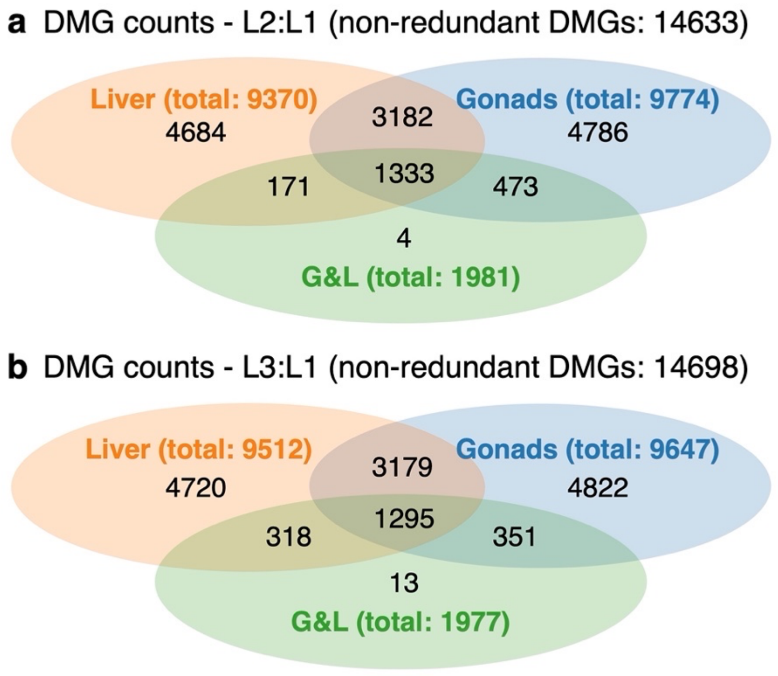

Figure S9. **Venn diagrams of overlapped DMGs between liver and gonads.**
Ovals represent three DMGs datasets of liver (orange), gonads (blue), and G&L (gonads and liver; green) with the counts of overlapped DMGs for **a** L2:L1 and **b** L3:L1 datasets. Non-redundant DMGs are a set of unique DMGs among all the three datasets - liver, gonads, and G&L.

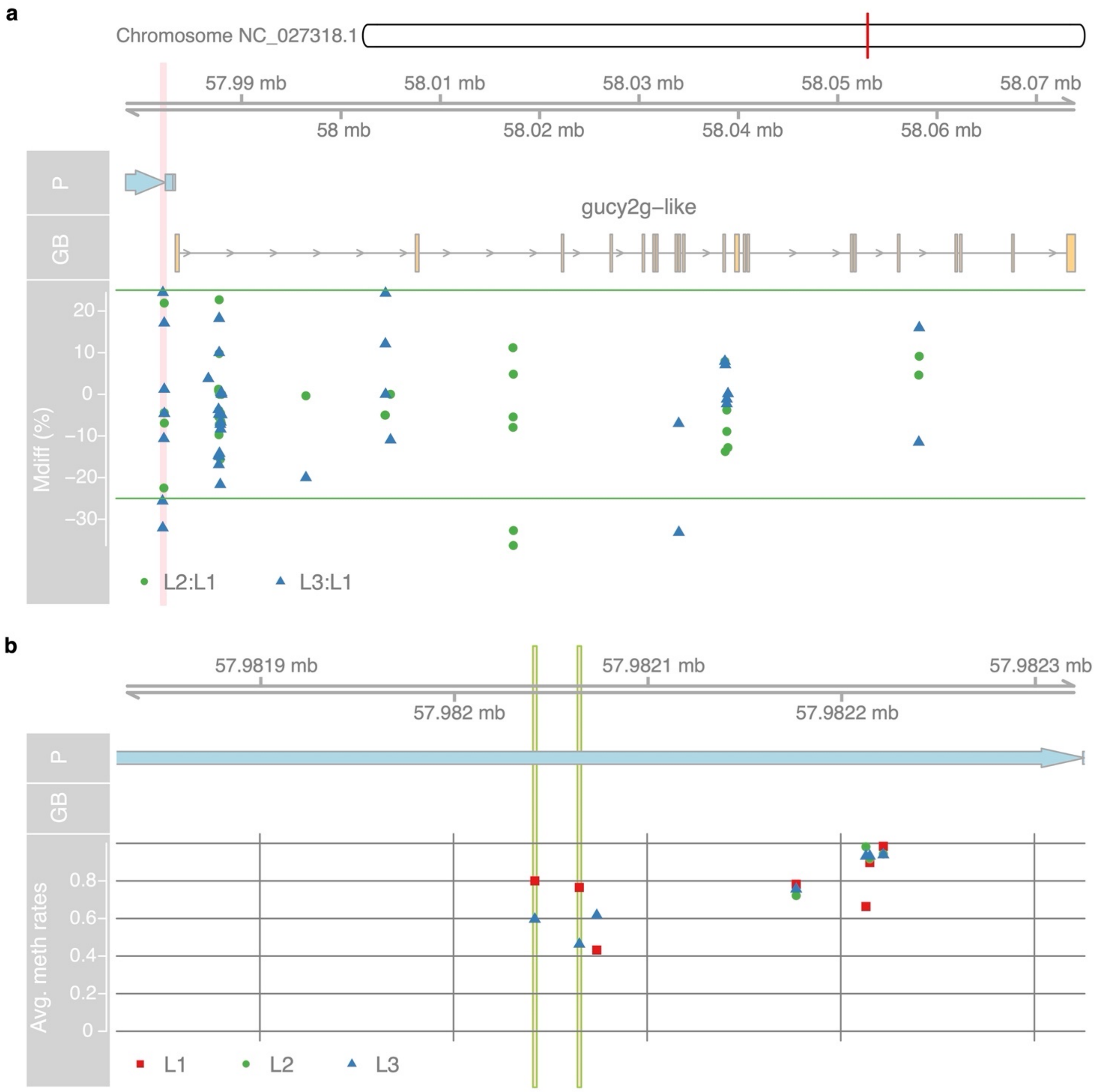

Figure S10. **Genomic feature view of *gucy2g-like* identified as a DEG:DMC in L3:L1**.
Visualization of genomic data of the *guanylate cyclase 2G-like* (*gucy2g-like*; *LOC106579347*) locus and its vicinity provides information of genomic features along with methylation differences and methylation rates. **a** The main track at the bottom shows differences of methylation rates for L2:L1 and L3:L1. Two green lines indicate thresholds of 25%. **b** An enlarged view shows the part of the region indicated as a pink rectangle in **a**. The main track at the bottom shows average methylation rates of L1, L2, and L3. Two DMCs of L3:L1 are highlighted with pink vertical bars.

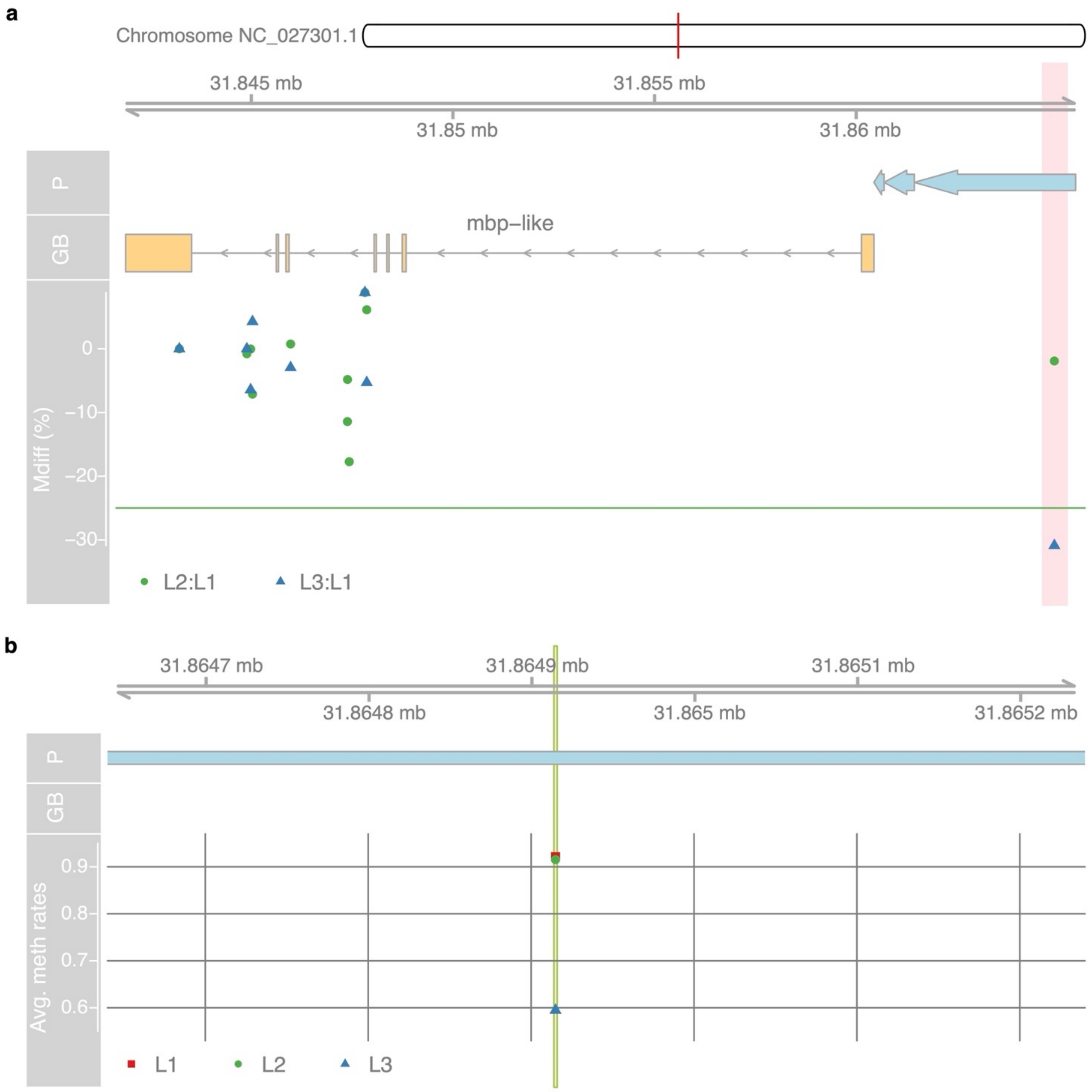

Figure S11. **Genomic feature view of *mbp-like* identified as a DEG:DMC in L3:L1**.
Visualization of genomic data of the *myelin basic protein-like, transcript variant X4* (*mbp-like*; *LOC106583543*) locus and its vicinity provides information of genomic features along with methylation differences and methylation rates. **a** The main track at the bottom shows differences of methylation rates for L2:L1 and L3:L1. The green line indicates a threshold of 25%. **b** An enlarged view shows the part of the region indicated as a pink rectangle in **a**. The main track at the bottom shows average methylation rates of L1, L2, and L3. One DMC of L3:L1 is highlighted with a pink vertical bar.

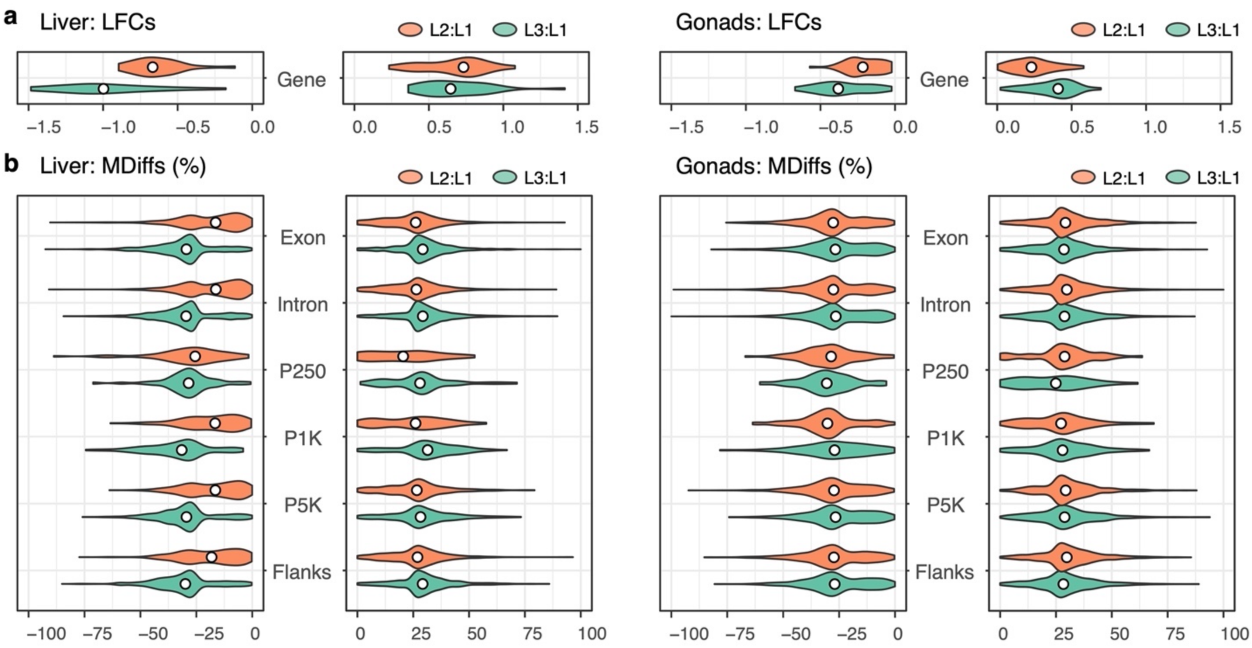

Figure S12. **Violin plots showing comparisons of LFCs and methylation rate differences between L2:L1 and L3:L1. a** Two violin plots show the distributions of the LFCs for L2:L1 (orange) and L3:L1 (green) for gonads and liver datasets. White dots indicate medians. The LFCs are filtered to contain only the genes identified as DEGs at least in one dataset. **b** Two violin plots show the distributions of the methylation rate differences for L2:L1 (orange) and L3:L1 (green) for gonads and liver datasets. White dots indicate medians. The methylation rate differences are filtered to contain only the CpG sites identified as DMCs at least in one dataset.

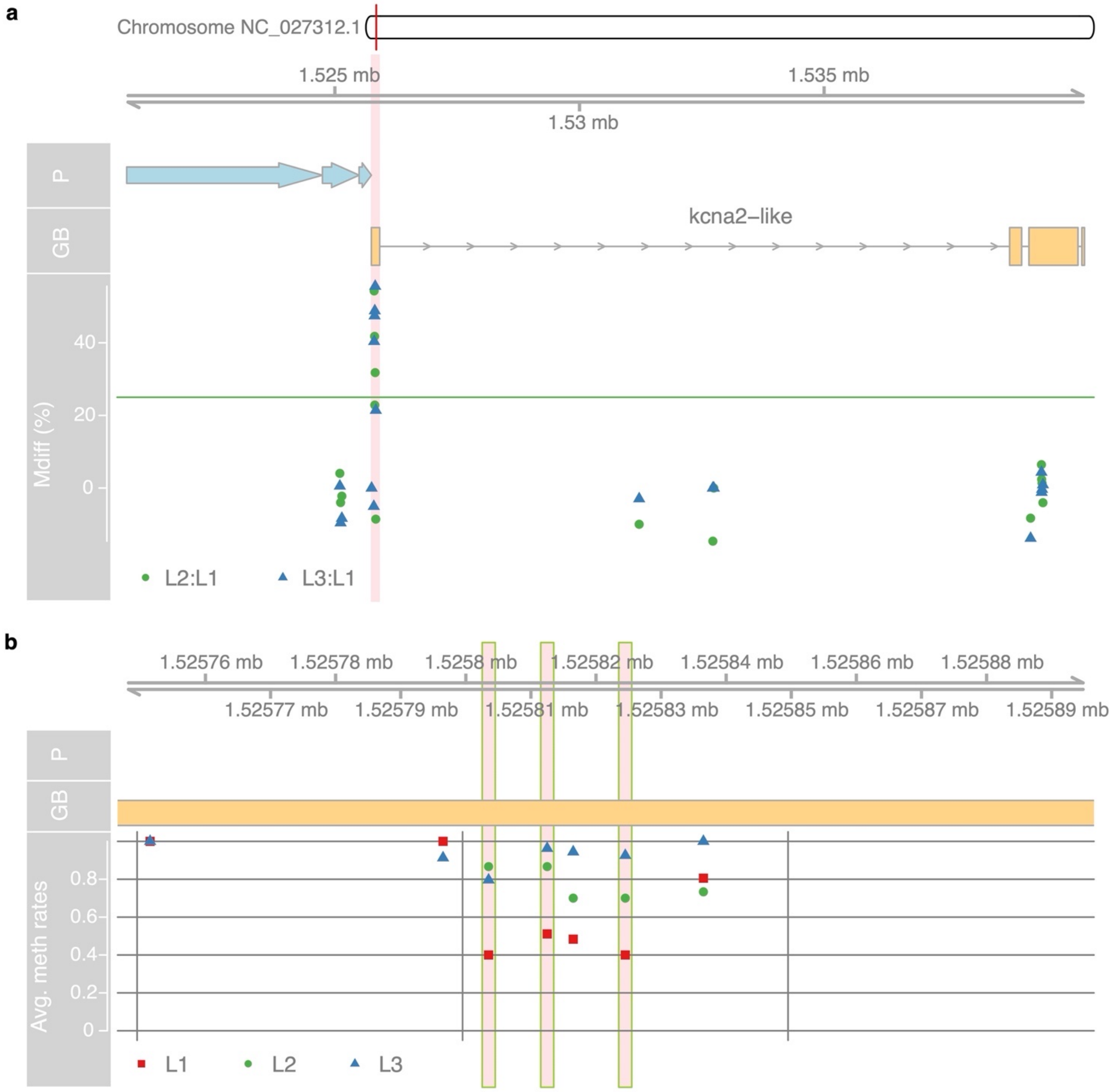

Figure S13. **Genomic feature view of *kcna2-like* with three common DMCs in Exon150**. Visualization of genomic data of the *potassium voltage-gated channel subfamily A member 2-like* (*kcna2-like*; *LOC106566321*) locus and its vicinity provides information of genomic features along with methylation differences and methylation rates. **a** The main track at the bottom shows differences of methylation rates for L2:L1 and L3:L1. The green line indicates a threshold of 25%. **b** An enlarged view shows the part of the region indicated as a pink rectangle in **a**. The main track at the bottom shows average methylation rates of L1, L2, and L3. Three common DMCs between L2:L1 and L3:L1 are highlighted with pink vertical bars.

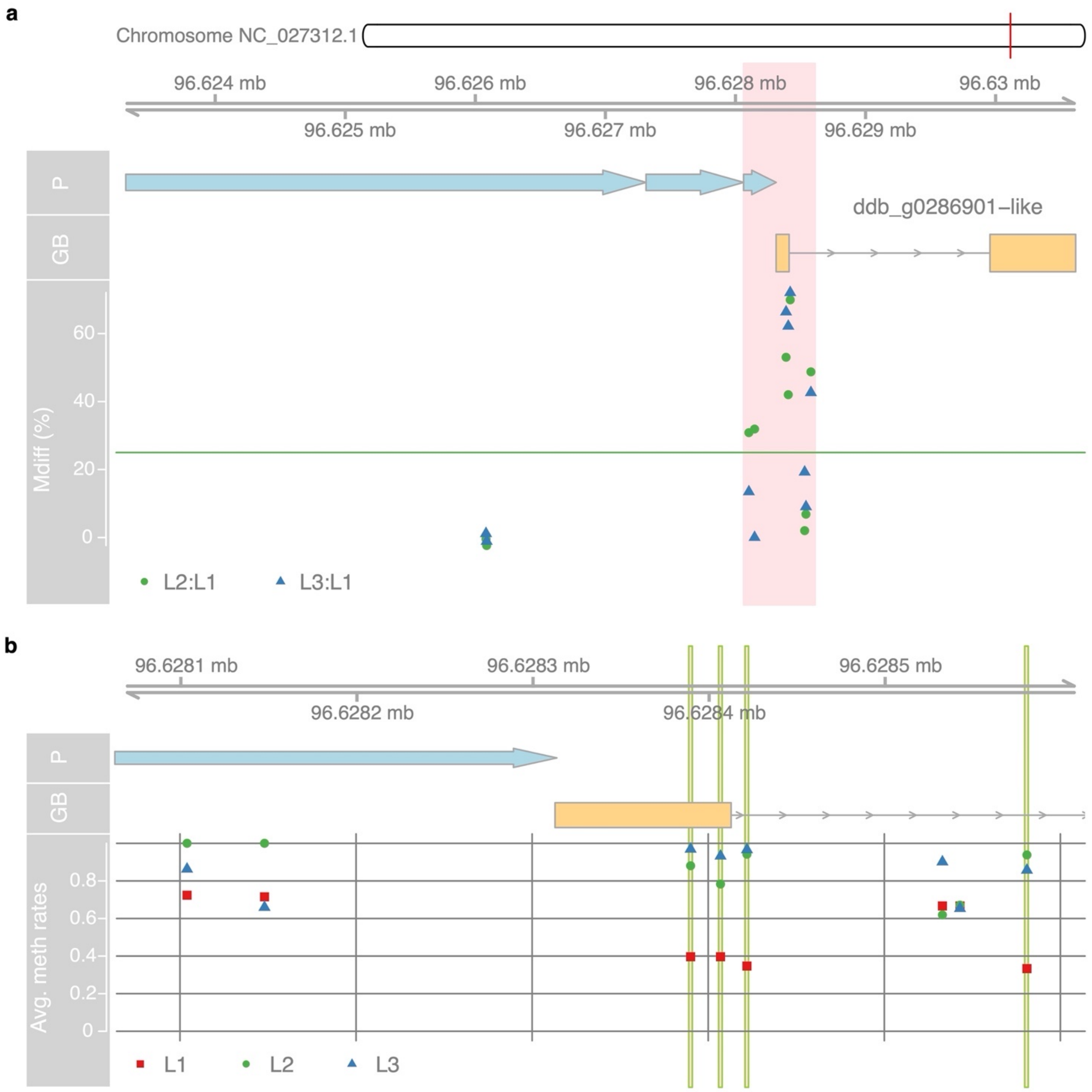

Figure S14. **Genomic feature view of *ddb_g0286901-like* with two common DMCs in Exon150**. Visualization of genomic data of the *putative uncharacterized protein DDB_G0286901* (*ddb_g0286901-like*; *LOC106568430*) locus and its vicinity provides information of genomic features along with methylation differences and methylation rates. **a** The main track at the bottom shows differences of methylation rates for L2:L1 and L3:L1. The green line indicates a threshold of 25%. **b** An enlarged view shows the part of the region indicated as a pink rectangle in **a**. The main track at the bottom shows average methylation rates of L1, L2, and L3. Four common DMCs between L2:L1 and L3:L1 are highlighted with pink vertical bars.

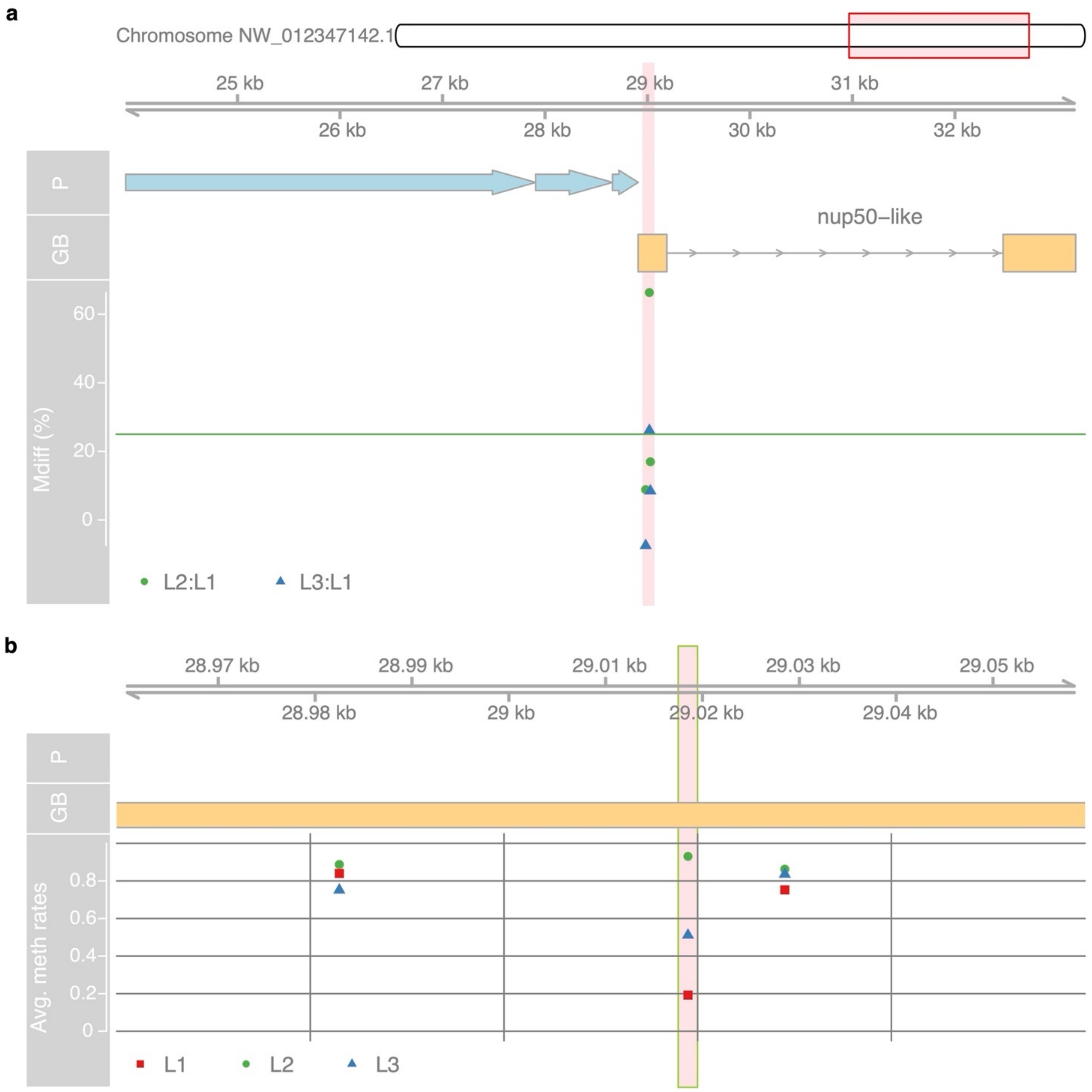

Figure S15. **Genomic feature view of *nup50-like* with one common DMC in Exon150**. Visualization of genomic data of the *nuclear pore complex protein Nup50-like* (*nup50-like*; *LOC106591755*) locus and its vicinity provides information of genomic features along with methylation differences and methylation rates. **a** The main track at the bottom shows differences of methylation rates for L2:L1 and L3:L1. The green line indicates a threshold of 25%. **b** An enlarged view shows the part of the region indicated as a pink rectangle in **a**. The main track at the bottom shows average methylation rates of L1, L2, and L3. One common DMC between L2:L1 and L3:L1 is highlighted with a pink vertical bar.

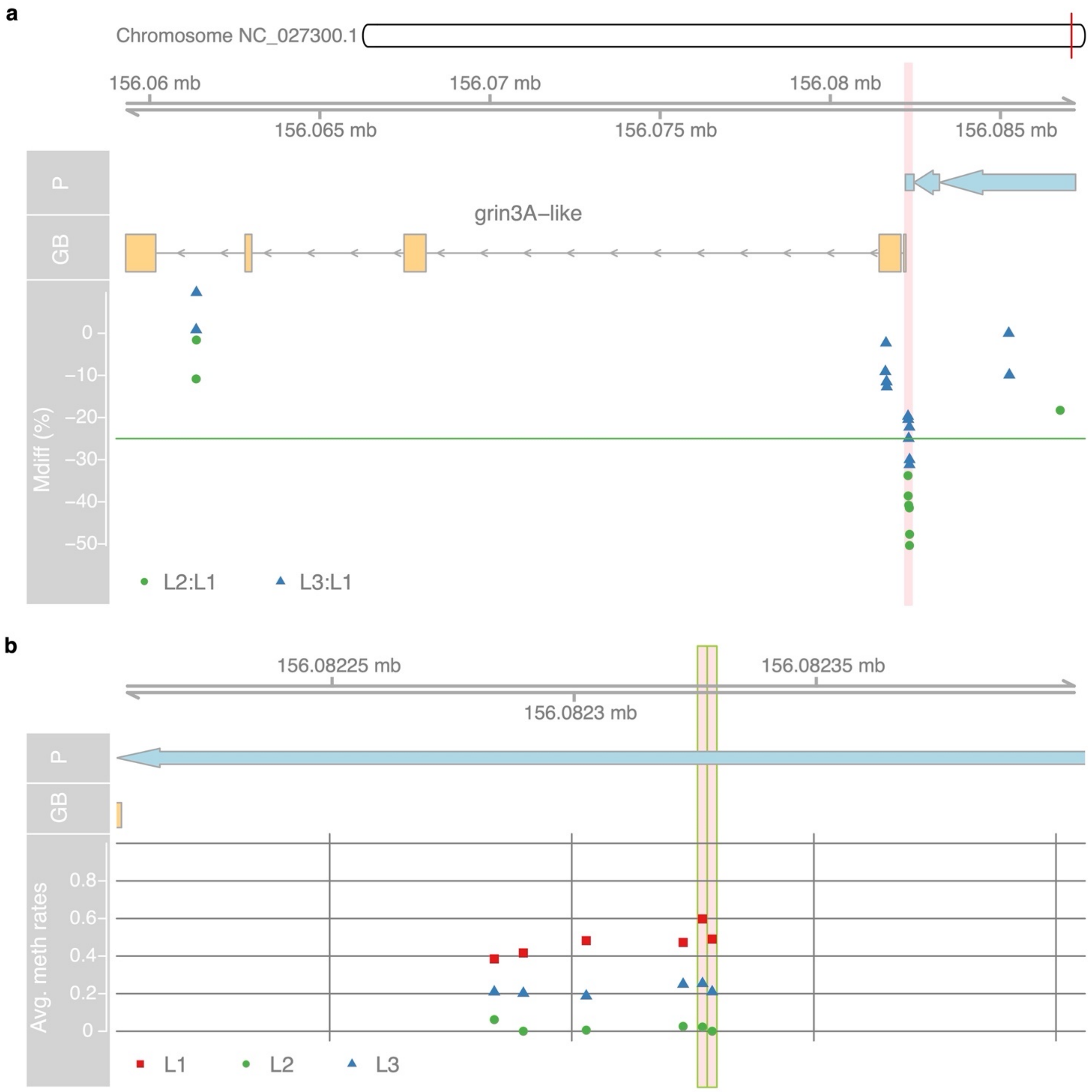

Figure S16. **Genomic feature view of *grin3a-like* with two common DMCs in P250**. Visualization of genomic data of the *glutamate receptor ionotropic, NMDA 3A-like* (*grin3a-like*; *LOC106572512*) locus and its vicinity provides information of genomic features along with methylation differences and methylation rates. **a** The main track at the bottom shows differences of methylation rates for L2:L1 and L3:L1. The green line indicates a threshold of 25%. **b** An enlarged view shows the part of the region indicated as a pink rectangle in **a**. The main track at the bottom shows average methylation rates of L1, L2, and L3. Two common DMCs between L2:L1 and L3:L1 are highlighted with pink vertical bars.

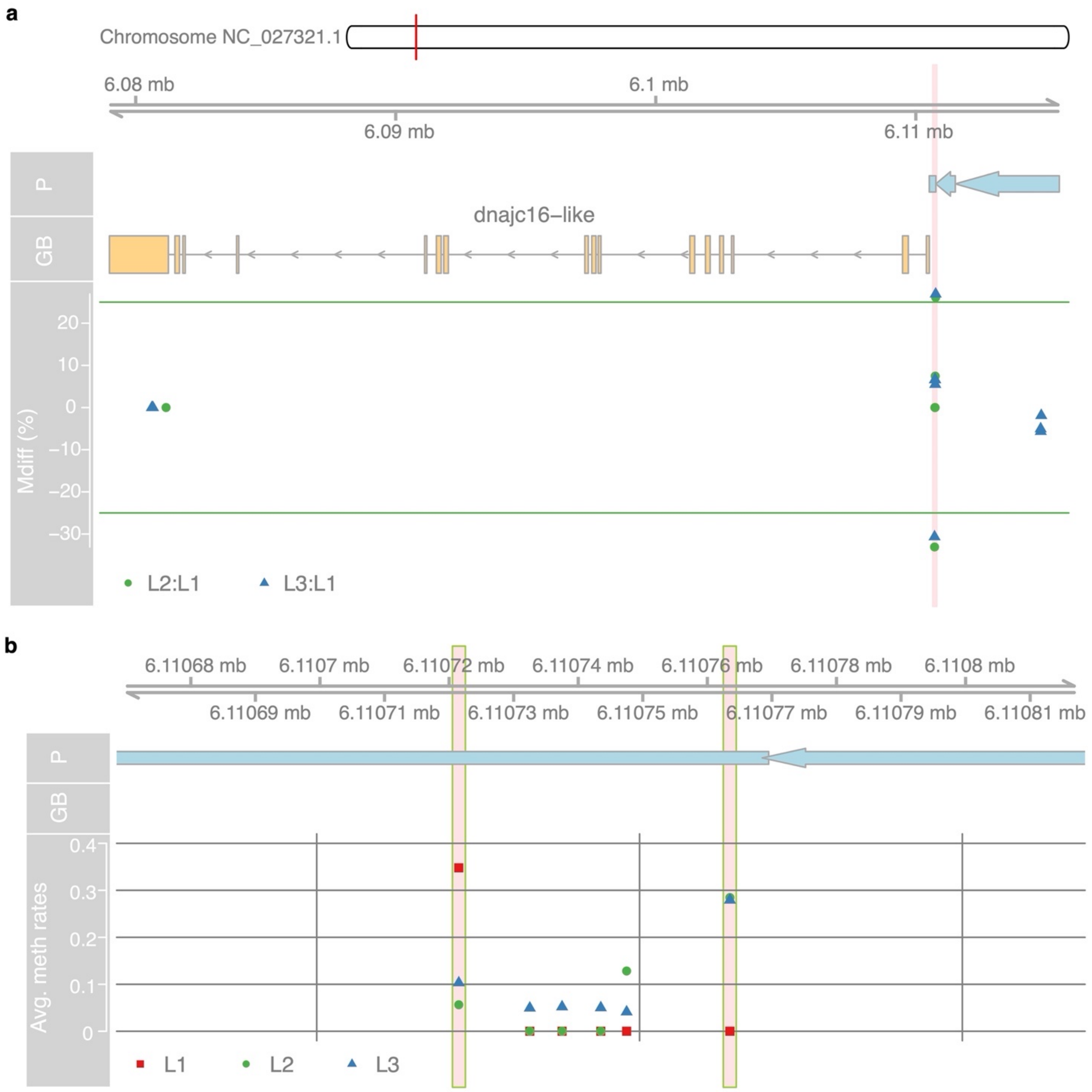

Figure S17. **Genomic feature view of *dnajc16-like* with two common DMCs in P250**. Visualization of genomic data of the *dnaJ homolog subfamily C member 16-like* (*dnajc16-like*; *LOC106582681*) locus and its vicinity provides information of genomic features along with methylation differences and methylation rates. **a** The main track at the bottom shows differences of methylation rates for L2:L1 and L3:L1. Two green lines indicate thresholds of 25%. **b** An enlarged view shows the part of the region indicated as a pink rectangle in **a**. The main track at the bottom shows average methylation rates of L1, L2, and L3. Two common DMCs between L2:L1 and L3:L1 are highlighted with pink vertical bars.

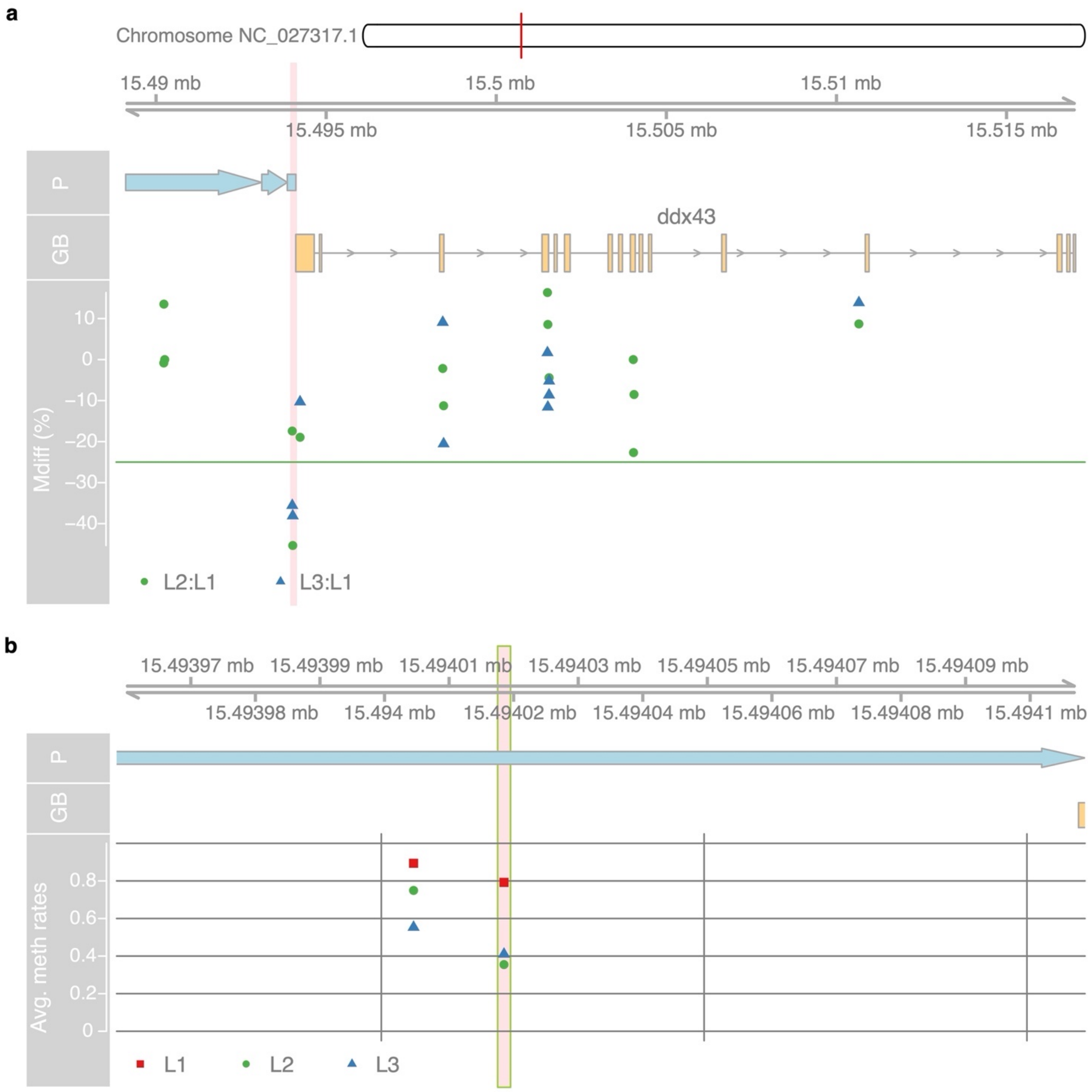

Figure S18. **Genomic feature view of *ddx43* with one common DMC in P250**.
Visualization of genomic data of the *DEAD (Asp-Glu-Ala-Asp) box polypeptide 43* (*ddx43*) locus and its vicinity provides information of genomic features along with methylation differences and methylation rates. **a** The main track at the bottom shows differences of methylation rates for L2:L1 and L3:L1. The green line indicates a threshold of 25%. **b** An enlarged view shows the part of the region indicated as a pink rectangle in **a**. The main track at the bottom shows average methylation rates of L1, L2, and L3. One common DMC between L2:L1 and L3:L1 is highlighted with a pink vertical bar.

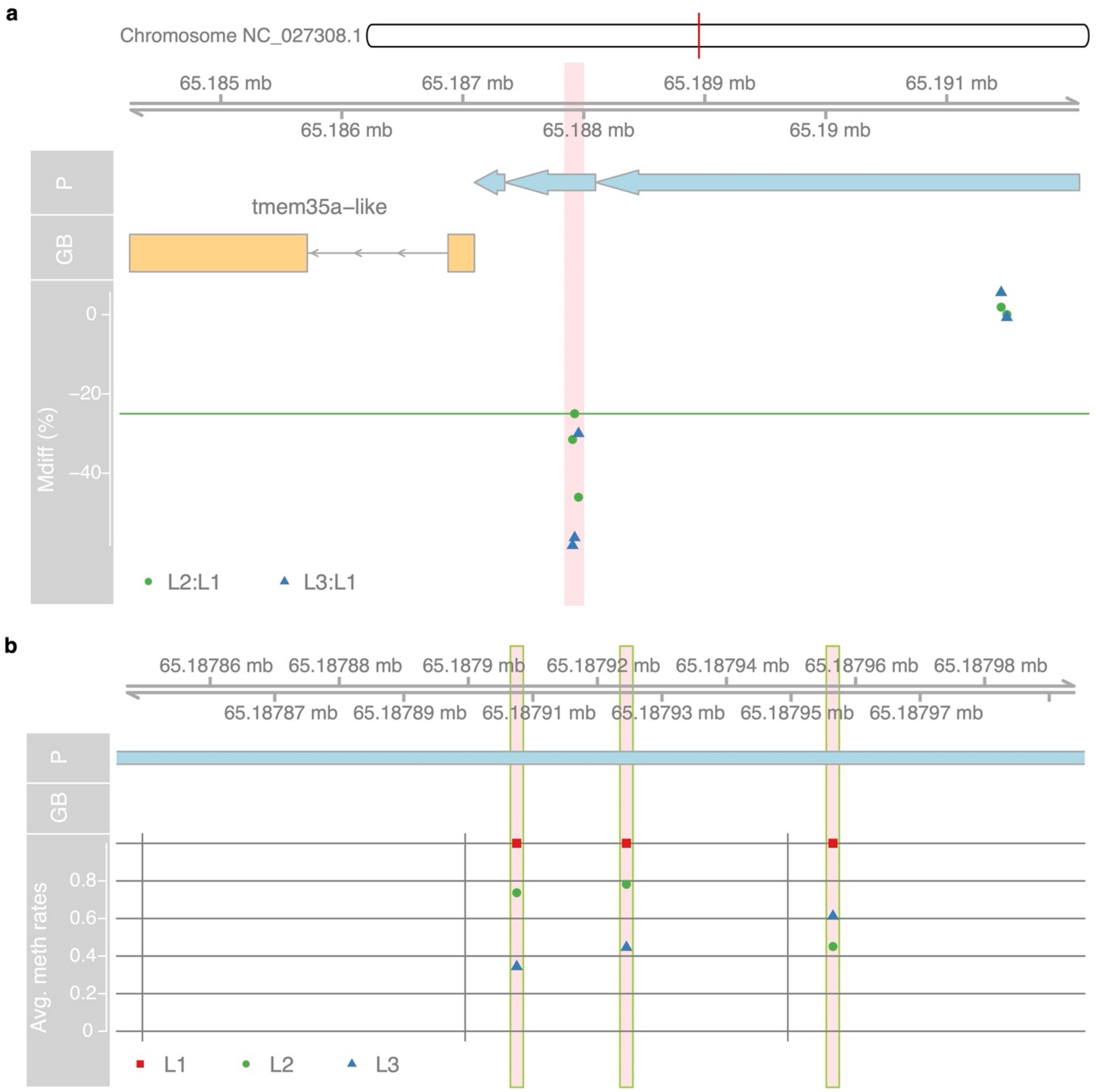

Figure S19. **Genomic feature view of *tmem35a-like* with three common DMCs in P1K**.
Visualization of genomic data of the *transmembrane protein 35-like*, (*tmem35a-like*, *LOC106611715*) locus and its vicinity provides information of genomic features along with methylation differences and methylation rates. **a** The main track at the bottom shows differences of methylation rates for L2:L1 and L3:L1. The green line indicates a threshold of 25%. **b** An enlarged view shows the part of the region indicated as a pink rectangle in **a**. The main track at the bottom shows average methylation rates of L1, L2, and L3. Three common DMCs between L2:L1 and L3:L1 are highlighted with pink vertical bars.

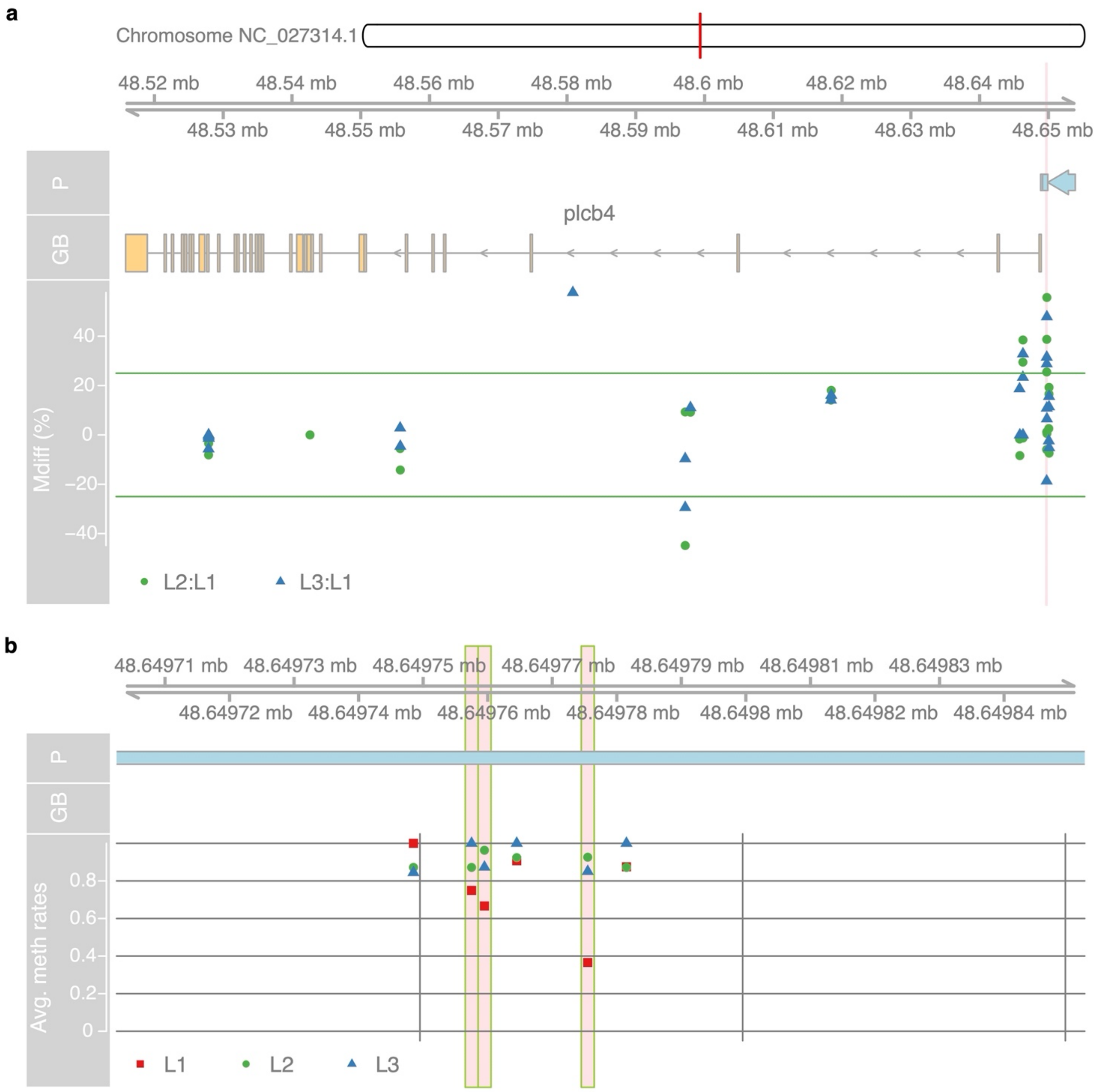

Figure S20. **Genomic feature view of *plcb4* with three common DMCs in P1K**.
Visualization of genomic data of the *phospholipase C beta 4* (*plcb4*) locus and its vicinity provides information of genomic features along with methylation differences and methylation rates. **a** The main track at the bottom shows differences of methylation rates for L2:L1 and L3:L1. Two green lines indicate thresholds of 25%. **b** An enlarged view shows the part of the region indicated as a pink rectangle in **a**. The main track at the bottom shows average methylation rates of L1, L2, and L3. Three common DMCs between L2:L1 and L3:L1 are highlighted with pink vertical bars.

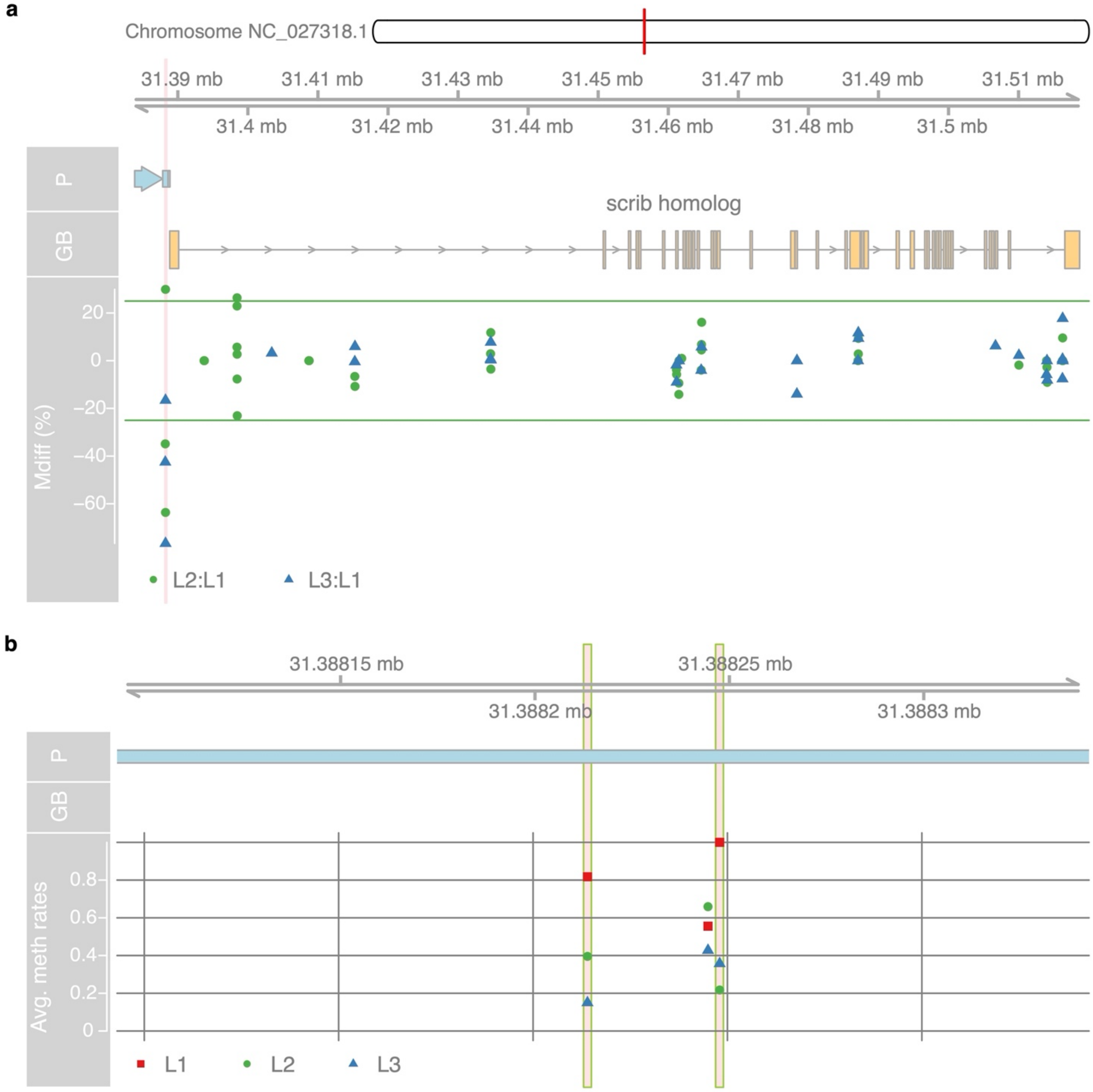

Figure S21. **Genomic feature view of *scrib homolog* with two common DMCs in P1K**.
Visualization of genomic data of the *protein scribble homolog* (*scrib homolog*, *LOC106578665*) locus and its vicinity provides information of genomic features along with methylation differences and methylation rates. **a** The main track at the bottom shows differences of methylation rates for L2:L1 and L3:L1. Two green lines indicate thresholds of 25%. **b** An enlarged view shows the part of the region indicated as a pink rectangle in **a**. The main track at the bottom shows average methylation rates of L1, L2, and L3. Two common DMCs between L2:L1 and L3:L1 are highlighted with pink vertical bars.

Supplementary tables

Table S1. Added micronutrient concentrations (mg/kg) within the NP.

Nutrient group	Micronutrient	L1	L2	L3
**Vitamin**	Vitamin A	3.79	7.58	15.16
	Vitamin D3	0.05	0.1	0.2
	Vitamin E	102.4	204.9	409.8
	Vitamin K3	9.82	19.64	39.28
	Thiamine (B1)	2.67	5.34	10.68
	Riboflavin (B2)	8.3	16.6	33.2
	B6	4.77	9.54	19.08
	B12	0.25	0.5	1
	Niacin (B3)	24.8	49.6	99.2
	Pantothenic Acid (B5)	17.15	34.3	68.6
	Folic Acid (B9)	2.82	5.64	11.28
	Biotin (B7)	0.14	0.28	0.56
	Vitamin C	80	160	320
**Micro-mineral**	Cobalt (Co)	0.94	1.88	3.76
	Iodine	0.67	1.34	2.68
	Selenium	0.23	0.46	0.92
	Iron	32.64	65.28	130.6
	Manganese	12.03	24.06	48.12
	Copper	3.24	6.48	12.96
	Zinc	66.92	133.8	267.7
**Macro-mineral**	Calcium (Ca)	0.4	0.8	1.6
**Amino acid**	Taurine	2450	4900	9800
	Histidine	1400	2800	5600
**Cholesterol**	Cholesterol	1100	2200	4400

Table S2. Analysed concentrations of the experimental diets.

Nutrient group	Unit	Micronutrient	Graded level[†]	L1	L2	L3	NRC 2011[‡]
**Vitamin**	mg/kg	Vitamin A	Yes	3.73	5.15	12.16	0.75[a]
		Vitamin D3	Yes	0.15	0.19	0.19	0.04[a]
		Vitamin E	Yes	241.5	364	436.5	60[b]
		Vitamin K3	Yes	0.71	1.51	2.7	<10[b]
		Thiamin (B1)	Yes	4.5	7.1	8.8	1[a]
		Riboflavin (B2)	Yes	17.2	27.8	33.5	4[a]
		Vitamin B6	Yes	12.8	16.8	21.3	5[b]
		Vitamin B12	Yes	0.18	0.35	0.67	NT
		Niacin (B3)	Yes	73	112	148	10[a]
		Pantothenic acid (B5)	Yes	24	58	44	20[a]
		Folic acid (B9)	Yes	6.53	9.69	11.67	1[a]
		Biotin (B7)	Yes	0.51	0.72	0.74	0.15[a]
		Vitamin C	Yes	183	251	409	20[b]
**Micro-mineral**	mg/kg	Cobalt	Yes	0.18	0.22	0.32	NT
		Iodine	Yes	n.a	n.a	n.a	1.1[a]
		Selenium	Yes	1.13	1.48	1.65	0.15[a]
		Iron	Yes	330	358	403	30-60[b]
		Manganese	Yes	42	53	86	10[b]
		Copper	Yes	11.8	14.8	22.8	5[b]
		Zinc	Yes	94	156	330	37[b]
**Macro-mineral**	g/kg	Calcium	Yes	6.7	7.1	8.2	NR[*b]
		Magnesium	No	1.73	1.66	1.68	0.4[b]
		Phosphorus	No	12.7	12.5	12.5	8.0[b]
**Amino acid**	g/kg	Taurine	Yes	2.6	4.4	10.1	NR[b]
		Methionine	No	9.7	9.9	10.3	7.0[b]
		Histidine	Yes	11.4	13.1	17.1	8.0[b]
**Cholesterol**		Cholesterol	Yes	n.a	n.a	n.a	NR

[†]Nutrients added at graded levels to the feeds. [‡]Current NRC, 2011, minimum requirement recommendations determined in [a] rainbow trout and [b]Atlantic salmon. Non-numeric values represent; n.a (not analysed), NR (no requirement), NR* (no requirement freshwater), and NT (not tested).

Table S3. Growth measures (mean ± SEM) recorded at the smolt and final stages.

Stage	Measurement	Unit	L1	L2	L3
**Smolt**	Body weight	g	68.0 ± 0.5[c]	77.4 ± 0.5[a]	73.8 ± 0.8[b]
	Condition Factor (K)	g/cm^3	1.21 ± 0.00	1.19 ± 0.00	1.19 ± 0.00
	Hepatosomatic index (HIS)	%	1.28 ± 0.06[a]	1.04 ± 0.01[b]	1.15 ± 0.06[b]
**Final**	Body weight	g	2127 ± 59[b]	2381 ± 82[a]	2385 ± 37[a]
	Condition Factor (K)	g/cm^3	1.37 ± 0.01[b]	1.43 ± 0.02[ab]	1.46 ± 0.03[a]
	Hepatosomatic index (HIS)	%	1.08 ± 0.06	1.02 ± 0.01	1.10 ± 0.04

[a,b,c]Superscripts denote significant differences between diets ($p < 0.05$, one-way ANOVA).

Table S4. Gonad RNA-seq samples with mapped counts.

No	Name	Diet	Sex	Total	Uniquely mapped	(%)	Multi-mapped	(%)	Unmapped	(%)
**1**	G1	L2	M	26 378 556	21 306 150	(80.8)	4 055 175	(15.4)	1 017 231	(3.9)
**2**	G6	L3	M	27 458 498	22 229 841	(81.0)	4 121 653	(15.0)	1 107 004	(4.0)
**3**	G9	L3	M	29 411 156	23 815 915	(81.0)	4 480 828	(15.2)	1 114 413	(3.8)
**4**	G11	L1	M	28 088 718	22 811 576	(81.2)	4 216 690	(15.0)	1 060 452	(3.8)
**5**	G18	L2	M	29 080 321	23 387 000	(80.4)	4 542 123	(15.6)	1 151 198	(4.0)
**6**	G21	L1	M	34 768 040	27 941 556	(80.4)	5 150 222	(14.8)	1 676 262	(4.8)
**7**	G22	L1	M	25 170 526	20 305 338	(80.7)	3 864 995	(15.4)	1 000 193	(4.0)
**8**	G23	L1	M	26 152 959	21 130 211	(80.8)	4 056 076	(15.5)	966 672	(3.7)
**9**	G24	L1	M	25 584 752	20 432 489	(79.9)	4 079 550	(15.9)	1 072 713	(4.2)
**10**	G26	L3	M	21 327 379	17 146 068	(80.4)	3 328 143	(15.6)	853 168	(4.0)
**11**	G27	L3	M	27 890 051	22 464 723	(80.5)	4 302 519	(15.4)	1 122 809	(4.0)
**12**	G31	L2	M	24 395 132	19 572 571	(80.2)	3 837 263	(15.7)	985 298	(4.0)
**13**	G34	L2	M	25 724 698	20 836 053	(81.0)	4 006 385	(15.6)	882 260	(3.4)
**14**	G35	L2	M	24 851 046	20 064 404	(80.7)	3 874 806	(15.6)	911 836	(3.7)
**15**	G36	L3	M	24 162 507	19 604 499	(81.1)	3 689 212	(15.3)	868 796	(3.6)
**16**	G40	L3	M	25 926 574	20 993 937	(81.0)	3 970 709	(15.3)	961 928	(3.7)
**17**	G41	L1	M	24 651 958	19 821 530	(80.4)	3 868 502	(15.7)	961 926	(3.9)

Table S5. Liver RNA-seq samples with mapped counts.

No	Name	Diet	Sex	Total reads	Uniquely mapped	(%)	Multi-mapped	(%)	Unmapped	(%)
**1**	L1	L2	M	23 931 936	19 343 294	(80.8)	4 069 114	(17.0)	519 528	(2.2)
**2**	L6	L3	M	25 784 227	20 835 554	(80.8)	4 342 016	(16.8)	606 657	(2.4)
**3**	L9	L3	M	24 515 643	18 958 733	(77.3)	5 050 907	(20.6)	506 003	(2.1)
**4**	L11	L1	M	35 226 602	28 614 343	(81.2)	5 829 665	(16.5)	782 594	(2.2)
**5**	L18	L2	M	26 964 928	21 640 070	(80.3)	4 794 142	(17.8)	530 716	(2.0)
**6**	L21	L1	M	18 942 816	15 243 947	(80.5)	3 221 854	(17.0)	477 015	(2.5)
**7**	L22	L1	M	23 089 367	18 614 587	(80.6)	3 987 283	(17.3)	487 497	(2.1)
**8**	L23	L1	M	25 798 922	20 938 468	(81.2)	4 259 572	(16.5)	600 882	(2.3)
**9**	L24	L1	M	24 147 183	19 158 542	(79.3)	4 463 935	(18.5)	524 706	(2.2)
**10**	L26	L3	M	22 126 381	17 433 239	(78.8)	4 151 966	(18.8)	541 176	(2.4)
**11**	L27	L3	M	23 382 289	18 723 912	(80.1)	4 150 950	(17.8)	507 427	(2.2)
**12**	L31	L2	M	22 905 555	17 692 154	(77.2)	4 071 464	(17.8)	1 141 937	(5.0)
**13**	L32	L2	M	24 849 856	20 002 667	(80.5)	4 290 325	(17.3)	556 864	(2.2)
**14**	L34	L2	M	25 064 047	19 818 939	(79.1)	4 630 819	(18.5)	614 289	(2.5)
**15**	L35	L2	M	26 770 780	21 178 280	(79.1)	4 109 368	(15.4)	1 483 132	(5.5)
**16**	L36	L3	M	23 220 436	18 513 605	(79.7)	4 213 806	(18.1)	493 025	(2.1)
**17**	L40	L3	M	26 161 249	21 043 381	(80.4)	4 516 363	(17.3)	601 505	(2.3)
**18**	L41	L1	M	23 952 553	19 128 270	(79.9)	4 378 012	(18.3)	446 271	(1.9)

Table S6. Liver DEGs - Enriched KEGG pathways by ORA.

Data set	Main class[a]	Sub class[b]	n[c]	Enriched pathways[d]
**L2:L1**	Metabolism	Lipid metabolism	1	**Steroid biosynthesis (sasa00100)**
		Metabolism of terpenoids and polyketides	1	**Terpenoid backbone biosynthesis (sasa00900)**
**L3:L1**	Metabolism	Lipid metabolism	3	**Steroid biosynthesis (sasa00100)**, Biosynthesis of unsaturated fatty acids (sasa01040), Steroid hormone biosynthesis (sasa00140)
		Global and overview maps	2	Fatty acid metabolism (sasa01212), Carbon metabolism (sasa01200)
		Metabolism of other amino acids	1	Glutathione metabolism (sasa00480)
		Metabolism of terpenoids and polyketides	1	**Terpenoid backbone biosynthesis (sasa00900)**
	Organismal Systems	Organismal Systems, Endocrine system	1	PPAR signaling pathway (sasa03320)

[ab]Main classes and sub classes of KEGG pathways defined on the KEGG website (https://www.genome.jp/kegg; br08901). [c]Number of enriched pathways. [d]Names of enriched KEGG pathways (adjusted p-values < 0.05 and minimum gene counts > 4) together with KEGG IDs in paratheses. Pathways in bold are identified in both L2:L1 and L3:L1.

Table S7. G&L DEGs - Enriched KEGG pathways by ORA.

Data set	Main class[a]	Sub class[b]	n[c]	Enriched pathways[d]
**L3:L1**	Metabolism	Amino acid metabolism	1	Glycine, serine and threonine metabolism (sasa00260)
		Lipid metabolism	1	Steroid biosynthesis (sasa00100)
		Metabolism of terpenoids and polyketides	1	Terpenoid backbone biosynthesis (sasa00900)

[ab]Main classes and sub classes of KEGG pathways defined on the KEGG website (https://www.genome.jp/kegg; br08901). [c]Number of enriched pathways. [d]Names of enriched KEGG pathways (adjusted p-values < 0.05 and minimum gene counts > 4) together with KEGG IDs in paratheses.

Table S8. Liver DEGs - Enriched GO terms.

Set	Root[a]	# Terms	Enriched terms[b]
**L2:L1**	BP	10	lipid biosynthetic process (GO:0008610), steroid metabolic process (GO:0008202), isoprenoid biosynthetic process (GO:0008299), isoprenoid metabolic process (GO:0006720), sterol metabolic process (GO:0016125), steroid biosynthetic process (GO:0006694), organic hydroxy compound metabolic process (GO:1901615), lipid metabolic process (GO:0006629), organic hydroxy compound biosynthetic process (GO:1901617), cellular lipid metabolic process (GO:0044255)
	MF	8	monooxygenase activity (GO:0004497), cofactor binding (GO:0048037), oxidoreductase activity, acting on paired donors, with incorporation or reduction of molecular oxygen (GO:0016705), heme binding (GO:0020037), tetrapyrrole binding (GO:0046906), iron ion binding (GO:0005506), isomerase activity (GO:0016853), coenzyme binding (GO:0050662)
**L3:L1**	CC	6	endoplasmic reticulum (GO:0005783), extracellular space (GO:0005615), endoplasmic reticulum membrane (GO:0005789), endoplasmic reticulum subcompartment (GO:0098827), nuclear outer membrane-endoplasmic reticulum membrane network (GO:0042175), endoplasmic reticulum part (GO:0044432)
	BP	36	lipid metabolic process (GO:0006629), steroid metabolic process (GO:0008202), lipid biosynthetic process (GO:0008610), steroid biosynthetic process (GO:0006694), sterol metabolic process (GO:0016125), sterol biosynthetic process (GO:0016126), organic hydroxy compound metabolic process (GO:1901615), isoprenoid biosynthetic process (GO:0008299), isoprenoid metabolic process (GO:0006720), organic hydroxy compound biosynthetic process (GO:1901617), secondary alcohol metabolic process (GO:1902652), cellular lipid metabolic process (GO:0044255), cholesterol metabolic process (GO:0008203), alcohol metabolic process (GO:0006066), small molecule biosynthetic process (GO:0044283), alcohol biosynthetic process (GO:0046165), fatty acid metabolic process (GO:0006631), nucleotide metabolic process (GO:0009117), fatty acid biosynthetic process (GO:0006633), nucleobase-containing small molecule metabolic process (GO:0055086), nucleoside phosphate metabolic process (GO:0006753), monocarboxylic acid metabolic process (GO:0032787), coenzyme metabolic process (GO:0006732), phospholipid biosynthetic process (GO:0008654), nucleoside bisphosphate metabolic process (GO:0033865), ribonucleoside bisphosphate metabolic process (GO:0033875), purine nucleoside bisphosphate metabolic process (GO:0034032), organic acid biosynthetic process (GO:0016053), carboxylic acid biosynthetic process (GO:0046394), organophosphate biosynthetic process (GO:0090407), monocarboxylic acid biosynthetic process (GO:0072330), carboxylic acid metabolic process (GO:0019752), oxoacid metabolic process (GO:0043436), organic acid metabolic process (GO:0006082), ribose phosphate metabolic process (GO:0019693), cofactor metabolic process (GO:0051186)
	MF	16	monooxygenase activity (GO:0004497), iron ion binding (GO:0005506), oxidoreductase activity, acting on paired donors, with incorporation or reduction of molecular oxygen (GO:0016705), heme binding (GO:0020037), tetrapyrrole binding (GO:0046906), coenzyme binding (GO:0050662), oxidoreductase activity, acting on the CH-OH group of donors, NAD or NADP as acceptor (GO:0016616), oxidoreductase activity, acting on CH-OH group of donors (GO:0016614), oxidoreductase activity, acting on the CH-CH group of donors (GO:0016627), transferase activity, transferring alkyl or aryl (other than methyl) groups (GO:0016765), receptor ligand activity (GO:0048018), receptor regulator activity (GO:0030545), steroid hormone receptor activity (GO:0003707), nuclear receptor activity (GO:0004879), transcription factor activity, direct ligand regulated sequence-specific DNA binding (GO:0098531), flavin adenine dinucleotide binding (GO:0050660)

[a]CC (cellular component), BP (biological process), MF (molecular function). [b]Enriched terms are sorted by adjusted p-values with the most significant one listed first.

Table S9. G&L DEGs - Enriched GO terms.

Set	Root[a]	# Terms	Enriched terms[b]
**L2:L1**	BP	3	regulation of transcription by RNA polymerase II (GO:0006357), transcription by RNA polymerase II (GO:0006366), immune response (GO:0006955)
**L3:L1**	BP	10	steroid metabolic process (GO:0008202), sterol metabolic process (GO:0016125), organic hydroxy compound metabolic process (GO:1901615), sterol biosynthetic process (GO:0016126), lipid biosynthetic process (GO:0008610), steroid biosynthetic process (GO:0006694), organic hydroxy compound biosynthetic process (GO:1901617), small molecule biosynthetic process (GO:0044283), alcohol metabolic process (GO:0006066), cellular lipid metabolic process (GO:0044255)

[a]Root GO terms defined as CC (cellular component), BP (biological process), MF (molecular function). [b]Enriched terms are sorted by adjusted p-values with the most significant one listed first.

Table S10. Gonad DEGs - Enriched KEGG pathways by GSEA.

Data set	Main class[a]	Sub class[b]	n[c]	NES[d]	Enriched pathways[e]
**L1:L2**	Environmental Information Processing	Signaling molecules and interaction	3	Up	**Cytokine-cytokine receptor interaction (sasa04060)**, Cell adhesion molecules (sasa04514), ECM-receptor interaction (sasa04512)
	Organismal Systems	Immune system	3	Up	NOD-like receptor signaling pathway (sasa04621), Toll-like receptor signaling pathway (sasa04620), C-type lectin receptor signaling pathway (sasa04625)
	Genetic Information Processing	Replication and repair	2	Down	**DNA replication (sasa03030)**, **Mismatch repair (sasa03430)**
	Cellular Processes	Cell growth and death	1	Up	Apoptosis (sasa04210)
**L1:L3**	Genetic Information Processing	Replication and repair	3	Down	**DNA replication (sasa03030)**, **Mismatch repair (sasa03430)**, Nucleotide excision repair (sasa03420)
		Transcription	2	Down	RNA polymerase (sasa03020), Spliceosome (sasa03040)
		Translation	2	Down	Ribosome biogenesis in eukaryotes (sasa03008), RNA transport (sasa03013)
	Environmental Information Processing	Signaling molecules and interaction	1	Up	**Cytokine-cytokine receptor interaction (sasa04060)**
	Organismal Systems	Immune system	1	Up	Intestinal immune network for IgA production (sasa04672)

[ab]Main classes and sub classes of KEGG pathways defined on the KEGG website (https://www.genome.jp/kegg; br08901). [c]Number of enriched pathways. [d]"Down" indicates down-regulation as negative NES (normalized enrichment score) values, whereas "Up" indicates up-regulation as positive NES values. [e]Names of enriched KEGG pathways (adjusted p-values < 0.05, abs(NES) > 2, and minimum gene counts: 5) together with KEGG IDs in paratheses. Pathways in bold are identified in both L2:L1 and L3:L1.

Table S11. Liver DEGs - Enriched KEGG pathways by GSEA.

Data set	Main class[a]	Sub class[b]	n[c]	NES[d]	Enriched pathways[e]
**L2:L1**	Genetic Information Processing	Translation	1	Down	**Aminoacyl-tRNA biosynthesis (sasa00970)**
	Metabolism	Amino acid metabolism	1	Down	Glycine, serine and threonine metabolism (sasa00260)
		Lipid metabolism	1	Down	**Steroid biosynthesis (sasa00100)**
		Metabolism of terpenoids and polyketides	1	Down	**Terpenoid backbone biosynthesis (sasa00900)**
**L3:L1**	Metabolism	Lipid metabolism	4	Down	**Steroid biosynthesis (sasa00100)**, Biosynthesis of unsaturated fatty acids (sasa01040), alpha-Linolenic acid metabolism (sasa00592), Fatty acid biosynthesis (sasa00061)
		Global and overview maps	2	Down	Fatty acid metabolism (sasa01212), Carbon metabolism (sasa01200)
		Carbohydrate metabolism	1	Down	Propanoate metabolism (sasa00640)
		Energy metabolism	1	Down	Oxidative phosphorylation (sasa00190)
		Metabolism of terpenoids and polyketides	1	Down	**Terpenoid backbone biosynthesis (sasa00900)**
	Genetic Information Processing	Translation	3	Down	Ribosome biogenesis in eukaryotes (sasa03008), **Aminoacyl-tRNA biosynthesis (sasa00970)**, Ribosome (sasa03010)
		Replication and repair	2	Down	DNA replication (sasa03030), Mismatch repair (sasa03430)
		Folding, sorting and degradation	1	Down	Proteasome (sasa03050)

[ab]Main classes and sub classes of KEGG pathways defined on the KEGG website (https://www.genome.jp/kegg; br08901). [c]Number of enriched pathways. [d]"Down" indicates down-regulation as negative NES (normalized enrichment score) values. [e]Names of enriched KEGG pathways (adjusted p-values < 0.05, abs(NES) > 2, and minimum gene counts: 5) together with KEGG IDs in paratheses. Pathways in bold are identified in both L2:L1 and L3:L1.

Table S12. G&L DEGs - Enriched KEGG pathways by GSEA.

Data set	Main class[a]	Sub class[b]	n[c]	NES[d]	Enriched pathways[e]
**L1:L2**	Organismal Systems	Immune system	3	Up	NOD-like receptor signaling pathway (sasa04621), C-type lectin receptor signaling pathway (sasa04625), Intestinal immune network for IgA production (sasa04672)
	Environmental Information Processing	Signaling molecules and interaction	2	Up	**Cytokine-cytokine receptor interaction (sasa04060)**, **Cell adhesion molecules (sasa04514)**
	Genetic Information Processing	Translation	1	Down	**Ribosome biogenesis in eukaryotes (sasa03008)**
	Metabolism	Amino acid metabolism	1	Down	Glycine, serine and threonine metabolism (sasa00260)
		Lipid metabolism	1	Down	**Steroid biosynthesis (sasa00100)**
		Metabolism of terpenoids and polyketides	1	Down	**Terpenoid backbone biosynthesis (sasa00900)**
**L1:L3**	Genetic Information Processing	Replication and repair	6	Down	DNA replication (sasa03030), Mismatch repair (sasa03430), Base excision repair (sasa03410), Homologous recombination (sasa03440), Nucleotide excision repair (sasa03420), Fanconi anemia pathway (sasa03460)
		Translation	4	Down	**Ribosome biogenesis in eukaryotes (sasa03008)**, RNA transport (sasa03013), Aminoacyl-tRNA biosynthesis (sasa00970), Ribosome (sasa03010)
		Transcription	2	Down	RNA polymerase (sasa03020), Spliceosome (sasa03040)
		Folding, sorting and degradation	1	Down	Proteasome (sasa03050)
	Environmental Information Processing	Signaling molecules and interaction	2	Up	**Cell adhesion molecules (sasa04514)**, **Cytokine-cytokine receptor interaction (sasa04060)**
	Metabolism	Carbohydrate metabolism	2	Down	Propanoate metabolism (sasa00640), Glyoxylate and dicarboxylate metabolism (sasa00630)
		Global and overview maps	2	Down	Fatty acid metabolism (sasa01212), Carbon metabolism (sasa01200)
		Lipid metabolism	2	Down	**Steroid biosynthesis (sasa00100)**, alpha-Linolenic acid metabolism (sasa00592)
		Amino acid metabolism	1	Down	Valine, leucine and isoleucine degradation (sasa00280)
		Energy metabolism	1	Down	Oxidative phosphorylation (sasa00190)
		Metabolism of terpenoids and polyketides	1	Down	**Terpenoid backbone biosynthesis (sasa00900)**

[ab]Main classes and sub classes of KEGG pathways defined on the KEGG website (https://www.genome.jp/kegg; br08901). [c]Number of enriched pathways. [d]"Down" indicates down-regulation as negative NES (normalized enrichment score) values, whereas "Up" indicates up-regulation as positive NES values. [e]Names of enriched KEGG pathways (adjusted p-values < 0.05, abs(NES) > 2, and minimum gene counts: 5) together with KEGG IDs in paratheses. Pathways in bold are identified in both L2:L1 and L3:L1.

Table S13. Read alignment of 18 RRBS samples on the Salmon genome.

Tissue	Name	Diet	Total	#Uniq[a]	%Uniq[b]	#Aligned Cs[c]	#CpGs[d]	#Met CpGs[e]	%Met[f]
**Gonads**	G11	L1	59283240	27746518	46.80	290898403	21.17	51833315	84.18
	G24	L1	61127908	27555755	45.08	291698912	20.89	51025800	83.73
	G41	L1	59694373	26870194	45.01	285455262	20.98	50457461	84.25
	G1	L2	64870321	29632714	45.68	312654452	20.93	55112619	84.21
	G18	L2	54130662	25345593	46.82	272179158	20.82	47578801	83.96
	G31	L2	63529304	29501162	46.44	308612179	20.82	53859427	83.83
	G26	L3	58252584	27591273	47.36	290761590	20.93	50884006	83.61
	G27	L3	67963338	31948993	47.01	331512995	21.09	58562821	83.77
	G40	L3	62837150	29908799	47.60	314276128	20.97	55001291	83.45
**Liver**	L11	L1	68986028	33639758	48.76	353798160	20.89	61967075	83.85
	L22	L1	53584633	24645272	45.99	262714857	20.86	46393393	84.68
	L24	L1	49281151	23525443	47.74	247448574	21.00	43541086	83.79
	L1	L2	64938299	30959215	47.67	326750266	21.05	58303520	84.77
	L18	L2	70070490	33404199	47.67	353591304	20.87	62241040	84.34
	L32	L2	58784680	28289701	48.12	297408787	21.02	52823444	84.51
	L26	L3	55156176	26012826	47.16	276699136	20.94	48703187	84.07
	L36	L3	57119286	27490750	48.13	290047602	21.10	51824926	84.69
	L6	L3	61742432	28936883	46.87	307091088	20.93	54305524	84.51

[a]Number of uniquely aligned reads. [b]Percentage of uniquely aligned reads. [c]Number of Cs among the uniquely aligned regions. [d]Number of CpG sites among the aligned Cs. [e]Percentage of CpG sites among the aligned Cs. [f]Average methylation rate calculated with mapped CpG sites.

Table S14. Gonad DMGs L2:L1 - Enriched KEGG pathways by ORA.

Main class[a]	Sub class[b]	KEGG pathway[c]	KEGG ID	Genetic region (gene ratio)[d]
Cellular Processes	Cell growth and death	Cellular senescence	sasa04218	P5K (25/357)
	Cellular community - eukaryotes	**Adherens junction**	sasa04520	**RS+GB** (87/2818), P+GB (77/2311), **Gene body** (72/1958), Exon (25/570), **Intron** (55/1516)
		Gap junction	sasa04540	RS+GB (73/2818), P+GB (64/2311), Gene body (61/1958), Intron (45/1516)
Environmental Information Processing	Signal transduction	**ErbB signaling pathway**	sasa04012	**RS+GB** (83/2818), **P+GB** (73/2311), **Gene body** (66/1958), **Intron** (59/1516)
		Phosphatidylinositol signaling system	sasa04070	RS+GB (70/2818), P+GB (62/2311), Gene body (58/1958), Intron (47/1516)
		**Wnt signaling pathway**	sasa04310	**RS+GB** (129/2818), **P+GB** (112/2311), **Gene body** (98/1958)
		Apelin signaling pathway	sasa04371	RS+GB (102/2818), P+GB (91/2311), Gene body (77/1958), Intron (64/1516)
	Signaling molecules and interaction	**ECM-receptor interaction**	sasa04512	**RS+GB** (78/2818), **P+GB** (70/2311), **Gene body** (63/1958), **Exon** (23/570), **I**ntron (50/1516)
		**Cell adhesion molecules**	sasa04514	**P+GB** (98/2311), **Gene body** (86/1958), Intron (72/1516)
Metabolism	Carbohydrate metabolism	Inositol phosphate metabolism	sasa00562	RS+GB (55/2818), Gene body (42/1958)
	Glycan biosynthesis and metabolism	Mucin type O-glycan biosynthesis	sasa00512	P+GB (25/2311)
		**Various types of N-glycan biosynthesis**	sasa00513	Gene body (25/1958), **Intron** (23/1516)
		Other types of O-glycan biosynthesis	sasa00514	RS+GB (38/2818), P+GB (32/2311), Gene body (27/1958)
		**Glycosaminoglycan biosynthesis - heparan sulfate / heparin**	sasa00534	**RS+GB** (29/2818), **P+GB** (28/2311), **Gene body** (26/1958), **Intron** (24/1516)
	Lipid metabolism	Glycerolipid metabolism	sasa00561	RS+GB (43/2818), P+GB (37/2311), Gene body (31/1958)
Organismal Systems	Circulatory system	**Adrenergic signaling in cardiomyocytes**	sasa04261	**RS+GB** (160/2818), **P+GB** (139/2311), **Gene body** (122/1958), **Intron** (104/1516)
		Vascular smooth muscle contraction	sasa04270	RS+GB (89/2818), P+GB (76/2311), Gene body (66/1958)
	Endocrine system	Insulin signaling pathway	sasa04910	P+GB (80/2311)
		**GnRH signaling pathway**	sasa04912	RS+GB (83/2818), P+GB (76/2311), Gene body (69/1958), **Intron** (57/1516)
		Melanogenesis	sasa04916	RS+GB (90/2818), P+GB (80/2311), Gene body (72/1958), Intron (53/1516)

[ab]Main classes and sub classes of KEGG pathways defined on the KEGG website (https://www.genome.jp/kegg; br08901). [c]Names of enriched KEGG pathways (adjusted p-values < 0.05 and minimum gene counts > 20) together with KEGG IDs in paratheses. [d]Genetic regions - RS: regularly sequence (gene body + promoter + flanks), P: promoter (P250 + P1K + P5K), GB: gene body (intron + exon) - with gene ratio (# of DMGs in pathways / # of all DMGs).

Table S15. Gonad DMGs L3:L1 - Enriched KEGG pathways by ORA.

Main class[a]	Sub class[b]	KEGG pathway	KEGG ID	Genetic region (gene ratio)[c]
Cellular Processes	Cellular community - eukaryotes	**Adherens junction**	sasa04520	**RS+GB** (78/2725), **P+GB** (68/2219), **Gene body** (60/1898), **Intron** (49/1490)
Environmental Information Processing	Signal transduction	**ErbB signaling pathway**	sasa04012	**RS+GB** (78/2725), **P+GB** (65/2219), **Gene body** (61/1898), **Intron** (54/1490)
		**Wnt signaling pathway**	sasa04310	**RS+GB** (125/2725), **P+GB** (106/2219), **Gene body** (91/1898)
	Signaling molecules and interaction	**ECM-receptor interaction**	sasa04512	**RS+GB** (66/2725), **P+GB** (61/2219), **Gene body** (58/1898), **Exon** (23/545), **Intron** (42/1490)
		**Cell adhesion molecules**	sasa04514	**P+GB** (96/2219), **Gene body** (87/1898), **Intron** (73/1490)
Metabolism	Amino acid metabolism	Lysine degradation	sasa00310	Exon (15/545)
	Glycan biosynthesis and metabolism	**Various types of N-glycan biosynthesis**	sasa00513	**Intron** (24/1490)
		**Glycosaminoglycan biosynthesis - heparan sulfate / heparin**	sasa00534	RS+GB (31/2725), **P+GB** (27/2219), **Gene body** (25/1898), **Intron** (22/1490)
	Nucleotide metabolism	Purine metabolism	sasa00230	Gene body (65/1898)
Organismal Systems	Circulatory system	**Adrenergic signaling in cardiomyocytes**	sasa04261	RS+GB (133/2725), **P+GB** (118/2219), **Gene body** (103/1898), **Intron** (83/1490)
	Endocrine system	**GnRH signaling pathway**	sasa04912	**Intron** (47/1490)
		Adipocytokine signaling pathway	sasa04920	P+GB (49/2219), Intron (34/1490)

[ab]Main classes and sub classes of KEGG pathways defined on the KEGG website (https://www.genome.jp/kegg; br08901). [c]Names of enriched KEGG pathways (adjusted p-values < 0.05 and minimum gene counts > 4) together with KEGG IDs in paratheses. [d]Genetic regions - RS: regularly sequence (gene body + promoter + flanks), P: promoter (P250 + P1K + P5K), GB: gene body (intron + exon) - with gene ratio (# of DMGs in pathways / # of all DMGs).

Table S16. Gonad DMGs L2:L1 - Enriched GO terms by ORA.

Root[a]	Region	# Terms	Top 5 enriched terms (terms with five lowest adjusted p-values)
**BP**	Exon	5	regulation of Ras protein signal transduction (GO:0046578), regulation of small GTPase mediated signal transduction (GO:0051056), cell-cell adhesion via plasma-membrane adhesion molecules (GO:0098742), homophilic cell adhesion via plasma membrane adhesion molecules (GO:0007156), Ras protein signal transduction (GO:0007265)
	Gene body	31	regulation of small GTPase mediated signal transduction (GO:0051056), regulation of Ras protein signal transduction (GO:0046578), Ras protein signal transduction (GO:0007265), regulation of intracellular signal transduction (GO:1902531), Rho protein signal transduction (GO:0007266)
	Intron	30	regulation of small GTPase mediated signal transduction (GO:0051056), regulation of Ras protein signal transduction (GO:0046578), Ras protein signal transduction (GO:0007265), regulation of intracellular signal transduction (GO:1902531), Rho protein signal transduction (GO:0007266)
	P+GB	26	regulation of small GTPase mediated signal transduction (GO:0051056), regulation of Ras protein signal transduction (GO:0046578), Ras protein signal transduction (GO:0007265), regulation of intracellular signal transduction (GO:1902531), Rho protein signal transduction (GO:0007266)
	RS+GB	18	regulation of small GTPase mediated signal transduction (GO:0051056), regulation of Ras protein signal transduction (GO:0046578), Ras protein signal transduction (GO:0007265), regulation of intracellular signal transduction (GO:1902531), Rho protein signal transduction (GO:0007266)
**CC**	Gene body	16	synapse (GO:0045202), synapse part (GO:0044456), neuron part (GO:0097458), postsynaptic membrane (GO:0045211), synaptic membrane (GO:0097060)
	Intron	15	synapse (GO:0045202), synapse part (GO:0044456), neuron part (GO:0097458), postsynaptic membrane (GO:0045211), synaptic membrane (GO:0097060)
	P+GB	15	synapse (GO:0045202), synapse part (GO:0044456), postsynaptic membrane (GO:0045211), synaptic membrane (GO:0097060), neuron part (GO:0097458)
	RS+GB	13	synapse (GO:0045202), synapse part (GO:0044456), postsynaptic membrane (GO:0045211), synaptic membrane (GO:0097060), postsynapse (GO:0098794)
**MF**	Exon	7	guanyl-nucleotide exchange factor activity (GO:0005085), Ras GTPase binding (GO:0017016), extracellular matrix structural constituent (GO:0005201), small GTPase binding (GO:0031267), Rho GTPase binding (GO:0017048)
	Gene body	49	guanyl-nucleotide exchange factor activity (GO:0005085), Ras guanyl-nucleotide exchange factor activity (GO:0005088), Rho GTPase binding (GO:0017048), Ras GTPase binding (GO:0017016), Rho guanyl-nucleotide exchange factor activity (GO:0005089)
	Intron	50	guanyl-nucleotide exchange factor activity (GO:0005085), GTPase activator activity (GO:0005096), GTPase regulator activity (GO:0030695), Rho GTPase binding (GO:0017048), Ras guanyl-nucleotide exchange factor activity (GO:0005088)
	P+GB	43	guanyl-nucleotide exchange factor activity (GO:0005085), Ras guanyl-nucleotide exchange factor activity (GO:0005088), Rho guanyl-nucleotide exchange factor activity (GO:0005089), Rho GTPase binding (GO:0017048), Ras GTPase binding (GO:0017016)
	RS+GB	33	guanyl-nucleotide exchange factor activity (GO:0005085), Ras guanyl-nucleotide exchange factor activity (GO:0005088), Rho guanyl-nucleotide exchange factor activity (GO:0005089), Rho GTPase binding (GO:0017048), Ras GTPase binding (GO:0017016)

[a]Root GO terms defined as CC (cellular component), BP (biological process), MF (molecular function).

Table S17. Gonad DMGs L3:L1 - Enriched GO terms by ORA.

Root[a]	Region	# Terms	Top 5 enriched terms (terms with five lowest adjusted p-values)
**BP**	Gene body	26	regulation of small GTPase mediated signal transduction (GO:0051056), regulation of Ras protein signal transduction (GO:0046578), Ras protein signal transduction (GO:0007265), Rho protein signal transduction (GO:0007266), regulation of Rho protein signal transduction (GO:0035023)
	Intron	35	regulation of small GTPase mediated signal transduction (GO:0051056), Ras protein signal transduction (GO:0007265), regulation of Ras protein signal transduction (GO:0046578), regulation of intracellular signal transduction (GO:1902531), regulation of transmembrane transport (GO:0034762)
	P+GB	34	regulation of small GTPase mediated signal transduction (GO:0051056), regulation of Ras protein signal transduction (GO:0046578), Ras protein signal transduction (GO:0007265), Rho protein signal transduction (GO:0007266), regulation of Rho protein signal transduction (GO:0035023)
	RS+GB	26	regulation of small GTPase mediated signal transduction (GO:0051056), regulation of transmembrane transport (GO:0034762), regulation of ion transmembrane transport (GO:0034765), nervous system development (GO:0007399), Ras protein signal transduction (GO:0007265)
**CC**	Gene body	13	cation channel complex (GO:0034703), synapse (GO:0045202), synapse part (GO:0044456), ion channel complex (GO:0034702), transmembrane transporter complex (GO:1902495)
	Intron	14	synapse (GO:0045202), synapse part (GO:0044456), cation channel complex (GO:0034703), transmembrane transporter complex (GO:1902495), ion channel complex (GO:0034702)
	P+GB	14	cation channel complex (GO:0034703), synapse (GO:0045202), synapse part (GO:0044456), ion channel complex (GO:0034702), neuron part (GO:0097458)
	RS+GB	11	cation channel complex (GO:0034703), ion channel complex (GO:0034702), transmembrane transporter complex (GO:1902495), transporter complex (GO:1990351), synapse (GO:0045202)
**MF**	Gene body	40	guanyl-nucleotide exchange factor activity (GO:0005085), Ras GTPase binding (GO:0017016), small GTPase binding (GO:0031267), Ras guanyl-nucleotide exchange factor activity (GO:0005088), Rho guanyl-nucleotide exchange factor activity (GO:0005089)
	Intron	40	guanyl-nucleotide exchange factor activity (GO:0005085), GTPase activator activity (GO:0005096), GTPase regulator activity (GO:0030695), Ras GTPase binding (GO:0017016), nucleoside-triphosphatase regulator activity (GO:0060589)
	P+GB	34	guanyl-nucleotide exchange factor activity (GO:0005085), small GTPase binding (GO:0031267), Ras GTPase binding (GO:0017016), Ras guanyl-nucleotide exchange factor activity (GO:0005088), Rho guanyl-nucleotide exchange factor activity (GO:0005089)
	RS+GB	25	voltage-gated ion channel activity (GO:0005244), voltage-gated channel activity (GO:0022832), guanyl-nucleotide exchange factor activity (GO:0005085), voltage-gated cation channel activity (GO:0022843), voltage-gated potassium channel activity (GO:0005249)

[a]Root GO terms defined as CC (cellular component), BP (biological process), MF (molecular function).

Table S18. Liver DMGs L2:L1 - Enriched KEGG pathways by ORA.

**Main class**[a]	**Sub class**[b]	**KEGG pathway**	**KEGG ID**	**Genetic region (gene ratio)** [c]
Cellular Processes	Cell growth and death	Cellular senescence	sasa04218	P+GB (88/2123)
	Cellular community - eukaryotes	**Adherens junction**	sasa04520	**RS+GB (**75/2652), P+GB (65/2123), **Gene body** (61/1822), **Intron** (50/1443)
		Tight junction	sasa04530	Intron (78/1443)
		**Gap junction**	sasa04540	**RS+GB** (74/2652), **P+GB** (65/2123), **Gene body** (55/1822), Intron (43/1443)
Environmental Information Processing	Membrane transport	**ABC transporters**	sasa02010	**RS+GB** (25/2652), **P+GB** (22/2123), **Gene body** (20/1822)
	Signal transduction	**ErbB signaling pathway**	sasa04012	**RS+GB** (71/2652), **P+GB** (62/2123), **Gene body** (56/1822), **Intron** (47/1443)
		**Wnt signaling pathway**	sasa04310	**RS+GB** (117/2652), **P+GB** (98/2123), **Gene body** (90/1822)
		**Apelin signaling pathway**	sasa04371	**RS+GB** (104/2652), **P+GB** (90/2123), **Gene body** (80/1822), **Intron** (73/1443)
	Signaling molecules and interaction	**ECM-receptor interaction**	sasa04512	**RS+GB** (83/2652), **P+GB** (75/2123), **Gene body** (72/1822), **Exon** (26/504), **Intron** (57/1443)
		**Cell adhesion molecules**	sasa04514	**RS+GB** (111/2652**), P+GB** (92/2123), **Gene body** (80/1822), **Intron** (69/1443)
Metabolism	Glycan biosynthesis and metabolism	**Mucin type O-glycan biosynthesis**	sasa00512	**P+GB** (26/2123), **Gene body** (24/1822)
		**Glycosaminoglycan biosynthesis - heparan sulfate / heparin**	sasa00534	**RS+GB** (24/2652), **P+GB** (23/2123), **Gene body** (22/1822), **Intron** (20/1443)
Organismal Systems	Circulatory system	**Adrenergic signaling in cardiomyocytes**	sasa04261	**RS+GB** (145/2652), **P+GB** (124/2123), **Gene body** (112/1822), **Intron** (93/1443)
		**Vascular smooth muscle contraction**	sasa04270	**RS+GB** (91/2652), P+GB (72/2123), Gene body (66/1822), Intron (57/1443)
	Endocrine system	**GnRH signaling pathway**	sasa04912	**P+GB** (63/2123), **Gene body** (56/1822), **Intron** (45/1443)
		Adipocytokine signaling pathway	sasa04920	P+GB (45/2123), Gene body (42/1822), Intron (37/1443)

[ab]Main classes and sub classes of KEGG pathways defined on the KEGG website (https://www.genome.jp/kegg; br08901). [c]Names of enriched KEGG pathways (adjusted p-values < 0.05 and minimum gene counts > 4) together with KEGG IDs in paratheses. [d]Genetic regions - RS: regularly sequence (gene body + promoter + flanks), P: promoter (P250 + P1K + P5K), GB: gene body (intron + exon) - with gene ratio (# of DMGs in pathways / # of all DMGs).

Table S19. Liver DMGs L3:L1 - Enriched KEGG pathways by ORA.

Main class[a]	Sub class[b]	KEGG pathway	KEGG ID	Genetic region (gene ratio)[c]
Cellular Processes	Cellular community - eukaryotes	**Adherens junction**	sasa04520	**RS+GB** (71/2725), **P+GB** (61/2226), **Gene body** (57/1879), **Intron** (52/1486)
	Cellular community - eukaryotes	**Gap junction**	sasa04540	**RS+GB** (71/2725), **P+GB** (60/2226), **Gene body** (53/1879)
Environmental Information Processing	Membrane transport	**ABC transporters**	sasa02010	**RS+GB** (29/2725), P+GB (27/2226), **Gene body** (26/1879), Intron (20/1486)
	Signal transduction	**ErbB signaling pathway**	sasa04012	**RS+GB** (75/2725), **P+GB** (64/2226), **Gene body** (60/1879), **Intron** (48/1486)
		Phosphatidylinositol signaling system	sasa04070	RS+GB (64/2725), P+GB (56/2226), Gene body (53/1879), Intron (52/1486)
		Adrenergic signaling in cardiomyocytes	sasa04261	RS+GB (129/2725), P+GB (115/2226), Gene body (103/1879), Intron (89/1486)
		**Wnt signaling pathway**	sasa04310	**RS+GB** (121/2725), **P+GB** (103/2226), **Gene body** (90/1879), Intron (73/1486)
		**Apelin signaling pathway**	sasa04371	**RS+GB** (111/2725), **P+GB** (96/2226), **Gene body** (79/1879), **Intron** (68/1486)
	Signaling molecules and interaction	**ECM-receptor interaction**	sasa04512	**RS+GB** (89/2725), **P+GB** (79/2226), **Gene body** (77/1879), **Exon** (29/520), **Intron** (59/1486)
		**Cell adhesion molecules**	sasa04514	**RS+GB** (115/2725), **P+GB** (99/2226), **Gene body** (87/1879), **Intron** (76/1486)
Metabolism	Carbohydrate metabolism	Inositol phosphate metabolism	sasa00562	Gene body (39/1879), Intron (36/1486)
	Glycan biosynthesis and metabolism	N-Glycan biosynthesis	sasa00510	P+GB (32/2226), Gene body (29/1879), Intron (25/1486)
		**Mucin type O-glycan biosynthesis**	sasa00512	RS+GB (28/2725), **P+GB** (25/2226), **Gene body** (22/1879), Intron (18/1486)
		Various types of N-glycan biosynthesis	sasa00513	Intron (23/1486)
		**Glycosaminoglycan biosynthesis - heparan sulfate / heparin**	sasa00534	**RS+GB** (25/2725), **P+GB** (23/2226), **Gene body** (23/1879), **Intron** (19/1486)
	Lipid metabolism	Fatty acid biosynthesis	sasa00061	P+GB (14/2226), Gene body (13/1879)
Organismal Systems	Circulatory system	**Adrenergic signaling in cardiomyocytes**	sasa04261	**RS+GB** (129/2725), **P+GB** (115/2226), **Gene body** (103/1879), **Intron** (89/1486)
		**Vascular smooth muscle contraction**	sasa04270	**RS+GB** (85/2725)
	Endocrine system	**GnRH signaling pathway**	sasa04912	RS+GB (74/2725), **P+GB** (69/2226), **Gene body** (63/1879), **Intron** (49/1486)
		Melanogenesis	sasa04916	RS+GB (84/2725), P+GB (69/2226), Gene body (61/1879), Intron (49/1486)

[ab]Main classes and sub classes of KEGG pathways defined on the KEGG website (https://www.genome.jp/kegg; br08901). [c]Names of enriched KEGG pathways (adjusted p-values < 0.05 and minimum gene counts > 4) together with KEGG IDs in paratheses. [d]Genetic regions - RS: regularly sequence (gene body + promoter + flanks), P: promoter (P250 + P1K + P5K), GB: gene body (intron + exon) - with gene ratio (# of DMGs in pathways / # of all DMGs).

Table S20. Liver DMGs L2:L1 - Enriched GO terms by ORA.

Root[a]	Region	# Terms	Top 5 enriched terms (terms with five lowest adjusted p-values)
**BP**	Exon	9	nervous system development (GO:0007399), regulation of small GTPase mediated signal transduction (GO:0051056), regulation of Ras protein signal transduction (GO:0046578), Ras protein signal transduction (GO:0007265), generation of neurons (GO:0048699)
	Gene body	58	regulation of small GTPase mediated signal transduction (GO:0051056), nervous system development (GO:0007399), Ras protein signal transduction (GO:0007265), Rho protein signal transduction (GO:0007266), regulation of Ras protein signal transduction (GO:0046578)
	Intron	46	regulation of small GTPase mediated signal transduction (GO:0051056), Ras protein signal transduction (GO:0007265), nervous system development (GO:0007399), Rho protein signal transduction (GO:0007266), regulation of Ras protein signal transduction (GO:0046578)
	P+GB	49	regulation of small GTPase mediated signal transduction (GO:0051056), nervous system development (GO:0007399), Ras protein signal transduction (GO:0007265), Rho protein signal transduction (GO:0007266), regulation of Ras protein signal transduction (GO:0046578)
	RS+GB	40	nervous system development (GO:0007399), regulation of small GTPase mediated signal transduction (GO:0051056), Ras protein signal transduction (GO:0007265), neurogenesis (GO:0022008), Rho protein signal transduction (GO:0007266)
**CC**	Exon	2	collagen trimer (GO:0005581), synapse part (GO:0044456)
	Gene body	16	synapse (GO:0045202), synapse part (GO:0044456), cation channel complex (GO:0034703), postsynaptic membrane (GO:0045211), synaptic membrane (GO:0097060)
	Intron	14	synapse (GO:0045202), synapse part (GO:0044456), neuron part (GO:0097458), cation channel complex (GO:0034703), ion channel complex (GO:0034702)
	P+GB	15	synapse (GO:0045202), synapse part (GO:0044456), postsynaptic membrane (GO:0045211), synaptic membrane (GO:0097060), cation channel complex (GO:0034703)
	RS+GB	15	synapse (GO:0045202), synapse part (GO:0044456), neuron part (GO:0097458), ion channel complex (GO:0034702), cation channel complex (GO:0034703)
**MF**	Exon	4	extracellular matrix structural constituent (GO:0005201), guanyl-nucleotide exchange factor activity (GO:0005085), Rho guanyl-nucleotide exchange factor activity (GO:0005089), Ras guanyl-nucleotide exchange factor activity (GO:0005088)
	Gene body	39	Ras GTPase binding (GO:0017016), small GTPase binding (GO:0031267), Rho GTPase binding (GO:0017048), guanyl-nucleotide exchange factor activity (GO:0005085), Rho guanyl-nucleotide exchange factor activity (GO:0005089)
	Intron	46	Ras GTPase binding (GO:0017016), small GTPase binding (GO:0031267), Rho GTPase binding (GO:0017048), guanyl-nucleotide exchange factor activity (GO:0005085), Ras guanyl-nucleotide exchange factor activity (GO:0005088)
	P+GB	36	Ras GTPase binding (GO:0017016), small GTPase binding (GO:0031267), Rho GTPase binding (GO:0017048), Rho guanyl-nucleotide exchange factor activity (GO:0005089), guanyl-nucleotide exchange factor activity (GO:0005085)
	RS+GB	32	Ras GTPase binding (GO:0017016), small GTPase binding (GO:0031267), Rho GTPase binding (GO:0017048), Ras guanyl-nucleotide exchange factor activity (GO:0005088), Rho guanyl-nucleotide exchange factor activity (GO:0005089)

[a]Root GO terms defined as CC (cellular component), BP (biological process), MF (molecular function).

Table S21. Liver DMGs L3:L1 - Enriched GO terms by ORA.

Root[a]	Region	# Terms	Top 5 enriched terms (terms with five lowest adjusted p-values)
**BP**	Gene body	55	regulation of small GTPase mediated signal transduction (GO:0051056), Ras protein signal transduction (GO:0007265), chemical synaptic transmission (GO:0007268), anterograde trans-synaptic signaling (GO:0098916), synaptic signaling (GO:0099536)
	Intron	42	chemical synaptic transmission (GO:0007268), anterograde trans-synaptic signaling (GO:0098916), synaptic signaling (GO:0099536), trans-synaptic signaling (GO:0099537), cell-cell adhesion (GO:0098609)
	P+GB	46	regulation of small GTPase mediated signal transduction (GO:0051056), Ras protein signal transduction (GO:0007265), chemical synaptic transmission (GO:0007268), anterograde trans-synaptic signaling (GO:0098916), synaptic signaling (GO:0099536)
	RS+GB	44	regulation of small GTPase mediated signal transduction (GO:0051056), nervous system development (GO:0007399), enzyme linked receptor protein signaling pathway (GO:0007167), chemical synaptic transmission (GO:0007268), anterograde trans-synaptic signaling (GO:0098916)
**CC**	Exon	1	collagen trimer (GO:0005581)
	Gene body	21	synapse (GO:0045202), synapse part (GO:0044456), neuron part (GO:0097458), presynapse (GO:0098793), postsynaptic membrane (GO:0045211)
	Intron	24	synapse (GO:0045202), synapse part (GO:0044456), neuron part (GO:0097458), presynapse (GO:0098793), postsynaptic membrane (GO:0045211)
	P+GB	19	synapse (GO:0045202), synapse part (GO:0044456), neuron part (GO:0097458), presynapse (GO:0098793), postsynaptic membrane (GO:0045211)
	RS+GB	15	synapse (GO:0045202), synapse part (GO:0044456), postsynaptic membrane (GO:0045211), synaptic membrane (GO:0097060), postsynapse (GO:0098794)
**MF**	Exon	5	extracellular matrix structural constituent (GO:0005201), Ras GTPase binding (GO:0017016), small GTPase binding (GO:0031267), Ras guanyl-nucleotide exchange factor activity (GO:0005088), Rho guanyl-nucleotide exchange factor activity (GO:0005089)
	Gene body	61	Ras GTPase binding (GO:0017016), small GTPase binding (GO:0031267), guanyl-nucleotide exchange factor activity (GO:0005085), Rho GTPase binding (GO:0017048), ephrin receptor activity (GO:0005003)
	Intron	58	ligand-gated ion channel activity (GO:0015276), ligand-gated channel activity (GO:0022834), ephrin receptor activity (GO:0005003), guanyl-nucleotide exchange factor activity (GO:0005085), Ras GTPase binding (GO:0017016)
	P+GB	43	small GTPase binding (GO:0031267), Ras GTPase binding (GO:0017016), Rho GTPase binding (GO:0017048), guanyl-nucleotide exchange factor activity (GO:0005085), ephrin receptor activity (GO:0005003)
	RS+GB	39	small GTPase binding (GO:0031267), Ras GTPase binding (GO:0017016), Rho GTPase binding (GO:0017048), ephrin receptor activity (GO:0005003), Rho guanyl-nucleotide exchange factor activity (GO:0005089)

[a]Root GO terms defined as CC (cellular component), BP (biological process), MF (molecular function).

Table S22. G&L DMGs - Enriched KEGG pathways by ORA.

Set	Main class[a]	Sub class[b]	KEGG pathway	KEGG ID	Genetic region (gene ratio) [c]
**L2:L1**	Environmental Information Processing	Signal transduction	**ErbB signaling pathway**	sasa04012	**RS+GB** (22/564), Intron (16/314)
**L3:L1**	Environmental Information Processing	Signal transduction	**ErbB signaling pathway**	sasa04012	**RS+GB** (22/550)
	Organismal Systems	Circulatory system	Adrenergic signaling in cardiomyocytes	sasa04261	RS+GB (37/550)

[ab]Main classes and sub classes of KEGG pathways defined on the KEGG website (https://www.genome.jp/kegg; br08901). [c]Names of enriched KEGG pathways (adjusted p-values < 0.05 and minimum gene counts > 4) together with KEGG IDs in paratheses. [d]Genetic regions - RS: regularly sequence (gene body + promoter + flanks), P: promoter (P250 + P1K + P5K), GB: gene body (intron + exon) - with gene ratio (# of DMGs in pathways / # of all DMGs).

Table S23. G&L DMGs L2:L1 - Enriched GO terms by ORA.

Root[a]	Region	# Terms	Top 5 enriched terms (terms with five lowest adjusted p-values)
**BP**	Gene body	3	homophilic cell adhesion via plasma membrane adhesion molecules (GO:0007156), cell-cell adhesion via plasma-membrane adhesion molecules (GO:0098742), regulation of small GTPase mediated signal transduction (GO:0051056)
**CC**	Intron	1	synapse (GO:0045202)
**MF**	Gene body	9	transmembrane receptor protein kinase activity (GO:0019199), Ras GTPase binding (GO:0017016), small GTPase binding (GO:0031267), transmembrane receptor protein tyrosine kinase activity (GO:0004714), Ras guanyl-nucleotide exchange factor activity (GO:0005088)
	P+GB	4	Ras GTPase binding (GO:0017016), small GTPase binding (GO:0031267), transmembrane receptor protein kinase activity (GO:0019199), transmembrane receptor protein tyrosine kinase activity (GO:0004714)

[a]Root GO terms defined as CC (cellular component), BP (biological process), MF (molecular function).

Table S24. G&L DMGs L3:L1 - Enriched GO terms by ORA.

Root[a]	Region	# Terms	Top 5 enriched terms (terms with five lowest adjusted p-values)
**BP**	Gene body	5	cell-cell adhesion via plasma-membrane adhesion molecules (GO:0098742), homophilic cell adhesion via plasma membrane adhesion molecules (GO:0007156), cell-cell adhesion (GO:0098609), neurotransmitter secretion (GO:0007269), signal release from synapse (GO:0099643)
	Intron	5	homophilic cell adhesion via plasma membrane adhesion molecules (GO:0007156), cell-cell adhesion via plasma-membrane adhesion molecules (GO:0098742), cell-cell adhesion (GO:0098609), neurotransmitter secretion (GO:0007269), signal release from synapse (GO:0099643)
	P+GB	5	cell-cell adhesion via plasma-membrane adhesion molecules (GO:0098742), homophilic cell adhesion via plasma membrane adhesion molecules (GO:0007156), neurotransmitter secretion (GO:0007269), signal release from synapse (GO:0099643), cell-cell adhesion (GO:0098609)
**CC**	Gene body	4	synapse (GO:0045202), synapse part (GO:0044456), neuron part (GO:0097458), presynapse (GO:0098793)
	Intron	4	synapse part (GO:0044456), synapse (GO:0045202), neuron part (GO:0097458), presynapse (GO:0098793)
	P+GB	3	synapse (GO:0045202), synapse part (GO:0044456), neuron part (GO:0097458)
	RS+GB	2	synapse (GO:0045202), synapse part (GO:0044456)
**MF**	Gene body	2	protein tyrosine kinase activity (GO:0004713), transmembrane receptor protein tyrosine kinase activity (GO:0004714)
	Intron	5	protein tyrosine kinase activity (GO:0004713), transmembrane receptor protein tyrosine kinase activity (GO:0004714), transmembrane receptor protein kinase activity (GO:0019199), extracellular ligand-gated ion channel activity (GO:0005230), steroid hormone receptor activity (GO:0003707)

[a]Root GO terms defined as CC (cellular component), BP (biological process), MF (molecular function).

Table S25. Enriched KEGG pathways with high tissue-specificity.

Tissue	Set	KEGG pathway	KEGG ID	Region (gene ratio)
Gonads	L2:L1	Other types of O-glycan biosynthesis	sasa00514	RS+GB (38/2818), P+GB (32/2311), Gene body (27/1958)
		Glycerolipid metabolism	sasa00561	RS+GB (43/2818), P+GB (37/2311), Gene body (31/1958)
		Insulin signaling pathway	sasa04910	P+GB (80/2311)
	L3:L1	Lysine degradation	sasa00310	Exon (15/545)
		Purine metabolism	sasa00230	Gene body (65/1898)
Liver	L2:L1	Tight junction	sasa04530	Intron (78/1443)
		ABC transporters	sasa02010	RS+GB (25/2652), P+GB (22/2123), Gene body (20/1822)
	L3:L1	ABC transporters	sasa02010	RS+GB (29/2725), P+GB (27/2226), Gene body (26/1879), Intron (20/1486)
		N-Glycan biosynthesis	sasa00510	P+GB (32/2226), Gene body (29/1879), Intron (25/1486)
		Fatty acid biosynthesis	sasa00061	P+GB (14/2226), Gene body (13/1879)

Table S26. Five DEGs with at least one DMC in gonad promoters of L3:L1.

Gene ID	Gene symbol	Gene description	LFC[a]	Adj p-val[b]	Region[c]	MDiff[d]	Q-val[e]
**106604849**	LOC106604849	arylsulfatase I-like	0.58	0.08	P250	32.81	2.16E-32
**106583543**	LOC106583543	myelin basic protein-like, transcript variant X4	0.51	0.08	P5K	-30.88	3.66E-05
**106601467**	LOC106601467	ras-related C3 botulinum toxin substrate 2	0.85	0.02	P5K	63.16	1.01E-22
**106570610**	LOC106570610	uncharacterized LOC106570610, transcript variant X2	0.38	0.05	P5K	43.02	1.35E-13
**106579347**	LOC106579347	guanylate cyclase 2G-like	4.92	0.02	P5K	-25.62	1.23E-03
					P5K	-32.07	2.29E-04

[a]Log2 fold changes of DEGs. [b]Adjusted p-values of DEGs. [c]Genetic regions of DMCs. [d]Methylation differences (%) of DMCs. [e]Q-values of DMCs.

Table S27. Number of common DMGs between L2:L1 and L3:L1.

Tissue	# common DMGs (unique)	Region	# common DMGs
**Gonads**	4336	Exon	745
		Exon150	27
		Intron	2403
		P250	37
		P1K	80
		P5K	387
		Flanks	889
**Liver**	2869	Exon	431
		Exon150	23
		Intron	1617
		P250	18
		P1K	65
		P5K	228
		Flanks	596
**G&L**	426	Exon	40
		Exon150	1
		Intron	254
		P250	2
		P1K	8
		P5K	37
		Flanks	89

Table S28. Top three common DMGs between L2:L1 and L3:L1 with the DMC counts in gonads.

Region	Gene ID	Gene symbol	Gene name	Count	Hypo	Hyper
**Exon**	100170847	homeobox protein HoxA2aa	homeobox protein HoxA2aa	6	6	0
	106569754	LOC106569754	dnaJ homolog subfamily B member 4-like	5	0	5
	106604600	LOC106604600	protocadherin-18-like	5	5	0
**Exon150**	106566321	LOC106566321	potassium voltage-gated channel subfamily A member 2-like	3	0	3
	106568430	LOC106568430	putative uncharacterized protein DDB_G0286901	2	0	2
	106591755	LOC106591755	nuclear pore complex protein Nup50-like	1	0	1
**Intron**	106573702	LOC106573702	cadherin-13-like	9	0	9
	106579565	LOC106579565	dipeptidyl aminopeptidase-like protein 6	9	4	5
	106577539	LOC106577539	neurexin-2-like	9	3	6
**P250**	106572512	LOC106572512	glutamate receptor ionotropic, NMDA 3A-like	2	2	0
	106582681	LOC106582681	dnaJ homolog subfamily C member 16-like	2	1	1
	106576969	DEAD (Asp-Glu-Ala-Asp) box polypeptide 43	DEAD-box helicase 43	1	1	0
**P1K**	106611715	LOC106611715	transmembrane protein 35-like	3	3	0
	106571557	plcb4	phospholipase C beta 4	3	0	3
	106578665	LOC106578665	protein scribble homolog	2	2	0
**P5K**	106566689	LOC106566689	MAM domain-containing protein 2-like	6	6	0
	106578448	LOC106578448	aristaless-related homeobox protein-like	6	2	4
	106560720	tsg101	tumor susceptibility 101	5	1	4
**Flanks**	106612344	LOC106612344	autophagy-related protein 16-1-like	6	2	4
	106608979	LOC106608979	E3 ubiquitin-protein ligase TRIM39-like	5	5	0
	106605378	LOC106605378	collagen alpha-1(XXVIII) chain-like	5	5	0

Table S29. Top three common DMGs between L2:L1 and L3:L1 with the DMC counts in liver.

Region	Gene ID	Gene symbol	Gene name	Count	Hypo	Hyper
**Exon**	106578606	LOC106578606	homeobox protein engrailed-2a-like	5	5	0
	106597047	LOC106597047	uncharacterized LOC106597047	5	0	5
	106583969	LOC106583969	abhydrolase domain-containing protein 8-like	4	0	4
**Exon150**	106569814	LOC106569814	guanine nucleotide exchange factor VAV3-like	2	2	0
	106595839	LOC106595839	uncharacterized LOC106595839	2	2	0
	106591680	LOC106591680	eukaryotic translation initiation factor 3 subunit A-like	1	1	0
**Intron**	106604876	LOC106604876	protocadherin gamma-C5-like	9	2	7
	106610060	LOC106610060	teneurin-3	7	1	6
	106571823	LOC106571823	zinc finger protein 2 homolog	6	0	6
**P250**	106572512	LOC106572512	glutamate receptor ionotropic, NMDA 3A-like	4	4	0
	106572076	myh7b	myosin heavy chain 7B	3	2	1
	100196355	NADH dehydrogenase 1 alpha subcomplex subunit 4-like 2	NADH dehydrogenase 1 alpha subcomplex subunit 4-like 2	2	0	2
**P1K**	106600623	LOC106600623	EMILIN-3-like	3	0	3
	106577589	ddit4l	DNA damage inducible transcript 4 like	2	0	2
	106607316	LOC106607316	tubulin gamma-1 chain	2	2	0
**P5K**	106586627	LOC106586627	vang-like protein 1	5	0	5
	106570019	LOC106570019	zinc finger MYM-type protein 4-like	4	4	0
	106565580	cav3	caveolin 3	4	0	4
**Flanks**	106568695	LOC106568695	probable G-protein coupled receptor 158	4	0	4
	106563084	LOC106563084	multiple C2 and transmembrane domain-containing protein 1-like	4	0	4
	106567661	LOC106567661	SH2B adapter protein 2-like	4	0	4

Table S30. Top three common DMGs between L2:L1 and L3:L1 with the DMC counts in G&L.

Region	Gene ID	Gene symbol	Gene name	Count	Hypo	Hyper
**Exon**	106578606	LOC106578606	homeobox protein engrailed-2a-like	5	5	0
	106565559	efcab12	EF-hand calcium binding domain 12	2	0	2
	106578193	LOC106578193	uncharacterized LOC106578193	2	0	2
**Flanks**	106567661	LOC106567661	SH2B adapter protein 2-like	6	0	6
	106577054	LOC106577054	protein tweety homolog 1-like	3	0	3
	106605378	LOC106605378	collagen alpha-1(XXVIII) chain-like	3	3	0
**Exon150**	106591755	LOC106591755	nuclear pore complex protein Nup50-like	1	0	1
**Intron**	106577697	LOC106577697	transmembrane protein 256-like	4	4	0
	106564814	LOC106564814	egl nine homolog 1-like	4	0	4
	106585546	LOC106585546	synapsin-1-like	3	0	3
**P1K**	106580048	LOC106580048	mothers against decapentaplegic homolog 2-like	1	0	1
	106603600	LOC106603600	misshapen-like kinase 1	1	0	1
	106577444	LOC106577444	leucine-rich repeat and calponin homology domain-containing protein 4-like	1	1	0
**P250**	106572512	LOC106572512	glutamate receptor ionotropic, NMDA 3A-like	4	4	0
	100196336	COMM domain containing 7	COMM domain containing 7	1	1	0
**P5K**	106569517	LOC106569517	semaphorin-6B-like	2	0	2
	106603277	LOC106603277	seizure protein 6-like	2	0	2
	106599732	LOC106599732	meprin A subunit beta-like	2	0	2

Table S31. Predicted transcription factors for *glutamate receptor ionotropic, NMDA 3A-like*.

Motif ID	Motif score	Distance from TSS	Motif length	Motif strand	Matched sequence	P-value	Motif start	Motif stop	TF
**6084**	13.37	-116	9	+	CGCGCTCGC	1.20E-05	156082322	156082330	HDAC2
**6073**	9.02	-115	9	-	AGCGAGCGC	5.87E-05	156082321	156082329	LRF
**1445**	8.67	-114	11	+	CGCGCTCGCTC	7.40E-05	156082320	156082330	ZFP206

Table S32. List of DEGs for L2:L1.

File: Dataset_01_DEG_L2L1.xlsx (provided in Excel format)

Sheets: Gonad (DEGs), Liver (DEGs), G&L (DEGs), Gonad (all genes), Liver (all genes), G&L (all genes)

Fields:

Gene ID	Entrez Gene ID
Base mean	Output of DESeq2
Log2 fold change	Output of DESeq2
P-value	Output of DESeq2
Adjusted p-value	Output of DESeq2
Gene symbol	Gene symbol
Gene name	Gene name

Table S33. List of DEGs for L3:L1.

File: Dataset_02_DEG_L3L1.xlsx (provided in Excel format)

Sheets: Gonad (DEGs), Liver (DEGs), G&L (DEGs), Gonad (all genes), Liver (all genes), G&L (all genes)

Fields:

Gene ID	Entrez Gene ID
Base mean	Output of DESeq2
Log2 fold change	Output of DESeq2
P-value	Output of DESeq2
Adjusted p-value	Output of DESeq2
Gene symbol	Gene symbol
Gene name	Gene name

Table S34. List of DMCs and CpG sites (sig CpGs) with q-value < 0.01 for L2:L1.

File: Dataset_03_DMC_L2L1.xlsx (provided in Excel format)

Sheet: Gonad (DMCs), Liver (DMCs), G&L (DMCs), Gonad (sig CpGs), Liver (sig CpGs), G&L (sig CpGs)

Fields:

Chromosome	Salmon chromosome name
Start	1-base start
End	1-base end
Strand	+/-
Region	Intron, Exon, P250, P1K, P5K, Flanks
Q value	Logistic regression and SLIM, < 0.01
Mdiff	Methylation difference
abs(Mdiff)	Absolute methylation difference
Gene ID	Entrez Gene ID
Gene symbol	Gene symbol
Gene name	Gene name
RefSeq id	RefSeq ID

Table S35. List of DMCs and CpG sites (sig CpGs) with q-value < 0.01 for L3:L1.

File: Dataset_04_DMC_L3L1.xlsx (provided in Excel format)

Sheet: Gonad (DMCs), Liver (DMCs), G&L (DMCs), Gonad (sig CpGs), Liver (sig CpGs), G&L (sig CpGs)

Fields:

Chromosome	Salmon chromosome name
Start	1-base start
End	1-base end
Strand	+/-
Region	Intron, Exon, P250, P1K, P5K, Flanks
Q value	Logistic regression and SLIM, < 0.01
Mdiff	Methylation difference
abs(Mdiff)	Absolute methylation difference
Gene ID	Entrez Gene ID
Gene symbol	Gene symbol
Gene name	Gene name
RefSeq id	RefSeq ID

Table S36. List of DMCs by region in gonad L2:L1.

File: Dataset_05_DMC_Region_Gonad_L2L1.xlsx (provided in Excel format)

Sheet: Intron, Exon, P250, P1K, P5K, Flanks

Fields:

Chromosome	Salmon chromosome name
Start	1-base start
End	1-base end
Strand	+/-
Region	Intron, Exon, P250, P1K, P5K, Flanks
Q value	Logistic regression and SLIM, < 0.01
Mdiff	Methylation difference
abs(Mdiff)	Absolute methylation difference
Gene ID	Entrez Gene ID
Gene symbol	Gene symbol
Gene name	Gene name
RefSeq id	RefSeq ID

Table S37. List of DMCs by region in gonad L3:L1.

File: Dataset_06_DMC_Region_Gonad_L3L1.xlsx (provided in Excel format)

Sheet: Intron, Exon, P250, P1K, P5K, Flanks

Fields:

Chromosome	Salmon chromosome name
Start	1-base start
End	1-base end
Strand	+/-
Region	Intron, Exon, P250, P1K, P5K, Flanks
Q value	Logistic regression and SLIM, < 0.01
Mdiff	Methylation difference
abs(Mdiff)	Absolute methylation difference
Gene ID	Entrez Gene ID
Gene symbol	Gene symbol
Gene name	Gene name
RefSeq id	RefSeq ID

Table S38. List of DMGs for L2:L1.

File: Dataset_07_DMG_L2L1.xlsx (provided in Excel format)

Sheets: Gonad, Liver, G&L

Fields:

Gene ID	Entrez Gene ID
Total	Total count of DMCs
GB	Total count of DMCs in gene body
Exon	Count of DMCs in exon
Intron	Count of DMCs in intron
P	Total count of DMCs in promoter
P250	Count of DMCs in promoter (1 - 250)
P1K	Count of DMCs in promoter (251 - 1K)
P5K	Count of DMCs in Promoter (1001 - 5K)
Gene symbol	Gene name
Gene description	Description of gene
RefSeq IDs	RefSeq IDs linked to the gene ID

Table S39. List of DMGs for L3:L1.

File: Dataset_08_DMG_L3L1.xlsx (provided in Excel format)

Sheets: Gonad, Liver, G&L

Fields:

Gene ID	Entrez Gene ID
Total	Total count of DMCs
GB	Total count of DMCs in gene body
Exon	Count of DMCs in exon
Intron	Count of DMCs in intron
P	Total count of DMCs in promoter
P250	Count of DMCs in promoter (1 - 250)
P1K	Count of DMCs in promoter (251 - 1K)
P5K	Count of DMCs in Promoter (1001 - 5K)
Gene symbol	Gene name
Gene description	Description of gene
RefSeq IDs	RefSeq IDs linked to the gene ID

Table S40. List of the DEGs that contain at least one DMC in gonads.

File: Dataset_09_DMGxDEG_Gonad.xls (provided in Excel format)
x

Sheets: L2L1 gonad (DMG) x gonad (DEG), L3L1 gonad (DMG) x gonad (DEG),
L2L1 gonad (DMG) x liver (DEG), L3L1 gonad (DMG) x liver (DEG),
L2L1 gonad (DMG) x G&L (DEG), L3L1 gonad (DMG) x G&L (DEG)

Fields:

Gene ID	Entrez Gene ID
Gene symbol	Gene symbol
Gene description	Gene name
Log2 FC	Output of DESeq2
Adj p-value	Output of DESeq2
Region	Intron, Exon, P250, P1K, P5K, Flanks
Mdiff	Methylation difference
Q value	Logistic regression and SLIM, < 0.01

Table S41. List of the DEGs that contain at least one DMC in liver.

File: Dataset_10_DMGxDEG_Liver.xlsx (provided in Excel format)

Sheets: L2L1 liver (DMG) x liver (DEG), L3L1 liver (DMG) x liver (DEG),
L2L1 liver (DMG) x gonad (DEG), L3L1 liver (DMG) x gonad (DEG),
L2L1 liver (DMG) x G&L (DEG), L3L1 liver (DMG) x G&L (DEG)

Fields:

Gene ID	Entrez Gene ID
Gene symbol	Gene symbol
Gene description	Gene name
Log2 FC	Output of DESeq2
Adj p-value	Output of DESeq2
Region	Intron, Exon, P250, P1K, P5K, Flanks
Mdiff	Methylation difference
Q value	Logistic regression and SLIM, < 0.01

Table S42. List of the DEGs that contain at least one DMC for G&L.

File: Dataset_11_DMGxDEG_GxL.xlsx (provided in Excel format)

Sheets: L2L1 G&L (DMG) x G&L (DEG), L3L1 G&L (DMG) x G&L (DEG),
L2L1 G&L (DMG) x gonad (DEG), L3L1 G&L (DMG) x gonad (DEG),
L2L1 G&L (DMG) x liver (DEG), L3L1 G&L (DMG) x liver (DEG)

Fields:

Gene ID	Entrez Gene ID
Gene symbol	Gene symbol
Gene description	Gene name
Log2 FC	Output of DESeq2
Adj p-value	Output of DESeq2
Region	Intron, Exon, P250, P1K, P5K, Flanks
Mdiff	Methylation difference
Q value	Logistic regression and SLIM, < 0.01

Table S43. List of the common DMCs between L2:L1 and L3:L1.

File: Dataset_12_Common_DMC.xlsx (provided in Excel format)

Sheets: Gonad (DMCs), Liver (DMCs), GandL (DMCs)

Note: Common DMCs are defined as an intersection of DMCs between L2:L1 and L3:L1. In addition, hyper and hypo methylation must be matched between L2:L1 and L3:L1.

Fields:

Chromosome	Salmon chromosome name
Start	1-base start
End	1-base end
Strand	+/-
Region	Intron, Exon, P250, P1K, P5K, Flanks
Q value (L2L1)	Logistic regression and SLIM, < 0.01
Mdiff (L2L1)	Methylation difference
Q value (L3L1)	Logistic regression and SLIM, < 0.01
Mdiff (L3L1)	Methylation difference
Gene ID	Entrez Gene ID
Gene symbol	Gene symbol
Gene name	Gene name

Table S44. List of the common DMGs between L2:L1 and L3:L1.

File: Dataset_13_Common_DMG.xlsx (provided in Excel format)

Sheets: Gonad (DMGs), Liver (DMGs), GandL (DMGs)

Note: Common DMGs are defined as DMGs that have at least one common DMC. Common DMCs are defined as an intersection of DMCs between L2:L1 and L3:L1. In addition, hyper and hypo methylation must be matched between L2:L1 and L3:L1.

Fields:

Region	Intron, Exon, P250, P1K, P5K, Flanks
Gene ID	Entrez Gene ID
Gene symbol	Gene symbol
Gene name	Gene name
Count	Count of DMCs
Hypo	Count of hypomethylated DMCs
Hyper	Count of hypermethylated DMCs

References

Hamre, K., N. H. Sissener, E. J. Lock, P. A. Olsvik, M. Espe, B. E. Torstensen, J. Silva, J. Johansen, R. Waagbo, and G. I. Hemre. 2016. 'Antioxidant nutrition in Atlantic salmon (Salmo salar) parr and post-smolt, fed diets with high inclusion of plant ingredients and graded levels of micronutrients and selected amino acids', *PeerJ*, 4: e2688.

Hemre, G. I., E. J. Lock, P. A. Olsvik, K. Hamre, M. Espe, B. E. Torstensen, J. Silva, A. C. Hansen, R. Waagbo, J. S. Johansen, M. Sanden, and N. H. Sissener. 2016. 'Atlantic salmon (Salmo salar) require increased dietary levels of B-vitamins when fed diets with high inclusion of plant based ingredients', *PeerJ*, 4: e2493.

Kadri, Sunil, Neil B. Metcalfe, Felicity A. Huntingford, John E. Thorpe, and David F. Mitchell. 1997. 'Early morphological predictors of maturity in one-sea-winter Atlantic salmon', *Aquaculture International*, 5: 41-50.

NRC, (National Research Council). 2011. *Nutrient requirements of fish and shrimp* (National academies press: Washington, DC).

Taylor, J. F., L. M. Vera, C. De Santis, E. J. Lock, M. Espe, K. H. Skjaerven, D. Leeming, J. Del Pozo, J. Mota-Velasco, H. Migaud, K. Hamre, and D. R. Tocher. 2019. 'The effect of micronutrient supplementation on growth and hepatic metabolism in diploid and triploid Atlantic salmon (Salmo salar) parr fed a low marine ingredient diet', *Comp Biochem Physiol B Biochem Mol Biol*, 227: 106-21.
